# Metabolomics Tools Assisting Classic Screening Methods in Discovering New Antibiotics from Mangrove Actinomycetia in Leizhou Peninsula

**DOI:** 10.3390/md19120688

**Published:** 2021-12-01

**Authors:** Qin-Pei Lu, Yong-Mei Huang, Shao-Wei Liu, Gang Wu, Qin Yang, Li-Fang Liu, Hai-Tao Zhang, Yi Qi, Ting Wang, Zhong-Ke Jiang, Jun-Jie Li, Hao Cai, Xiu-Jun Liu, Hui Luo, Cheng-Hang Sun

**Affiliations:** 1 Department of Microbial Chemistry, Institute of Medicinal Biotechnology, Chinese Academy of Medical Sciences & Peking Union Medical College, Beijing 100050, China; qinpei89@hotmail.com (Q.-P.L.); liushaowei3535@163.com (S.-W.L.); gangwu@aliyun.com (G.W.); yangqin@imb.pumc.edu.cn (Q.Y.); LiuLiFang@imb.pumc.edu.cn (L.-F.L.); tingwang0707@imb.pumc.edu.cn (T.W.); jiangzhongke@126.com (Z.-K.J.); 2 Beijing Key Laboratory of Antimicrobial Agents, Institute of Medicinal Biotechnology, Chinese Academy of Medical Sciences & Peking Union Medical College, Beijing 100050, China; 3 The Key Lab of Zhanjiang for R&D Marine Microbial Resources in the Beibu Gulf Rim, Marine Biomedical Research Institute, Guangdong Medical University, Zhanjiang 524023, China; huangym@gdmu.edu.cn (Y.-M.H.); taohaizhang33@163.com (H.-T.Z.); qiyi7272@gdmu.edu.cn (Y.Q.); jjleeee@gdmu.edu.cn (J.-J.L.); 4 Marine Biomedical Research Institute of Guangdong Zhanjiang, Zhanjiang 524023, China; 5 Department of Oncology, Institute of Medicinal Biotechnology, Chinese Academy of Medical Sciences & Peking Union Medical College, Beijing 100050, China; caihao@imb.pumc.edu.cn (H.C.); Liuxiujun2000@imb.pumc.edu.cn (X.-J.L.)

**Keywords:** Leizhou Peninsula, mangrove soil, actinomycetia, diversity, antimicrobial activity, secondary metabolites, dereplication, metabolomics tools, trioxacarcins

## Abstract

Mangrove actinomycetia are considered one of the promising sources for discovering novel biologically active compounds. Traditional bioactivity- and/or taxonomy-based methods are inefficient and usually result in the re-discovery of known metabolites. Thus, improving selection efficiency among strain candidates is of interest especially in the early stage of the antibiotic discovery program. In this study, an integrated strategy of combining phylogenetic data and bioactivity tests with a metabolomics-based dereplication approach was applied to fast track the selection process. A total of 521 actinomycetial strains affiliated to 40 genera in 23 families were isolated from 13 different mangrove soil samples by the culture-dependent method. A total of 179 strains affiliated to 40 different genera with a unique colony morphology were selected to evaluate antibacterial activity against 12 indicator bacteria. Of the 179 tested isolates, 47 showed activities against at least one of the tested pathogens. Analysis of 23 out of 47 active isolates using UPLC-HRMS-PCA revealed six outliers. Further analysis using the OPLS-DA model identified five compounds from two outliers contributing to the bioactivity against drug-sensitive *A. baumannii*. Molecular networking was used to determine the relationship of significant metabolites in six outliers and to find their potentially new congeners. Finally, two *Streptomyces* strains (M22, H37) producing potentially new compounds were rapidly prioritized on the basis of their distinct chemistry profiles, dereplication results, and antibacterial activities, as well as taxonomical information. Two new trioxacarcins with keto-reduced trioxacarcinose B, gutingimycin B (**16**) and trioxacarcin G (**20**), together with known gutingimycin (**12**), were isolated from the scale-up fermentation broth of *Streptomyces* sp. M22. Our study demonstrated that metabolomics tools could greatly assist classic antibiotic discovery methods in strain prioritization to improve efficiency in discovering novel antibiotics from those highly productive and rich diversity ecosystems.

## 1. Introduction

Highly effective antibiotics have been waning since they were introduced into the clinic to treat infectious diseases more than 75 years ago. Antibacterial-resistant bacteria account for approximately 700,000 deaths each year worldwide [1]. The near-empty pharmaceutical pipeline for new antibiotics has now reached alarming levels. In 2017, the World Health Organization (WHO) released a list of antibiotic-resistant “priority pathogens” that posed the greatest threat to human health for the first time, reminding that we were stepping into the post-antibiotic era [2]. Therefore, the urgent need for new antimicrobial agents has augmented scientists’ interest in discovering new antibiotics.

Natural products have a profound role in drug discovery and development [3]. Between 1981 and 2019, natural products and their derivatives accounted for 55% of the 162 new antibacterial chemical entities [4]. The strains in the class *Actinomycetia* (former class *Actinobacteria*) [5], especially species of the genus *Streptomyces*, are known as a rich source of novel antibiotics [6,7,8,9]. However, finding novel antibiotics from in-depth investigated terrestrial microorganisms has become more challenging. Researchers have been exploring untapped sources of biodiversity for novel pharmaceutical compounds, such as the deep sea [10], deserts [11], polar areas [12], and mangroves [13,14]. Because of its high moisture, high salinity, and hypoxia-tolerant ecosystem in the intertidal zones, the mangrove ecosystem is unique and contains a wealth of undiscovered bacteria and natural products, making it a focus area for bioactive natural product discovery [15]. To the best of our knowledge, very little research has been carried out on bioprospecting of Leizhou Peninsula mangrove actinomycetia. Only a few reports describing actinomycetia isolated from this ecosystem have been published so far, and no research on prioritizing the actinomycetial strains and discovering their secondary metabolites has been carried out [16,17,18].

The selection of strain candidates from large strain collections using traditional bioactivity screening and/or taxonomy-based methods usually result in the reisolation of known compounds [19,20]. Nowadays, exploring new bioactive compounds from microbial strains has moved toward integrated strategies, which combine phylogenetic data and bioactivity tests with dereplication approaches for rapid identification of known bioactive metabolites. Dereplication using chromatographic and spectroscopic methods and database searches can save time and avoid repetitive work during natural product discovery programs. Recently, a new dereplication method was developed by Tim Bugni using chemical dereplication coupled with metabolomics tools [21]. By incorporating metabolomics approaches, dereplication can focus on chemically diverse bacterial extracts from the bioactive strains, and this new method has shown to be effective in discovering putative new bioactive compounds and is frequently employed in microbial drug discovery programs.

Metabolomics is the comprehensive analysis of small molecule metabolites in a biological system to reflect the phenotype of its genomic, transcriptomic, and proteomic networks, providing insight into the biological functions [22]. In the past, metabolomics was mainly used to investigate primary metabolites, such as nucleotides, amino acids, and lipids. However, the majority of modern metabolomics coupled with molecular networking is readily applied to secondary metabolites discovery for new natural products. Combined principal component analysis (PCA) with LC/MS-based metabolomics is an efficient analytical tool to differentiate the bacterial strains based on their LC/MS profiles [23,24]. The strains producing similar secondary metabolites are clustered together, whereas those with different metabolites are separated. This new approach could significantly accelerate the bioassay-guided selection for chemically distinct strains that might yield novel bioactive secondary metabolites [25,26,27]. In addition, orthogonal partial least squares-discriminant analysis (OPLS-DA) is a supervised multivariate analysis that targets bioactive metabolites between the active and inactive groups to give information about the chemical composition of selected active extracts before isolation [19,20,28,29]. Molecular networking, one of the main analysis tools in the GNPS platform, creates structured networking based on MS/MS similarity to reflect the molecular diversity in the extracts. It has been proven to be successful for effective chemical dereplication and novel metabolite discovery [30,31,32].

In the present study, we employed integrated strategies in prioritizing the actinomycetial strains from the underexplored mangrove soil in Leizhou Peninsula. Actinomycetial strains were isolated using the culture-dependent method and phylogenetically characterized based on 16S rRNA gene sequencing. The selected actinomycetial strains were further subjected to antibacterial assays followed by metabolomics analysis, such as PCA, OPLS-DA, and molecular networking. The obtained data were integrated to prioritize the strains for follow-up chemical isolation and structural identification work of putative new compounds.

## 2. Results

### 2.1. Strain Isolation and Phylogenetic Identification

**Figure 1:**
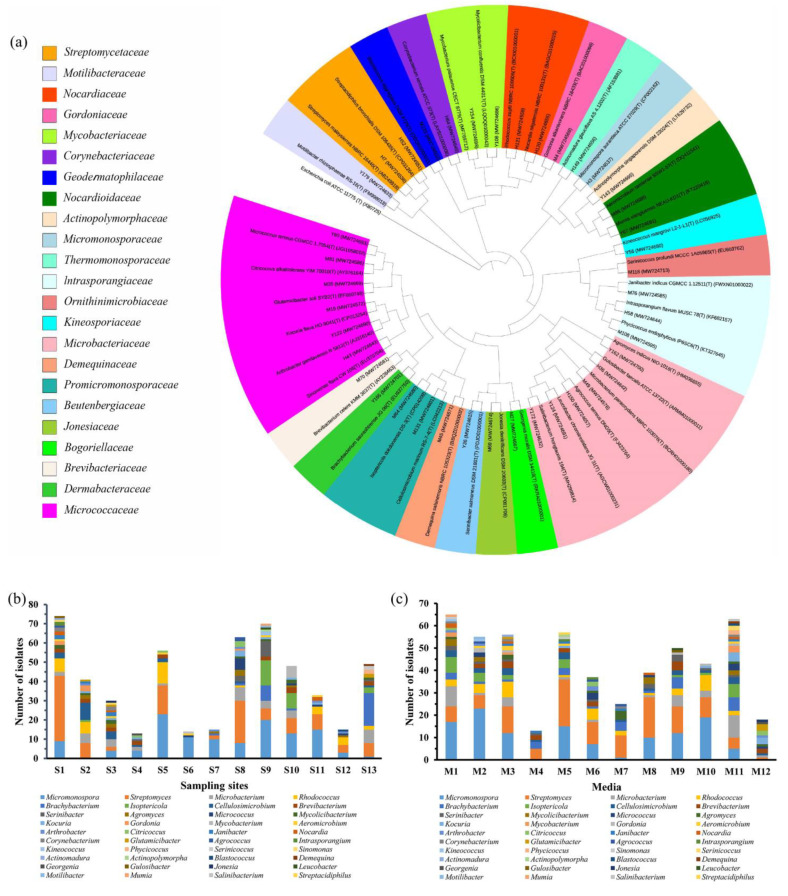
The distribution of cultivable actinomycetial strains isolated from mangrove soil in Leizhou Peninsula. (**a**) Phylogenetic tree (16S rRNA gene) obtained by neighbor-joining analysis of 40 isolates from each genus and their closely related type strains; *Escherichia coli* was used as an outgroup; (**b**) genera distribution in different sampling sites; (**c**) genera distribution according to the culture media used for the isolation.

A total of 521 actinomycetial strains were isolated by 12 different agar media (Table S1). Partial 16S rRNA gene sequence analysis identified these strains as the following genera: *Micromonospora* (121 strains), *Streptomyces* (116), *Microbacterium* (36), *Rhodococcus* (35), *Brachybacterium* (28), *Isoptericola* (25), *Cellulosimicrobium* (21), *Brevibacterium* (17), *Serinibacter* (13), *Mycolicibacterium* (10), *Micrococcus* (10), *Agromyces* (10), *Kocuria* (9), *Mycobacterium* (6), *Gordonia* (6), *Aeromicrobium* (5), *Arthrobacter* (5), *Citricoccus* (5), *Janibacter* (5), *Nocardia* (5), *Corynebacterium* (4), *Glutamicibacter* (4), *Agrococcus* (3), *Intrasporangium* (2), *Kineococcus* (2), *Phycicoccus* (2), *Sinomonas* (2), *Serinicoccus* (2), *Actinomadura* (1), *Actinopolymorpha* (1), *Blastococcus* (1), *Demequina* (1), *Georgenia* (1), *Gulosibacter* (1), *Jonesia* (1), *Leucobacter* (1), *Motilibacter* (1), *Mumia* (1), *Salinibacterium* (1), and *Streptacidiphilus* (1). These strains were affiliated to 40 different genera in 23 families (Figure 1a, Tables S2 and S3). The genera *Micromonospora* (23.2%) and *Streptomyces* (22.3%) were dominant, followed by *Microbacterium* (6.9%), *Rhodococcus* (6.7%), *Brachybacterium* (5.4%), and *Isoptericola* (4.8%). Additionally, 12 genera were represented by only one isolate. The distribution of the 521 actinomycetial strains from 13 different mangrove soil samples is shown in Figure 1b and Table S2. Sample 1 gave the highest diversity and abundance (74 isolates in 17 genera). Samples 3, 8, and 9 shared the second-highest diversity (15 genera), followed closely by sample 13 (14 genera) and sample 2 (13 genera). Two samples collected from Dongsong island showed the lowest diversity (sample 6, 4 genera; sample 7, 5 genera). With respect to the medium composition, M1 yielded the highest recovery rate (12.5%), with 65 isolates representing 18 different genera (Figure 1c and Table S3). A similar result showing higher diversity (63 isolates in 23 genera) was obtained from the modified M1 medium (M11) by the addition of kelp (Table S1). M5 displayed the third-best recovery rates (10.9%) with 57 isolates in 15 genera and showed the best recovery rate of *Streptomyces* (21 isolates). M4 yielded the lowest number and diversity of isolates (13 isolates in 6 genera). Interestingly, M12 (18 isolates in 12 genera), the amended M4 medium with the addition of coconut juice (Table S1), showed a higher number and diversity of isolates than M4 (13 isolates in 6 genera).

### 2.2. Antibacterial Assay

**Figure 2:**
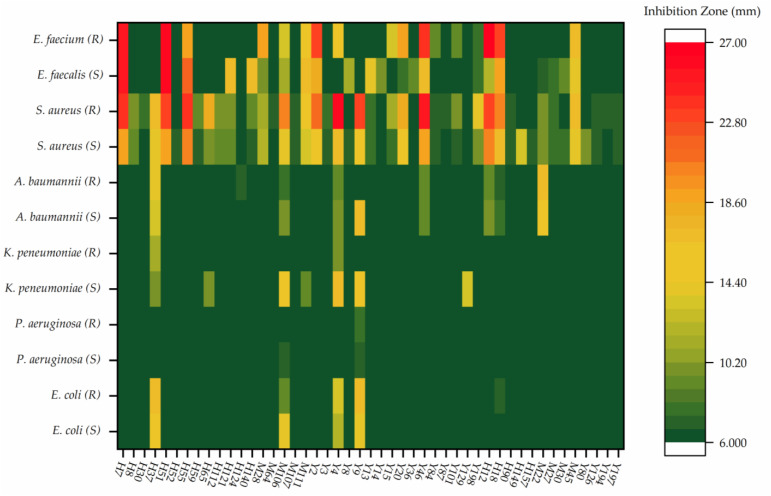
The antibacterial spectra of 47 strains against indicator bacteria (6 mm, no inhibitory activity; *S*, sensitive; *R*, drug resistant; *E. coli*, *Escherichia coli*; *P. aeruginosa, Pseudomonas aeruginosa*; *K. pneumoniae*, *Klebsiella pneumoniae*; *A. baumannii*, *Acinetobacter baumannii*; *S. aureus*, *Staphylococcus aureus*; *E. faecalis*, *Enterococcus faecalis*; *E. faecium*, *Enterococcus faecium*).

The antibacterial assay was used as one effective way to exclude the inactive strains and select the candidates for metabolomics analysis. Out of 521 actinomycetial strains from 13 different mangrove soil samples, 179 strains affiliated to 40 different genera with a unique colony morphology were selected to evaluate the antibacterial activities against six sets of indicator bacteria; each set contained one drug-sensitive and one drug-resistant pathogen (*Enterococcus faecium*, *Staphylococcus aureus*, *Klebsiella pneumoniae*, *Acinetobacter baumannii*, *Pseudomonas aeruginosa*, and *Enterobacter species*). Of the 179 strains, 47 exhibited antagonistic activity against at least one of the tested pathogens. These bioactive strains were distributed in 13 genera, including *Streptomyces* (29), *Micromonospora* (4), *Micrococcus* (3), *Rhodococcus* (2), *Brevibacterium* (1), *Demequina* (1), *Actinomadura* (1), *Georgenia* (1), *Gordonia* (1), *Isoptericola* (1), *Microbacterium* (1), *Streptacidiphilus* (1), and *Serinibacter* (1) (Table S4). The antibacterial spectra of the 47 isolates against indicator bacteria are shown in Figure 2. Of the 47 strains, 35 were active only against the Gram-positive bacteria, while two isolates (*Streptomyces* sp. H124 and *Streptomyces* sp. Y129) were active only against the Gram-negative bacteria. In addition, ten strains showed inhibitory activities against both the Gram-positive and Gram-negative bacteria. Interestingly, isolate M45, a rare actinomycetial strain affiliated to genus *Demequina*, showed moderate activities against all four Gram-positive bacterial strains tested. Thirty-seven isolates showed anti-MRSA activity, accounting for the highest proportion of the total active strains (78.7%). Twenty-three strains showing high anti-MRSA activity (diameter of inhibition zone ≥ 10 mm) were selected for subsequent metabolomics analyses (Table 1).

### 2.3. Strain Prioritization by Metabolomics

**Figure 3:**
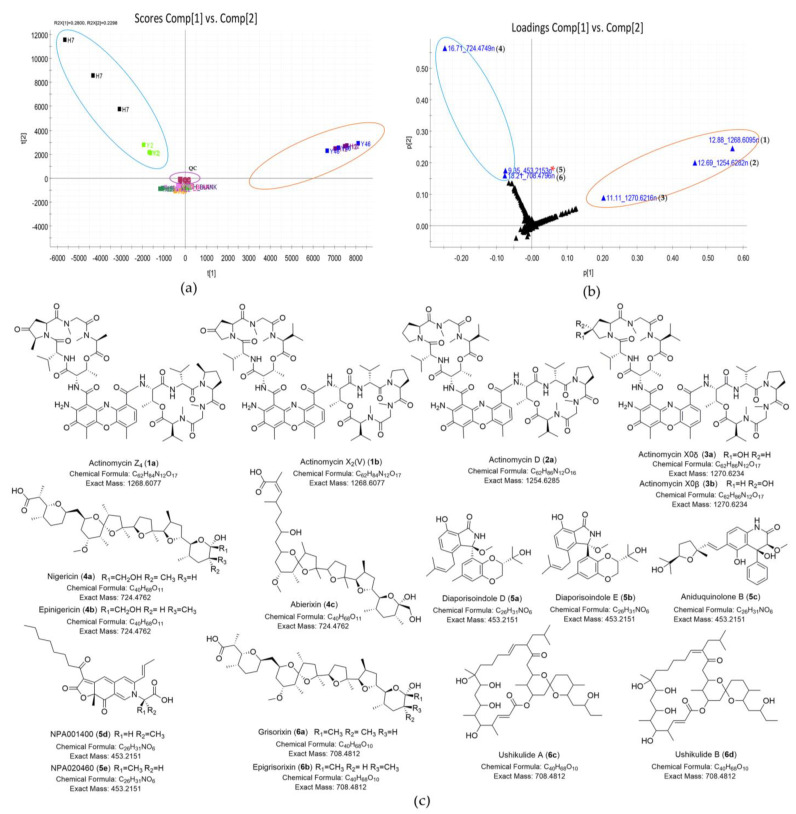
Discovery of natural products unique to four outlier strains (*Streptomyces* sp. Y46, *Streptomyces* sp. H12, *Streptomyces* sp. H7, and *Streptomyces* sp. Y2). (**a**) Scores plot (PC1 vs. PC2) of 23 extracts; (**b**) loadings plot (PC1 vs. PC2) of 23 extracts; the loadings plot showed compounds **1**–**3** were unique to *Streptomyces* sp. H12 and *Streptomyces* sp. Y46, and compounds **4**–**6** were special to *Streptomyces* sp. H7 and *Streptomyces* sp. Y2; 9.35_453.2153n* (**5**): false positive; (**c**) The structures of putative metabolites for compounds **1**–**6** from four outlier strains by database dereplication.

The ethyl acetate extracts of 23 isolates shown in Table 1 were subjected to metabolomics analysis using UPLC-HRMS-PCA. PCA, an unsupervised statistical analysis method, was used to identify differing chemical features found in the outlying strains and to prioritize the strains that produced unique secondary metabolites. After UPLC-MS/MS data acquisition and processing, a total of 6544 features (RT-*m/z* pairs) and 6 principal components were generated by PCA analysis, giving the R^2^ (goodness of fit) and Q^2^ (predictability) values of 0.82 and 0.70, respectively. After analyzing the scores plot (PC1 vs. PC2), the quality control (QC) samples (purple circle) clustered and were close to the center of the scores plot (Figure 3a), indicating good reproducibility and stability of the system. Four predominant outliers, *Streptomyces* sp. H7, *Streptomyces* sp. Y2, *Streptomyces* sp. H12, and *Streptomyces* sp. Y46, were observed, suggesting their chemical uniqueness from the main groups of samples in the scores plot. *Streptomyces* sp. H7 and *Streptomyces* sp. Y2 were located in the same quadrant, indicating similarities in their secondary metabolite profiles. Similarly, the metabolite profiles of *Streptomyces* sp. H12 and *Streptomyces* sp. Y46 were also alike. The loadings plot (Figure 3b) was geometrically related to the scores plot and described the variance observed in the scores plot, so it could be used to identify compounds that caused a clustered group to separate. From the loadings plot, three outlying metabolites, 12.88_1268.6095n (n: neutral mass) (**1**), 12.69_1254.6282n (**2**), and 11.11_1270.6216n (**3**), were significant contributors to the group of *Streptomyces* sp. H12 and *Streptomyces* sp. Y46. They were putatively assigned as actinomycin-type antibiotics through dereplication with the Natural Products Atlas (NPAtlas) v19_12 database [33] and StreptomeDB v3.0 database [34] and further confirmed as the actinomycins by comparisons of their ultraviolet (UV) spectra with previously published data (Figure 3c, Table 2, and Figure S1) [35,36]. Comparison with the other extracts revealed that the outlying feature 16.71_724.4749n (**4**) was abundant in *Streptomyces* sp. H7 and present in *Streptomyces* sp. Y2 with lower peak intensities (Figure S2). The predicted molecular formula C_40_H_68_O_11_ was tentatively identified as nigericin, epinigericin, or abierixin by dereplication. Abierixin was first excluded because it had UV absorptions at 200–400 nm but the feature 16.71_724.4749n (**4**) lacked UV absorptions [37]. Meanwhile, the MS/MS fragment pattern (Figure S3) and UV characterization of 16.71_724.4749n (**4**) strongly suggested that it was most likely to be either nigericin or epinigericin [38,39]. Another feature 18.21_708.4796n (**6**) was putatively elucidated as 22-member macrolides (ushikulide A or B) or nigericin-type polyethers (grisorixin or epigrisorixin); all were previously isolated from *Streptomyces* spp. (Table 2, Figure 3c) [40,41,42].

**Figure 4:**
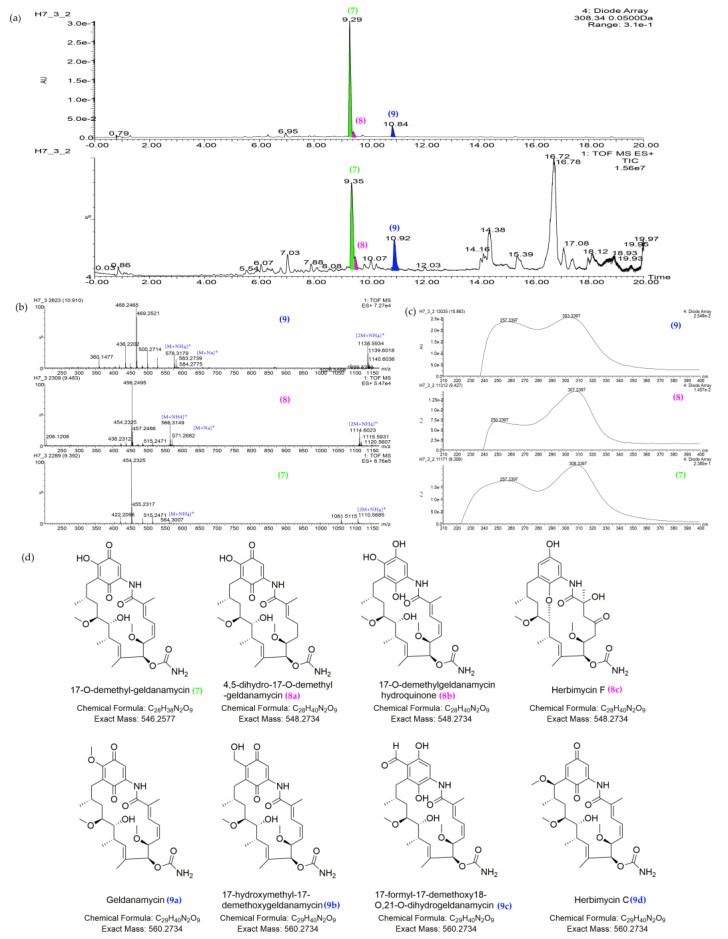
The characteristic of major benzoquinoid ansamycin-type compounds in the UPLC-UV-HRMS chromatogram of sample *Streptomyces* sp. H7 (**7**: 9.35_546.2571n, 17-*O*-demethyl-geldanamycin; **8**: 9.46_548.2717n, 4,5-dihydro-17-O-demethyl-geldanamycin (**8a**) or 17-O-demethylgeldanamycin hydroquinone (**8b**), or herbimycin F (**8c**); **9**: 10.91_560.2719n, geldanamycin (**9a**) or 17-hydroxymethyl-17-demethoxygeldanamycin (**9b**), or 17-formyl-17-demethoxy18-O,21-O-dihydrogeldanamycin (**9c**)). (**a**) UV spectra at 308 nm and TIC plot. (**b**) MS spectra of three compounds, **7**–**9**. (**c**) UV spectra of three compounds, **7**–**9**. (**d**) The structures of putative metabolites for compounds **7**–**9** after comparison with the databases and literatures.

After rapid structure dereplication using high-resolution MS data of the feature 9.35_453.2153n (**5**), it was putatively assigned to diaporisoindole D, E, aniduquinolone B, NPA001400, or NPA020460, all described as fungal metabolites by the NPAtlas database (Table 2 and Figure 3c). However, none of them matched the UV spectrum of this feature [43,44,45]. When searching its UV maximum (308 nm) in the UPLC-UV-HRMS chromatogram of sample *Streptomyces* sp. H7, two analogs of compound **5**, 9.53_456.4081*m/z* and 10.90_468.4110*m/z*, were found and they did not get any hits from the databases. The dereplication results of three homologs (9.35_453.2153n (**5**), 9.53_456.4081*m/z*, and 10.90_468.4110*m/z*) indicated they might be the putative new metabolites. However, when we reacquired the MS/MS data in the data-dependent acquisition (DDA) method, all of them showed poor MS/MS fragmentations and high background noise (Figure S4), indicating that they probably were in-source fragments (false positives) rather than molecular ion peaks when acquiring in MSE method. As a data-independent acquisition (DIA) method, the MSE can simultaneously record exact mass precursor and fragment ion information in the full *m/z* range, but precursor and fragment spectra are aligned mainly according to retention times. Therefore, the MSE acquisition might mismatch the product ions with its parent ion in the analysis of complex samples [46,47,48], leading to the misidentification of compounds when processing the data in software, such as Progenesis QI. By examination of the raw MS and MS/MS data in almost the same elution time as three in-source fragments (9.35_453.2153n (**5**), 9.53_456.4081*m/z* and 10.90_468.4110*m/z*), three other compounds, 9.35_546.2571n (**7**), 9.46_548.2717n (**8**), and 10.91_560.2719n (**9**), had a high intensity of MS/MS fragments in the DDA acquisition, and their pseudomolecular ion peaks were also identified in MS spectra of UPLC-HRMS and HPLC-MS chromatograms (Figure 4, Figures S5 and S6). Therefore, the corresponding compounds in the same elution time should be revised as compounds **7**–**9**, respectively. By searching databases and comparing their UV spectral data (Figure 4 and Figure S7) with the literature data, compounds **7**–**9** were most likely to be benzoquinoid ansamycin-type compounds as shown in Figure 4 [49,50,51]. In summary, four predominant outliers, *Streptomyces* sp. H7, *Streptomyces* sp. Y2, *Streptomyces* sp. H12, and *Streptomyces* sp. Y46, were preliminarily excluded for prospecting new antibiotics because differing features of those strains could be matched well with known antibiotics.

**Figure 5:**
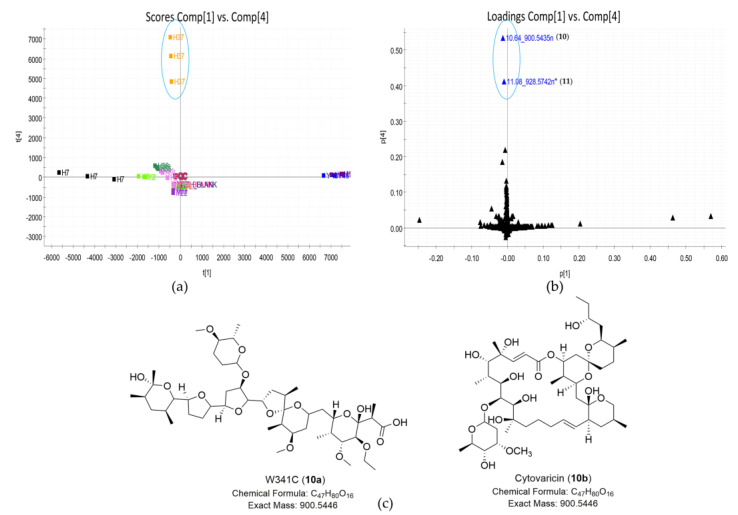
Discovery of natural products unique to strain *Streptomyces* sp. H37. (**a**) Scores plot (PC1 vs. PC4) of 23 extracts; the PC planes were adjusted to separate *Streptomyces* sp. H37; (**b**) loadings plot (PC1 vs. PC4) of 23 extracts; the loadings plot showed compounds **10**–**11** that were unique to *Streptomyces* sp. H37; *, no hit. (**c**) The structures of putative metabolites for compound **10** from *Streptomyces* sp. H37 by database dereplication.

Identifying the compounds responsible for groups near the center of the scores plot, such as sample *Streptomyces* sp. H37 or sample *Streptomyces* sp. M22 in the PC1 vs. PC2 scores plot (Figure 3a) was usually not straightforward. However, these groups could be separated by observing different PC planes [21]. This approach could lead to identifying more significant compounds that we might be interested in. As shown in Figure 5a, sample *Streptomyces* sp. H37 was separated in the PC1 vs. PC4 scores plot. Compounds unique to this sample were identified in the corresponding position in the loadings plot. Therefore, the major compounds unique to *Streptomyces* sp. H37 were shown as 10.64_900.5435n (**10**) and 11.08_928.5742n (**11**) (Figure 5b). The outlying feature (compound **10**) with neutral mass ion peak at 900.5435 Da in the predicted molecular formula C_47_H_80_O_16_ was putatively identified as either cytovaricin or W341C (Figure 5c, Table 3). The MS/MS fragment spectrum showed a series of peaks with loss of H_2_O (Figure S8), and a characteristic fragmentation pattern of polyether ionophores [39] representing compound **10** was likely identified as the polyether ionophore compound W341C. Database searches revealed no hit for feature 11.08_928.5742n (**11**), suggesting that it might be a putative new compound. Two features (**10**–**11**) showed similarity in their MS/MS spectra, demonstrating they should be the structural analogs (Figure S8).

**Figure 6:**
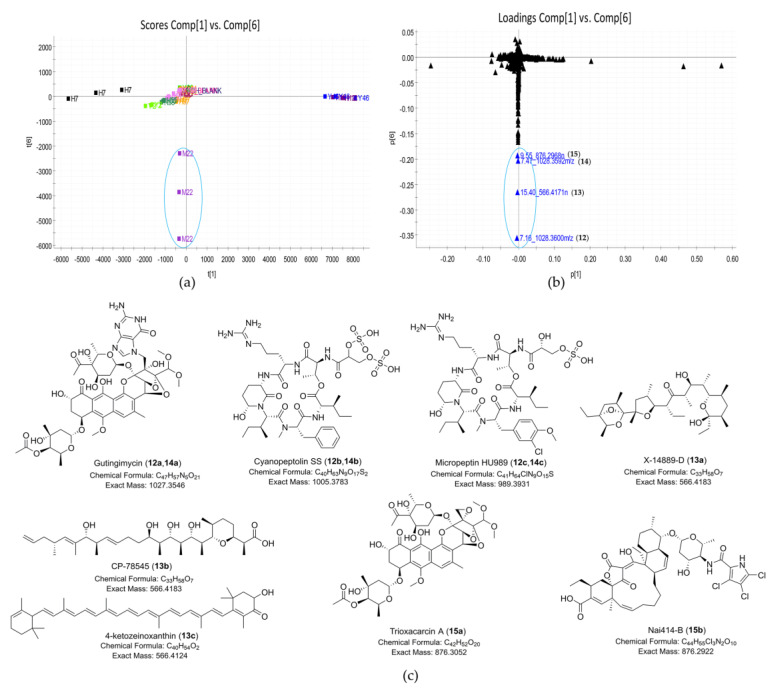
Discovery of natural products unique to strain *Streptomyces* sp. M22. (**a**) Scores plot (PC1 vs. PC6) of 23 extracts; the PC planes were adjusted to separate *Streptomyces* sp. M22; (**b**) loadings plot (PC1 vs. PC6) of 23 extracts; the loadings plot showed compounds **12**–**15** that were unique to *Streptomyces* sp. M22. (**c**) The structures of putative metabolites for compounds **12**–**15** from *Streptomyces* sp. M22 by database dereplication.

**Figure 7:**
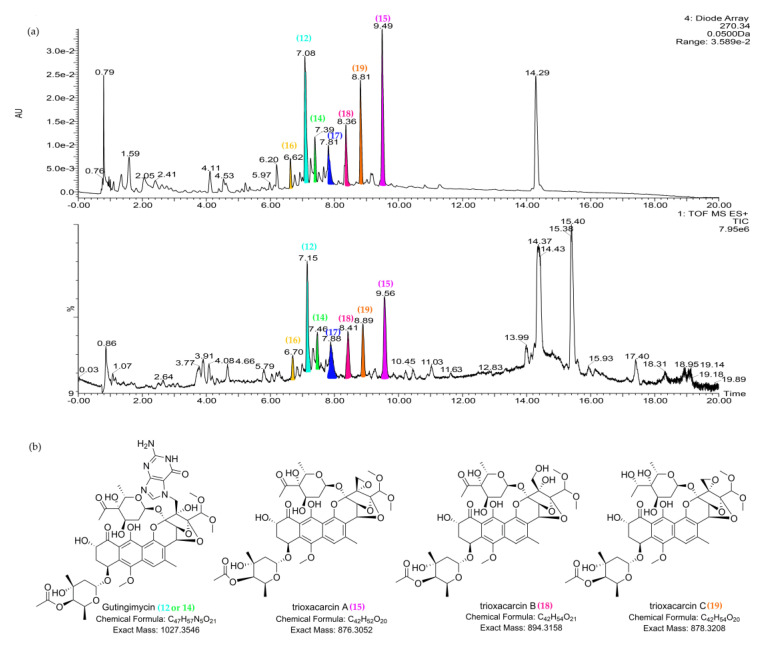
The characteristic of major trioxacarcin-type compounds in the UPLC-UV-HRMS chromatogram of *Streptomyces* sp. M22. (**12**, 7.16_1028.3600*m/z*; **14**, 7.47_1028.3592*m/z*; **15**, 9.55_876.2958n, trioxacarcin A; **16**, 6.69_1030.3751*m/z*; **17**, 7.94_1013.3486*m/z*; **18**, 8.43_894.3132n, trioxacarcin B; **19**, 8.89_878.3168n, trioxacarcin C). (**a**) UV spectra at 270 nm and TIC plot. (**b**) The structures of putative metabolites for compounds **12**, **14**–**15**, and **18**–**19** after comparison with the databases and literature.

In the scores plot (PC1 vs. PC6) (Figure 6a), strain *Streptomyces* sp. M22 was clearly distinct from other strains. The major compounds unique to *Streptomyces* sp. M22 were 7.16_1028.3600*m/z* (**12**), 15.40_566.4171n (**13**), 7.47_1028.3592*m/z* (**14**), and 9.55_876.2968n (**15**). The dereplicated results of these outlying metabolites are shown in Table 3. The UV spectra of compounds **12**, **14**, and **15** confirmed their identities as trioxacarcin-type compounds (Figure S9), corresponding to putative gutingimycin, gutingimycin, and trioxacarcin A, respectively [52,53]. Both compounds **12** and **14** matched gutingimycin in the database searching because they had the same MS data (1028.3592*m/z*), indicating that one of them should be an isomer of gutingimycin. To the best of our knowledge, no isomers of gutingimycin have been found so far (Table S5). The presence of gutingimycin isomer in 7.16 or 7.47 min (compound **12** or **14**) indicated that one of them should be a putative new compound. After examining the UPLC-UV-HRMS profile of *Streptomyces* sp. M22, trioxacarcin-type compounds, including compounds **12**, **14**, and **15,** were presented within a retention time range of 6.5–10.0 min (Figure 7, Figures S9 and S10). Two compounds, 6.69_1030.3751*m/z* (**16**) and 7.94_1013.3486*m/z* (**17**), in the chromatogram were identified as the putative new compounds after searching the databases and reverting back to the literature (Table S5). Lastly, another differing compound (**13**) was tentatively confirmed not to be 4-ketozeinoxanthin due to its lack of characteristic maximum UV absorption at 445–470 nm [54].

**Figure 8:**
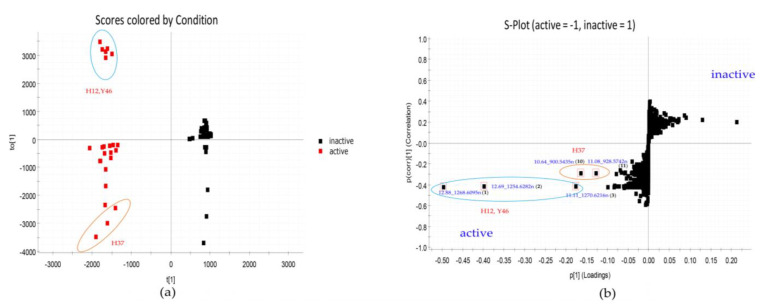
OPLS-DA analysis of 23 extracts. (**a**) OPLS-DA scores plot against *A. baumannii*. (**b**) The OPLS-DA loadings S-plot with the selected markers (12.88_1268.6095n (**1**), 12.69_1254.6282n (**2**), 11.11_1270.6216n (**3**), 10.64_900.5435n (**10**), and 11.08_928.5742n (**11**)).

As shown in Table 1, among 23 strains showing anti-MRSA activity, 8 strains also displayed inhibition zones against drug-sensitive *Acinetobacter baumannii*. However, in PCA model, the groups of active or inactive strains against the *Acinetobacter baumannii* were not completely separated from each other as shown in Figure 3a. To discriminate the two classes (active vs. inactive) and identify compounds mainly contributing to the bioactivity of the eight strains, the OPLS-DA model was constructed. As shown in Figure 8a, active and inactive samples were clearly separated in the model. The R^2^ value of 0.98 and Q^2^ value of 0.95 suggested that the OPLS-DA model possessed reliable fitness and predictability. In the S-plot analysis, five potential marker compounds were selected to chemically distinguish the active from inactive extracts (Figure 8b). The variable importance in the projection (VIP) plot showed that all selected potential markers had high VIP values (VIP ≥ 10) (Figure S11), revealing that these marker compounds were largely responsible for the discrimination between active and inactive groups. Therefore, the main contributors to the activity were the putative three actinomycins (**1**–**3**) from the group of *Streptomyces* sp. H12 and *Streptomyces* sp. Y46, and the two compounds 10.64_900.5435n (**10**) and 11.08_928.5742n (**11**) from *Streptomyces* sp. H37. To the best of our knowledge, the inhibitory activity of actinomycin D against *A. baumannii* was reported [55]. Furthermore, cytovaricin and W341C, the dereplicated metabolites for compound **10**, had no antibacterial activity against Gram-negative pathogens [56,57]. In contrast to their antibacterial spectra, compound **10** showed antibacterial activity against Gram-negative pathogens, suggesting that it might be a putative new compound.

### 2.4. Molecular Network Analysis of Outlier Strains

**Figure 9:**
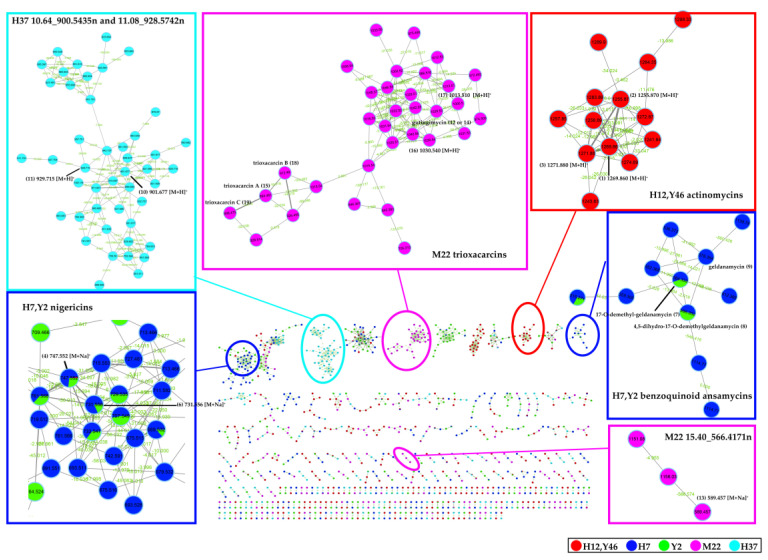
Molecular network of six outliers. The different colors of the nodes represented by different outliers, but the red node represented both *Streptomyces* sp. H12 and *Streptomyces* sp. Y46. Only clusters containing at least two nodes were shown.

**Figure 10:**
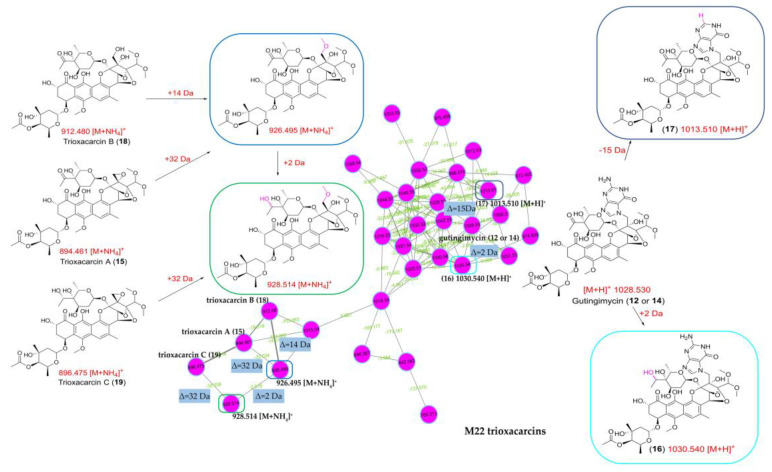
Molecular network analysis of putative trioxacarcins family in *Streptomyces* sp. M22 extract.

To survey the global map of the metabolites of six outliers (*Streptomyces* sp. Y46, *Streptomyces* sp. H12, *Streptomyces* sp. H7, *Streptomyces* sp. Y2, *Streptomyces* sp. H37, and *Streptomyces* sp. M22), classical molecular networking acquired by the DDA method was performed. After the removal of nodes associated with the blank medium control, the molecular network consisted of 5033 nodes connected with 5247 edges. It was noted that the number of nodes in the network did not correspond exactly to the number of metabolites, as different adducts or charges of the same compounds could generate different nodes [58]. As shown in Figure 9, six molecular families that contained spectra matching the discriminatory metabolites in the six outliers were identified. The putative actinomycin-type compounds, the trioxacarcins group, and benzoquinoid ansamycin-type compounds only existed in samples of *Streptomyces* sp. H12 and *Streptomyces* sp. Y46, *Streptomyces* sp. M22, *Streptomyces* sp. H7 and *Streptomyces* sp. Y2, respectively. They were in agreement with the results of the PCA analysis. In addition, the molecular network could clarify the relationship of the discriminatory metabolites in the outlier strains, assisting in the identification of the discriminatory metabolites. The two discriminatory metabolites 16.71_724.4749n (**4**) and 18.21_708.4796n (**6**) clustered as adjacent nodes in the nigericin family (Figure 9), indicating that compound **6** could be assigned as putative grisorixin or epigrisorixin, the analog of compound **4**. The putative metabolites (**8a** and **9a**) showed higher structural similarity with 17-*O*-demethyl-geldanamycin (**7**) than other “hit” compounds (**8b**–**8c**; **9b**–**9d**), and thus, compounds **8** and **9** in *Streptomyces* sp. H7 and *Streptomyces* sp. Y2 were further putatively deduced as 4,5-dihydro-17-*O*-demethyl-geldanamycin and geldanamycin, respectively (Figure 9). Similarly, the compounds 10.64_900.5435n (**10**) and 11.08_928.5742n (**11**) from *Streptomyces* sp. H37 were further confirmed as two congeners with a mass difference of 28 Da (CO or C_2_H_4_ group). In sample *Streptomyces* sp. M22, two putative new compounds, 1030.3751*m/z* (**16**) and 1013.3486*m/z* (**17**), were adjacent to gutingimycin (**12** or **14**) with mass differences of 2 and 15 Da, respectively, suggesting that the former was the putative hydrogenated gutingimycin, and the latter was the putative gutingimycin with loss of NH group (Figure 10).

Furthermore, a large number of nodes clustered with the discriminatory metabolites suggested the presence of additional analogs in these compound classes. For example, in addition to trioxacarcin A (**15**), B (**18**), and C (**19**), the inspection of their parent mass, mass difference, and the MS/MS data implied that putative methylated trioxacarcin B (*m/z* 926.495) and its hydrogenation product (*m/z* 928.514) were present (Figure 10). Another outlier, 15.40_566.4171n (**13**), in the *Streptomyces* sp. M22 was clustered with two other compounds, whose molecular weights were almost twice as high, indicating that its dimers might exist in the extract (Figure S12). In the cluster containing the 10.64_900.5435n (**10**) and 11.08_928.5742n (**11**) of *Streptomyces* sp. H37, putative dehydrogenated, methylated, and demethylated analogs were detected (Figure S13). However, most of the putative new compounds discovered from the molecular network were presented as trace components in the extracts when checking their original UPLC-HRMS/MS data.

**Figure 11:**
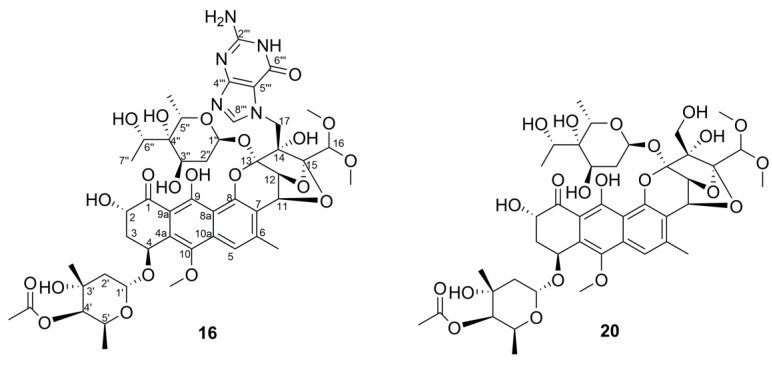
Chemical structures of gutingimycin B (**16**) and trioxacarcin G (**20**).

According to the results above, actinomycin-producing strains *Streptomyces* sp. Y46 and *Streptomyces* sp. H12, and nigericin-producing strains *Streptomyces* sp. H7 and *Streptomyces* sp. Y2 were excluded since their discriminatory metabolites were well known and frequently discovered from the actinomycetial strains, even though they were chemically unique. Samples of *Streptomyces* sp. H37 and *Streptomyces* sp. M22 were prioritized for scale-up and further isolation work. The strain *Streptomyces* sp. H37 was selected because the PCA analysis revealed that it contained the putative novel compounds. Further analysis using the OPLS-DA model identified these putative novel compounds contributing to significant inhibitory activity against drug-sensitive *A. baumannii*. Lastly, a variety of their potential new analogs were found through molecular network analysis. The purification and structural elucidation of new putative compounds in *Streptomyces* sp. H37 is still underway. The strain *Streptomyces* sp. M22 was selected because the putative novel trioxacarcin-type compounds were identified by the metabolomics-based dereplication approach (PCA, metabolic profile, molecular network, and database searching). Furthermore, the structures of some putative novel compounds were deduced from molecular networking. As a result, two novel trioxacarcin analogs, gutingimycin B (**16**) and trioxacarcin G (**20**) (Figure 11), along with gutingimycin (**12**) from the strain *Streptomyces* sp. M22, were isolated and structurally identified after large-scale fermentation.

### 2.5. Structure Elucidation of Trioxacarcin Compounds from Strain Streptomyces sp. M22

Gutingimycin B (**16**) was obtained as a yellow powder. It fluoresced green in solution upon irradiation with 365 nm light and showed the UV absorption maxima at 274 nm and 408 nm (Figure S14), typical for trioxacarcin-type compounds. Its molecular formula was assigned as C_47_H_59_N_5_O_21_ on the basis of HRESIMS peak at *m/z* 1030.3778 [M+H]^+^ (calcd for C_47_H_60_N_5_O_21_, 1030.3781) (Figure S15), 2 mass units more than gutingimycin. The ^1^H NMR spectra (Table 4 and Figure S17) showed characteristic resonances for an exchangeable OH proton (*δ*_H_ 13.84 (s)), two aromatic protons (*δ*_H_ 8.22 (s) and 7.48 (s)), two anomeric protons (*δ*_H_ 5.64 (d, 3.6) and 5.51 (d, 4.2)), a pair of coupling oxygenated methines (*δ*_H_ 5.22 (d, 4.2) and 5.10 (d, 4.2)), a pair of geminal coupling methylenes (*δ*_H_ 5.02 (d, 15.6) and 4.33 (d,15.6)), three methoxyl singlets (*δ*_H_ 3.95 (s), 3.66 (s) and 3.53 (s)), two methyls attached to olefinic or carbonyl carbon (*δ*_H_ 2.62 (s) and 2.25 (s)), three methyl doublets (*δ*_H_ 1.37 (d, 6.6), 1.26 (d, 6.6) and 1.26 (d, 6.6)), and one methyl singlet (*δ*_H_ 1.18 (s)). In accordance with the molecular formula, the ^13^C (Table 4 and Figure S18) and DEPT NMR spectra (Figure S19) showed the 47 carbon resonances assigned to 1 keto carbonyl (*δ*_C_ 207.6), 1 ester carbonyl (*δ*_C_ 173.3), a characteristic of the guanine group (*δ*_C_ 157.8, 153.8, 151.8, 140.6, and 108.1), 3 naphthalene carbons bearing to oxygen (*δ*_C_ 162.9, 152.7, and 144.6), 6 acetal carbons or electron-rich *sp2* atoms (*δ*_C_ 108.8, 108.3, 103.3, 100.9, 99.6, and 92.5), 15 oxygenated methines (*δ*_C_ 56.1–84.4), 4 methylenes (*δ*_C_ 46.2, 37.7, 37.1, and 33.8), and 6 methyls (*δ*_C_ 15.7–27.3). The aforementioned NMR data of compound **16** showed very close similarities with those of gutingimycin [52]. The differences were an additional methine quartet at *δ*_H_ 3.99 (q, 6.6) and a methyl doublet at *δ*_H_ 1.37 (d, 6.6) in compound **16** instead of the methyl singlet at *δ*_H_ 2.50 (s) in gutingimycin. In the ^13^C NMR spectra, the carbonyl at *δ*_C_ 211.1 in gutingimycin was missing, and additional oxygenated methine at *δ*_C_ 70.3 in compound **16** was detected. The missing ketone signal, together with the additional two mass units compared to gutingimycin, indicated that the ketone group in the sugar moiety of gutingimycin was reduced to an alcohol in compound **16**.

**Figure 12:**
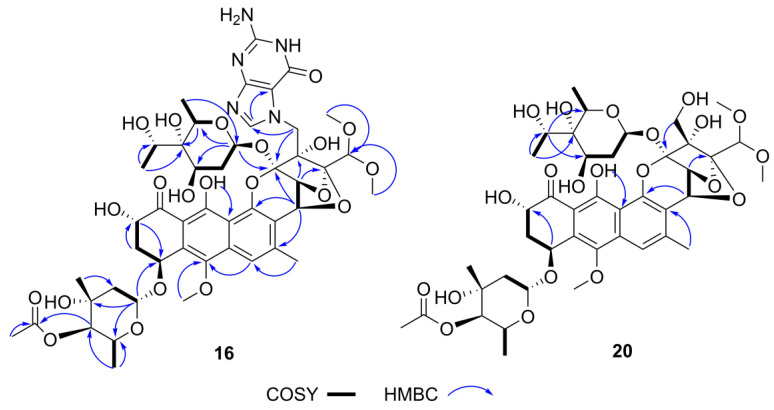
The key ^1^H-^1^H COSY and HMBC correlations of **16** and **20**.

The abovementioned structural features in compound **16** were confirmed by the 2D NMR spectra (Figures S20–S22). The crucial ^1^H-^1^H COSY correlation of H-6″ (δ_H_ 3.99, q, 6.6)/H_3_-7″ (*δ*_H_ 1.37, d, 6.6) and the key HMBC correlation from H_3_-7″ to C-6″ (*δ*_C_ 70.3), C-4″ (72.1) (Figure 12) indicated that the hydroxyl group was linked to the C-6″ position as the keto-reduced trioxacarcinose B.

Trioxacarcin G (**20**) was obtained as a yellow powder. The UV absorption maxima at 232, 271, and 400 nm (Figure S23) and fluorescence under 365 nm light indicated that compound **20** is a trioxacarcin-type compound. The HRESIMS suggested that its molecular formula was determined to be C_42_H_56_O_21_ (*m/z* 914.3644 [M+NH_4_]^+^, calcd C_42_H_60_NO_21_, 914.3658) (Figure S24), 2 mass units more than trioxacarcin B. ^1^H and ^13^C NMR of compound **20** (Table 4, Figures S26–S28) were nearly identical to those of trioxacarcin B [59,60], except for an additional methine quartet at *δ*_H_/*δ*_C_ 3.91 (q, 6.6)/70.7 and a methyl doublet at *δ*_H_/*δ*_C_ 1.34 (d, 6.6)/18.2 in replacement of the carbonyl at *δ*_C_ 210.9 and the methyl singlet at *δ*_H_ /*δ*_C_ 2.46 (s)/28.1. These were similar to the difference between compound **16** and gutingimycin, indicating that the ketone group in the L-trioxacarcinoses B of trioxacarcin B was also reduced to an alcohol in the keto-reduced trioxacarcinose B of compound **20**. The location of hydroxyl group was assigned at C-6″ based on the ^1^H-^1^H COSY correlation of H-6″ (*δ*_H_ 3.91, q, 6.6)/H_3_-7″ (*δ*_H_ 1.34, d, 6.6) and the HMBC correlations from H_3_-7″ to C-6″ (*δ*_C_ 70.7), C-4″ (*δ*_C_ 72.6) and from H-6″ to C-3″ (*δ*_C_ 68.4), C-5″ (*δ*_C_ 66.3) (Figure 12 and Figures S29–S31).

The NMR data of the keto-reduced trioxacarcinose B in **16** and **20** were closely similar with those of trioxacarcin C rather than epi-6″-trioxacarcin C, indicating that the absolute configuration at C-6″of **16** and **20** was determined as 6″S, the same as trioxacarcin C [61]. According to the literature [60], the X-ray structure of gutingimycin delivered the stereochemistry of the trioxacarcin skeleton, and the sugar moieties of the trioxacarcins A–B were identified previously as L-trioxacarcinoses A and B. In addition, compounds **16** and **20** had the same specific rotation sign as the known trioxacarcin, also isolated in the present study, gutingimycin (**12**) (${\left[\alpha \right]}_{D}^{25}$ −60.0° (c 0.02, ACN)). Therefore, the absolute configuration of compounds **16** and **20** was established, as shown in Figure 11. In addition, the structures of known compounds were identified as gutingimycin (**12**) by comparison of their physio-chemical and spectroscopic data (Figures S32–S37) with those of the literature [52].

## 3. Discussion

Actinomycetia derived from mangroves is a promising source for exploring novel bioactive natural products. A multitude of bioactive compounds, including the promising compounds salinosporamide A, xiamycins, and indolocarbazoles, have been isolated from mangrove actinomycetia [13,14,62,63]. Salinosporamide A, a potent 20S proteasome inhibitor, is the first mangrove-derived compound that entered phase I clinical trials for the treatment of multiple myeloma only three years after its discovery [13,64,65]. Xiamycin exhibits selective anti-HIV activity and also represents one of the first examples of indolosesquiterpenes isolated from prokaryotes [66]. The Leizhou Peninsula, located in the southernmost end of mainland China, has 9284.3 ha of mangrove distributed in over 100 sites along the coastlines, comprising approximately 79% of the total mangroves area in Guangdong province and 33% in China [67,68]. However, the mangrove from the Leizhou Peninsula is underexplored, and only a few reports on the diversity and antibacterial activity of its inhabiting actinomycetia have been published to date. Our group started exploring the diversity and bioactivity of the mangrove plant endophytic actinomycetia in this region in 2015. In total, 159 strains in 19 genera affiliated with 12 families were isolated, and 64 out of 88 tested strains exhibited activity against at least one of the tested pathogens [18]. In order to maximize the harvest of actinomycetial strains in this study, 13 soil samples from different mangrove sites were collected, and 12 isolation media were applied, leading to the isolation of 521 strains in 40 genera from 23 families. Thus, this ecosystem is proven to provide a highly productive and rich diversity of actinomycetia. The genera *Micromonospora* and *Streptomyces* were still numerically dominant, which is consistent with previous studies [63]. Interestingly, the addition of kelp or coconut juice into the medium (M11 and M12) increased the diversity of actinomycetia compared with the blank control (M1 and M4), which might be caused by trace substances from kelp or coconut juice assisting some strains growth since both seaweed and coconut trees grow in or along the sea and live in similar environments as mangroves. Lastly, 179 strains affiliated to 40 different genera with a unique colony morphology were selected to evaluate their antibacterial activities.

Bioactivity screening is, in general, the initial step to find novel antibiotics. To comprehensively analyze the antibacterial spectra of the extracts, 12 strains of drug-sensitive and drug-resistant pathogens were used as indicators in this study. In order to find the strains producing more potent antibacterial metabolites, the test volume of extracts used in the assay was reduced to 50% of the normally applied volume in the same concentration. In this assay, we found that *Streptomyces* was still the main genus producing bioactive secondary metabolites, accounting for 61.7% of all active strains. Because the ISP2 medium supports the growth of most actinomycetia and also is known to promote secondary metabolite production [24,69,70], it was the only medium used for fermentation. ISP2 medium components are also reported to reduce noise level and interference with secondary metabolites detection in the UPLC–HRMS analysis [24]. In addition, application of only one medium facilitates comparability between the different strains in metabolomics analysis [71].

Dereplication has become a key issue for the discovery of new antibiotics. An effective approach was required to maximize the detection of chemical diversity and minimize the redundancy of the samples after the bioactivity and phylogeny screening [72,73]. UPLC-HRMS/MS-based metabolomics could maximize the detection of chemical diversity among extracts in a high throughput manner [74]. UPLC-HRMS/MS gives rapid separation from the complex strain extracts and increases confidence in identifying metabolites based on mass accuracy and isotope fit. Metabolomic methods are combined with chemoinformatics approaches, such as PCA, OPLS-DA, etc. PCA, an unsupervised method without grouping, was applied to overview the differences among numerous samples, identifying strains with distinct metabolites, while excluding strains with common chemical profiles [75]. Hou Yangpeng et al. applied LC/MS-PCA to discover novel natural products from marine-derived *Streptomyces* spp.; the result was that 37% of all isolates produced a number of unique and putative new natural products, indicating that this approach could greatly improve the discovery rate from *Streptomyces* spp. [21]. However, the application of PCA was limited to the number of samples, usually between 20 and 50 strains, practically [21,24]. Therefore, 23 strains with strong anti-MRSA activity were selected for PCA analysis. The result showed that six strains with unique chemical profiles could be candidates for prioritization. After dereplicating, two *Streptomyces* strains (M22, H37) with putative novel compounds were prioritized.

OPLS-DA, a supervised method, was an effective statistic model for comparing two different sample groups [20]. A total of 8 out of the above 23 strains showing bioactivity against drug-sensitive *A. baumannii* were prioritized using the OPLS-DA model to find the bioactive compounds produced in the strains. Finally, *Streptomyces* sp. H37 with putative new compounds responsible for inhibitory activity against *A. baumannii* were prioritized. The OPLS-DA analysis effectively assists the bioactivity screening to find potential new antibiotics with great bioactivity and avoid targeting inactive metabolites in the follow-up chemical isolation. Molecular networking was a new dereplication strategy to rapidly overview the chemical family in the extracts, identify known compounds, and find the analogs with novelty. In this study, it can act as a complement for the PCA and OPLS-DA analysis to claim the relationship of the significant metabolites in the outlier strains, find the potential new congeners, and predict the structure using their MS/MS similarity. As shown in Figure 10, the predicted structure of compound **16** was consistent with the structure that was identified by spectroscopic analysis, including HRESIMS, 1D, and 2D NMR.

NPAtlas and StreptomeDB are recommended as the databases used for the dereplication of metabolites produced in microbial extracts, especially in actinomycetial extracts. These databases are manually curated by the Linington group out of Simon Fraser and Stefan Günther group from Freiburg, respectively. Unlike Antibase, MarinLit, and Dictionary of Natural Products (DNP), NPAtlas and StreptomeDB are open access, updated, and ready to be downloaded. To date, NPAtlas v19_12 contains over 25,000 microbial-produced natural products [33], and StreptomeDB v3.0 includes 6524 compounds produced by *Streptomyces* [34]. As shown in Table 2 and Table 3, the dereplication of metabolites from different databases produced different results sometimes. If two professional microbial databases are simultaneously applied to the dereplication process, it can increase accuracy and effectiveness in dereplication and avoid omissions caused by using one database. Hence, more than one database should be used, and loose parameters, such as precursor and fragments tolerances in 10 ppm, should be set in the search method. The limitation of dereplication for secondary metabolites is usually caused by the difficulty of obtaining an authentic standard for every “hit” from the database. Additional data, such as UV and MS/MS data, can be helpful to ensure that the identified hits in the mass ion peaks are correct.

The trioxacarcin family is a family of complex aromatic polyketides, which is produced by *Streptomyces* strains. In 1981, the trioxacarcins (trioxacarcins A–C) were first isolated from *Streptomyces bottropensis* DO-45 [76]. Subsequently, they were reisolated from a marine *Streptomyces* sp. B8652 with additional four new analogs, trioxacarcin D–F and gutingimycin, in 2004 [52,60]. The structure of the trioxacarcins was characterized as a rigid, highly oxygenated polycyclic skeleton with a fused spiro epoxide function, and one or more unusual glycosidic residues, named ‘trioxacarcinoses’ [77]. Trioxacarcin-type compounds display extraordinary antiproliferative effects, such as anticancer, antibacterial, and anti-malaria activities [60,76,78], which have attracted attention for chemical synthesis [61,77,79,80], mode of action, and biosynthesis studies [81,82,83,84,85]. It was reported that the notable biological activity of trioxacarcin A is due to its tight interaction with DNA [86,87]. In our study, several putative novel trioxacarcin-type compounds were identified by the metabolomics-based dereplication approach, such as compound **12** (7.16_1028.3600*m/z*), compound **14** (7.47_1028.3592*m/z*), compound **16** (6.69_1030.3751*m/z*), compound **17** (7.94_1013.3486*m/z*), and so on. However, some of them were unstable in scale-up fermentation. The contents of compounds **14** and **17** were decreased, making them hard to be isolated and accumulated for structural identification. Meanwhile, a low-yield compound **20** (7.86_896.3265n) was increased in the scale-up fermentation. A similar phenomenon was also reported by other researchers in metabolomics analysis of bacterial strains [88]. Finally, two new trioxacarcins, gutingimycin B (**16**) and trioxacarcin G (**20**), along with gutingimycin (**12**, 7.16_1028.3600*m/z*) were isolated from the scale-up fermentation broth of *Streptomyces* sp. M22. To the best of our knowledge, it is the second report of finding trioxacarcins with keto-reduced trioxacarcinose B moiety, except for trioxacarcin C. The new trioxacarcin-type members, compound **16** and compound **20**, together with known compound **12**, were evaluated for cytotoxicity against the H460 lung cancer cell line in this study, but no prominent cytotoxic activity against this cell line was observed in these compounds (IC_50_ > 1000 nM). The antibacterial activity of the new analogs and their cytotoxicity against other cell lines will be tested in the future. The successful isolation of novel trioxacarcin-type compounds from *Streptomyces* sp. M22 has demonstrated that our integrative strategies are effective, efficient, and suitable for seeking new antibiotics from those ecosystems inhabiting a large amount of actinomycetial strains.

## 4. Materials and Methods

### 4.1. Samples Collection

**Figure 13:**
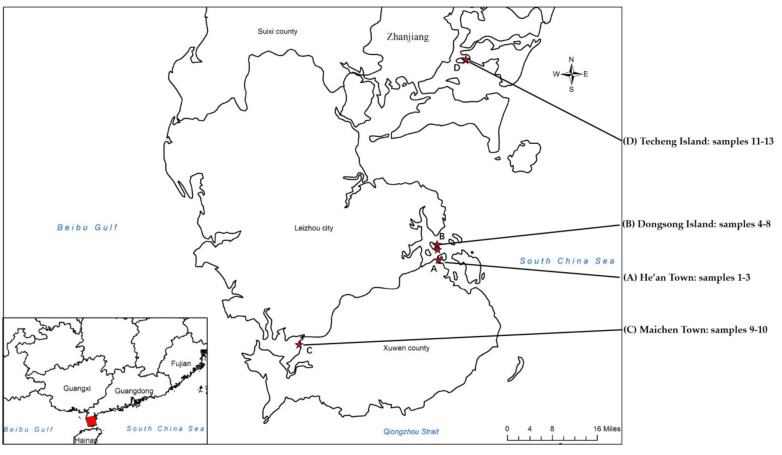
Locations of the sampling sites (red star) in Leizhou Peninsula, Guangdong, China.

Soil samples were collected in August 2019 at different mangrove sites in Leizhou Peninsular, Guangdong province, China. The locations where samples were collected and their information are shown in Figure 13 and Table S6. All samples were collected from depth of 5–10 cm with a sterile spatula, then packed in sterile bags, and brought to the laboratory at the earliest time. Each sample was air-dried in a laminar flow hood before grinding with a mortar and pestle.

### 4.2. Isolation of Actinomycetial Strains

A total of 12 cultural media were used to isolate actinomycetial strains. All media were supplemented with nalidixic acid (25 mg/L), cycloheximide (50 mg/L), and potassium dichromate (50 mg/L) to inhibit the growth of Gram-negative bacteria and fungi. The recipes for 12 media are shown in Table S1. It should be mentioned that M11 is a modified version of M1 with the addition of 15 mL kelp solution instead of 15 mL distilled water in the recipe. The kelp solution was prepared as follows: 200 g fresh kelp was cut into small pieces, added into 200 mL distilled water, and boiled for 30 min. After cooling down, the kelp soup was filtered by absorbent cotton to obtain the kelp solution. Meanwhile, M12 is a modified version of M4 with the addition of 10 mL natural fresh coconut juice instead of 10 mL distilled water in the recipe.

Strains were isolated by using the dilution plating technique. A total of 5 g of each soil sample was diluted with 45 mL of sterile 0.1% Na_4_P_2_O_7_ solution, then mixed, homogenized, and shaken for 1 h at 180 rpm to release actinomycetia cells attached to the soil. Subsequently, the pretreated samples were prepared for ten-fold serial dilutions up to 10^−4^. A total of 0.2 mL of diluted sample (10^−2^, 10^−3^, and 10^−4^) from each soil sample was spread onto isolation agar plates, and plates were incubated for 7–14 days at 28 °C. Actinomycetia-like colonies depending on their morphological characters, pigment diffusion, and coloration of their mycelia were picked and streaked several times on ISP2 agar plates until pure actinomycetial colonies were isolated. The pure strains were maintained on ISP2 agar slants at 4 °C and preserved in 20% glycerol (*v*/*v*) at −80 °C.

### 4.3. Phylogenetic Analysis

Genomic DNA was extracted by using a rapid method with Chelex-100 as described previously [89]. The 16S rRNA gene amplification and sequencing analysis were performed using the universal primers 27F (5′-AGAGTTTGATCMTGGCTCAG-3′) and 1492R (5′-GGTTACCTTGTTACGACTT-3′). The PCR reaction mixture (50 μL) included 25 μL 2× supermix (TransGen Biotech, Beijing, China), 1 μL each of the primers (10 mM, Sangon Biotech, Shanghai, China), 1.5 μL DNA, and 21.5 μL ddH_2_O. The reaction conditions were as follows: 95 °C for 3 min, 30 cycles of 94 °C for 1 min, annealing at 60 °C for 1 min, extension at 72 °C for 1 min, followed by final extension for 10 min at 72 °C. The amplified products were sent to Shanghai Shenggong Company for sequencing. The sequencing data were BLAST analyzed using the GenBank NCBI (http://www.ncbi.nlm.nih.gov/, accessed on 11 November 2021) and the EzBioCloud database [90] to determine the similarity with type strains. Multiple alignments were generated using the Clustal_X tool in MEGA version 7.0 [91]. A phylogenetic tree based on the neighbor-joining method was constructed under Kimura’s two-parameter model [92]. Bootstrap analysis with 1000 replications was performed with MEGA version 7.0 and finally visualized via the Interactive Tree of Life (iTOL) web service [93].

### 4.4. Extracts Preparation and Bioactivity Assay

Based on the analysis of phenotypic and phylogenetic characteristics, 179 strains were selected from the 521 isolated actinomycetial strains to examine their antibacterial potentials. The strains were inoculated into 100 mL ISP2 broth in 500 mL conical flasks and cultured for 7 days in a shaking incubator at 180 rpm at 28 °C. A total of 300 mL (3 × 100 mL) cultural broth of each strain was pooled and centrifuged at 4200 rpm for 20 min to separate the mycelium portion. The supernatants were extracted three times with ethyl acetate (1:1, *v*/*v*). The organic layers were combined and evaporated to obtain crude extracts. The crude extracts were dissolved in 3 mL methanol and used for antibacterial assay by the paper disc diffusion method.

The methanol sample (30 µL) was dripped on a paper disk (6 mm diameter). A total of 30 µL methanol and levofloxacin solution (10 µL, 1 mg/mL) were used as the negative and positive control, respectively. After being dried in a biosafety hood, the paper disks were transferred to agar plates seeded with pathogenic bacteria and incubated at 37 °C for 24–48 h. The antibacterial activity was evaluated by measuring the diameters of the inhibition zones with a vernier caliper. The indicator bacteria used for antimicrobial assay were six sets of indicator bacteria, including *Enterococcus* sp. (ATCC 33186 and 310682), *Staphylococcus aureus* (ATCC 29213 and ATCC 33591), *Klebsiella pneumonia* (ATCC 10031 and ATCC 700603), *Acinetobacter baumannii* (2799 and ATCC 19606), *Pseudomonas aeruginosa* (ATCC 27853 and 2774) and *Escherichia coli* (ATCC 25922 and ATCC 35218). Their drug susceptibility testing was identified and confirmed by the Beijing Key Laboratory of Antimicrobial Agents, Institute of Medicinal Biotechnology. Each set consisted of two strains, one drug-sensitive strain (the former), and one drug-resistant strain (the latter). Isolate 310682 was resistant to vancomycin. Meanwhile, isolate 2774 was resistant to aminoglycosides and carbapenems. Indicator bacteria were obtained from either the American Type Culture Collection (ATCC) or the clinic isolation from hospital in China, and they were deposited in the Institute of Medicinal Biotechnology, Chinese Academy of Medical Sciences and Peking Union Medical College.

### 4.5. PCA and OPLS-DA Analysis

Twenty-three strains with zones of inhibition larger than or equal to 10 mm against MRSA were selected for dereplication and microbial strain prioritization studies using UPLC-HRMS-PCA and UPLC-HRMS-OPLS-DA. Three biological replicates were prepared for each actinomycetial strain. Each strain was cultured in triplicate for 7 days in ISP2 broth medium (3 × 100 mL) as mentioned above. Only 15 mL supernatant from 100 mL cultural broth were extracted three times (3 × 15 mL) with ethyl acetate, then dried under vacuum to obtain the crude extract. The dried crude extracts were weighed and dissolved in methanol to yield a stock solution with a concentration of 2 mg/mL. The ISP2 broth medium was used as a blank medium control. After centrifugation at 14,000 rpm for 10 min, the supernatant of the stock solution was diluted 4-fold with methanol to yield the test solution (0.05 mg/mL). A quality control (QC) sample was prepared by mixing an equal volume of each test solution (including bank medium control). All test solutions were stored at 4 °C before analysis.

UPLC-HRMS/MS experiments were carried out on Waters ACQUITY UPLC I-Class system combined with Waters Xevo G2-XS Q-TOF mass spectrometer (Waters, Manchester, UK). A Waters ACQUITY UPLC BEH C18 column (2.1 × 100 mm, 1.7 μm) maintained at 25 °C was used, and the PDA scan range was 200–800 nm. The binary mobile phase consisted of solvent A (water containing 0.1% formic acid) and solvent B (acetonitrile). The gradient elution program was applied as follows: 0–1 min, 10%(B); 1–18 min, 10–95%(B); 18–20 min, 95%(B); 20–22 min, 10%(B). The flow rate was 0.3 mL/min. The injection volume was 2 μL, and the QC sample was analyzed after every six injections to evaluate system stability.

The ESI source parameters in the positive mode were set as follows: capillary, 2 kV; sampling cone, 40 V; source offset, 80 V; source temperature, 100 °C; desolvation temperature, 250 °C; cone gas, 50 L/h; and desolvation gas, 600 L/h. The desolvation and cone gases were nitrogen, and the collision gas was argon. The MSE acquisition (data-independent acquisition) was obtained in the continuum format with a mass range of 100–2000 Da in both low-energy (function 1) and high-energy (function 2) scan functions. For function 1, the collision energy was 2 V. For function 2, a collision energy ramp of 40–80 V was used. The scan time was 0.10 s. The mass accuracy was maintained by using a lock spray with leucine–enkephalin ([M+H]^+^ = 556.2771 Da) at a concentration of 2 ng/mL and a flow rate of 5 μL/min as reference. The run sequence started with a blank solvent, then a blank medium, followed by the samples. The instrument controlling and data acquisition were performed by MassLynx V4.1 software (Waters, Milford, CT, USA).

The acquired raw data from 87 samples, including 69 test samples, six blank samples (blank solvent and blank medium), and 12 QC injections, were all imported into Progenesis QI 3.0 software (Waters, Milford, USA) to operate the chromatographic peak alignment, experimental design setup, peak picking, normalization, deconvolution, compound identification, and compound review. The imported 87 runs were aligned on the basis of an automatically selected QC sample. The retention time for peak picking was set as 0–20 min, and the limits and sensitivity were set as the default. The adduct ion forms of [M+H-H_2_O]^+^, [M+H]^+^, [M+NH_4_]^+^, [M+Na]^+^, [M+K]^+^, [2M+H]^+^, [2M+Na]^+^ were added to deconvolute the spectral data. After performing automatic processing to all compounds in all samples, a data matrix involving sample code, RT, *m/z*, and normalized abundance was generated. In the blank samples, features with the most abundance and an abundance 20 times less than the 69 test samples were hidden manually to remove medium and blank effects for cleaner data [20,71]. The obtained data were exported into the extended statistics module EZinfo 3.0 (Umetrics, Umea, Sweden) for PCA and OPLS-DA analyses. The significant differential retention time-observed mass (RT-*m/z*) or retention time-neutral mass (RT-*m/z*) pairs in the loadings plot and S-plot were selected and imported back into Progenesis QI for compound identification. After filtering by ANOVA *p*-value ≤ 0.05, *q* value ≤ 0.05, and max fold change ≥ 2, the filtered pairs were identified using the search method in Progenesis QI (parameters in search method: precursor tolerance 10 ppm and theoretical fragment tolerance 10 ppm). The Natural Product Atlas v19_12 and StreptomeDB v3.0 databases as in-house libraries were use for the dereplication of the differential metabolites in the samples.

### 4.6. Molecular Network Analysis

The UPLC and ESI source parameters were set as same as shown above. DDA was also performed in positive ion mode. The full MS survey scan was performed for 0.2 s in the range of 100–2000 Da, and MS/MS scanned a mass range of 50–2000 Da by the same scan time. The five most intense ions were chosen for MS/MS fragmentation spectra. The gradient of collision energy was set as 20 V to 40 V for low-mass collision energy (LM CE) and 60 V to 80 V for high-mass collision energy (HM CE). Automatic switching to MS/MS mode was enabled when the TIC intensity rose above 10,000 counts and switched off when 0.4 s had elapsed, or the TIC intensity was 1,000,000 counts. The tolerance window of ±3.0 Da was set in the deisotope peak detection mode. Dynamic peak exclusion was enabled, acquired, and then excluded for 3.0 s. Fixed peak exclusion was as follows: *m/z* 205.0877, 255.1581, 279.1591, 301.1425, 371.3183, 579.2933. Raw data files obtained from the DDA acquisition were converted to 32-bit mzxML format with MS-Convert [94] and then uploaded on the GNPS web platform (http://gnps.ucsd.edu, accessed on 11 November 2021) for dereplication and molecular networking construction.

A molecular network was created using the online workflow on the GNPS website (https://ccms-ucsd.github.io/GNPSDocumentation/, accessed on 11 November 2021). The precursor ion mass tolerance was set as 0.1 Da and an MS/MS fragment ion tolerance as 0.1 Da. A network was then created where edges were filtered to have a cosine score above 0.6 and more than 4 matched peaks. Furthermore, edges between two nodes were kept in the network only if each of the nodes appeared in each other’s respective top 10 most similar nodes. Finally, the maximum size of a molecular family was set to 100, and the lowest scoring edges were removed from molecular families until the molecular family size was below this threshold. The spectra in the network were then searched against GNPS spectral libraries. The library spectra were filtered in the same manner as the input data. All matches kept between network spectra and library spectra were required to have scores above 0.6 and at least 3 matching peaks. The generated molecular network was visualized using Cytoscape 3.7.1 [95].

### 4.7. Scale-Up Fermentation, Extraction, and Purification of Natural Products

*Streptomyces* sp. M22 was grown and maintained on an ISP2 agar plate at 28 °C for 7–10 days. The spores of the strain were inoculated into 500 mL Erlenmeyer flasks contained 100 mL of the ISP2 medium, which grew at 28 °C for 2 days at 180 rpm as seed cultures. Then, each seed culture (100 mL) was inoculated into autoclaved 5 L Erlenmeyer flasks containing 1 L ISP2 medium. The flasks were incubated at 28 °C for 7 days on a rotary shaker (180 rpm). The total 18 L (18 × 1L) of fermentation broth was centrifuged at 4300 rpm for 20 min, and the supernatant was extracted three times with ethyl acetate (18 L/time) to give an organic extract. After 3 times of fermentation, the combined organic extract (5.5 g) was subjected to MPLC column chromatography eluted with MeOH-H_2_O (10:90, 30:70, 50:50, 70:30, 90:10, 100:0, *v*/*v*) to obtain six subfractions (Fr.01–Fr.06) based on LC-MS analysis. Fraction 05 was further separated by Sephadex LH-20 (CH_2_Cl_2_: MeOH=1:1, *v*/*v*) to yield five subfractions (Fr.05a–Fr.05e). Fr.05b was subjected to semi-preparative HPLC (ACN-H_2_O, 32:68, *v*/*v*, 0–5 min; 32:68–52:48, *v*/*v*, 5–40 min, 3.0 mL/min) to yield gutingimycin B (**16**, 6.0 mg), gutingimycin (**12**, 15.6 mg) and semi-pure trioxacarcin G. The semi-pure trioxacarcin G was further fractioned by semi-preparative HPLC using MeOH-H_2_O (55:45, *v*/*v*) to yield pure trioxacarcin G (**20**, 8.0 mg).

Gutingimycin B (**16**): yellow amorphous powder. ${\left[\alpha \right]}_{D}^{25}$ −49.4° (c 0.02, ACN); UV (MeOH) λ_max_ (log ε) 274 (4.83), 408 (4.23) nm; IR *v*_max_: 3366, 2932, 2853, 1689, 1628, 1386, 1223, 1089, 999 cm^−1^; ^1^H NMR (CDCl_3_, 600 MHz) and ^13^C NMR (CDCl_3_, 150 MHz), see Table 4; HRESIMS: *m/z* 1030.3778 [M+H]^+^ (calcd for C_47_H_60_N_5_O_21_, 1030.3781).

Trioxacarcin G (**20**): yellow amorphous powder. ${\left[\alpha \right]}_{D}^{25}$ −140.0° (c 0.02, ACN); UV (MeOH) λ_max_ (log ε) 232 (4.19), 271 (4.25), 400 (3.71) nm; IR *v*_max_: 3439, 2933, 2851, 1730, 1623, 1384, 1233, 1085, 998 cm^−1^; ^1^H NMR (CDCl_3_, 600 MHz) and ^13^C NMR (CDCl_3_, 150 MHz), see Table 4; HRESIMS: *m/z* 914.3644 [M+NH_4_]^+^ (calcd for C_42_H_60_NO_21_, 914.3658).

## 5. Conclusions

Mangrove actinomycetia are considered one of the promising sources of novel biologically active compounds. In this study, a total of 521 actinomycetial strains were isolated from underexplored mangrove soils collected in Leizhou Peninsular, China. Our integrative strategies using taxonomical information, bioactivity, and metabolomics tools (PCA, OPLS-DA, molecular networking) for dereplication allowed us to prioritize two *Streptomyces* strains (H37, M22) with the potential to produce new antibiotics. Two new trioxacarcins were isolated from the scale-up fermentation broth of *Streptomyces* sp. M22. Our study demonstrated that modern metabolomics tools greatly assist classic antibiotic discovery for strain prioritization and improve the efficiency of novel antibiotics discovery. Our data also highlighted that the mangrove in Leizhou Peninsular is an unexploited source with rich microbial diversity and bioactive actinomycetia. In summary, the new strategies presented in this research could set an example to accelerate new antibiotics discovery from mangroves and other highly productive sources, such as rainforests.

## Figures and Tables

**Table 1:** Information of 23 isolates selected for metabolomics analyses and their antimicrobial activity against drug-resistant *S. aureus* (MRSA) and drug-sensitive *A. baumannii*.

Isolates ID	GenBank Accession No.	Top-Hit Taxon (Pairwise Similarity, %)	Diameter of Inhibition Zone (mm)	
			*A. baumannii_S*	*S. aureus_R*
H7	MW724538	*Streptomyces malaysiensis* NBRC 16446^T^ (100.00)	-	24
H8	MW724539	*Streptomyces bungoensis* DSM 41781^T^ (99.62)	-	10
H37	MW724543	*Streptomyces thermoviolaceus subsp. Thermoviolaceus* DSM 40443^T^ (98.50)	13	17
H51	MW724550	*Streptomyces griseochromogenes* ATCC 14511^T^ (99.38)	-	23
H55	MW724552	*Streptomyces galbus* DSM 40089^T^ (99.50)	-	24
H65	MW724554	*Streptomyces griseoincarnatus* LMG 19316^T^ (100.00)	-	18
H112	MW724556	*Gordonia polyisoprenivorans* NBRC 16320^T^ (98.54)	-	10
H121	MW724559	*Rhodococcus zopfii* NBRC 100606^T^ (100.00)	-	10
M28	MW724573	*Streptomyces sundarbansensis* MS1/7^T^ (99.76)	-	11
M106	MW724593	*Streptomyces geysiriensis* NBRC 15413^T^ (99.75)	10	20
M111	MW724597	*Streptomyces smyrnaeus* SM3501^T^ (99.74)	-	16
Y2	MW724603	*Streptomyces hygroscopicus subsp. hygroscopicus* NBRC 13472^T^ (99.63)	-	21
Y4	MW724605	*Streptomyces albogriseolus* NRRL B-1305^T^ (100.00)	10	26
Y9	MW724607	*Streptomyces pluripotens* MUSC 135^T^ (99.87)	17	23
Y15	MW724610	*Streptomyces cellulosae* NBRC 13027^T^ (100.00)	-	11
Y20	MW724611	*Streptomyces levis* NBRC 15423^T^ (99.75)	-	18
Y46	MW724616	*Streptomyces antibioticus* NBRC 12838^T^ (100.00)	9	25
Y101	MW724624	*Micromonospora humi* DSM 45647^T^ (99.61)	-	10
Y198	MW724634	*Micromonospora pallida* DSM 43817^T^ (99.13)	-	14
H12	MW724637	*Streptomyces similanensis* KC-106^T^ (99.13)	10	23
H18	MW724638	*Streptomyces seoulensis* NRRL B-24310^T^ (100.00)	8	20
M22	MW724664	*Streptomyces shenzhenensis* 172115^T^ (99.88)	16	10
M45	MW724671	*Demequina salsinemoris* NBRC 105323^T^ (99.63)	-	17

Paper disk diameter, 6 mm; -, no inhibitory zone; *A. baumannii*, *Acinetobacter baumannii*; *S. aureus*, *Staphylococcus aureus*; *S*, sensitive; *R*, drug resistant.

**Table 2:** The putative metabolites for compounds **1**–**9** against the NPAtlas (Hit 1) and StreptomeDB (Hit 2) databases in the Progenesis QI v3.0 software.

Peak ID	Compound	Isolate No. ^a^	R_t_ (min)	Neutral Mass	Observed *m/z*	UV-Vis (nm) ^b^	Hit 1	Hit 2	Predicted MF ^e^
**1**	12.88_1268.6095n	H12,Y46	12.88	1268.6095	1269.6168	245, 442	Actinomycin Z4 (**1a**)		C_62_H_84_N_12_O_17_
								Actinomycin X2(V) (**1b**)	C_62_H_84_N_12_O_17_
**2**	12.69_1254.6282n	H12,Y46	12.69	1254.6282	1255.6355	245, 442	Actinomycin D (**2a**)	Actinomycin D (**2a**)	C_62_H_86_N_12_O_16_
**3**	11.11_1270.6216n	H12,Y46	11.11	1270.6216	1271.6289	245, 442	Actinomycin X0δ (**3a**)		C_62_H_86_N_12_O_17_
								Actinomycin X0β (**3b**)	C_62_H_86_N_12_O_17_
**4**	16.71_724.4749n	H7,Y2	16.71	724.4749	747.4654	- ^c^	Nigericin (**4a**)	Nigericin (**4a**)	C_40_H_68_O_11_
							Epinigericin (**4b**)		C_40_H_68_O_11_
							Abierixin (**4c**)		C_40_H_68_O_11_
**5**	9.35_453.2153n ^d^	H7,Y2	9.35	453.2153	454.2226	257, 308	Diaporisoindole D (**5a**)		C_26_H_31_NO_6_
							Diaporisoindole E (**5b**)		C_26_H_31_NO_6_
							Aniduquinolone B (**5c**)		C_26_H_31_NO_6_
							NPA001400 (**5d**)		C_26_H_31_NO_6_
							NPA020460 (**5e**)		C_26_H_31_NO_6_
**6**	18.21_708.4796n	H7,Y2	18.21	708.4796	731.4806	- ^c^	Grisorixin (**6a**)	Grisorixin (**6a**)	C_40_H_68_O_10_
								Epigrisorixin (**6b**)	C_40_H_68_O_10_
							Ushikulide A (**6c**)	Ushikulide A (**6c**)	C_40_H_68_O_10_
							Ushikulide B (**6d**)	Ushikulide B (**6d**)	C_40_H_68_O_10_
**7**	9.35_546.2571n	H7,Y2	9.35	546.2571	564.2910	257, 308		17-O-demethyl-geldanamycin (**7**)	C_28_H_38_N_2_O_9_
**8**	9.46_548.2717n	H7,Y2	9.46	548.2717	566.3055	257, 308	4,5-dihydro-17-O-demethyl-geldanamycin (**8a**)	4,5-dihydro-17-O-demethyl-geldanamycin (**8a**)	C_28_H_40_N_2_O_9_
							17-O-demethylgeldanamycin hydroquinone (**8b**)		
							Herbimycin F (**8c**)		
							Antimycin A1a (**8d**)	Antimycin A1a (**8d**)	
								Antimycin A1 (**8e**)	
							Antimycin A12 (**8f**)		
							Antimycin A13 (**8g**)		
							Antimycin A19 (**8h**)	Antimycin A19 (**8h**)	
								Deformylantimycin A2a (**8i**)	
**9**	10.91_560.2719n	H7,Y2	10.91	560.2719	578.3062	257, 308		Geldanamycin (**9a**)	C_29_H_40_N_2_O_9_
							17-hydroxymethyl-17-demethoxygeldanamycin (**9b**)	17-hydroxymethyl-17-demethoxygeldanamycin (**9b**)	C_29_H_40_N_2_O_9_
							17-formyl-17-demethoxy18-O,21-O-dihydrogeldanamycin (**9c**)	17-formyl-17-demethoxy18-O,21-O-dihydrogeldanamycin (**9c**)	C_29_H_40_N_2_O_9_
							Herbimycin C (**9d**)	Herbimycin C (**9d**)	C_29_H_40_N_2_O_9_

^a^, the isolate with the high intensity; ^b^, re-checked in the LC conditions with ACN-H_2_O; ^c^, no or end absorption; ^d^, false positive; ^e^, molecular formula.

**Table 3:** The putative metabolites for compounds **10**–**19** against the NPAtlas (Hit 1) and StreptomeDB (Hit 2) databases in the Progenesis QI v3.0 software.

Peak ID	Compound	Isolate No. ^a^	R_t_ (min)	Neutral Mass	Observed *m/z*	UV-Vis (nm) ^b^	Hit 1	Hit 2	Predicted MF ^d^
**10**	10.64_900.5435n	H37	10.64	900.5435	901.5508	- ^c^		W341C (**10a**)	C_47_H_80_O_16_
							Cytovaricin (**10b**)	Cytovaricin (**10b**)	C_47_H_80_O_16_
**11**	11.08_928.5742n	H37	11.08	928.5742	929.5815	- ^c^	No hit	No hit	unknown
**12**	7.16_1028.3600*m/z*	M22	7.16	unknown	1028.3600	219, 230 (sh), 270, 397	Gutingimycin (**12a**)		C_47_H_57_N_5_O_21_
							Cyanopeptolin SS (**12b**)		C_40_H_63_N_9_O_17_S_2_
							Micropeptin HU989 (**12c**)		C_41_H_64_ClN_9_O_15_S
**13**	15.40_566.4171n	M22	15.4	566.4171	549.4138	- ^c^	X-14889-D (**13a**)		C_33_H_58_O_7_
							CP-78545 (**13b**)	CP-78545 (**13b**)	C_33_H_58_O_7_
							4-ketozeinoxanthin (**13c**)		C_40_H_54_O_2_
**14**	7.47_1028.3592*m/z*	M22	7.47	unknown	1028.3592	219, 230 (sh), 270, 397	Gutingimycin (**14a**)		C_47_H_57_N_5_O_21_
							Cyanopeptolin SS (**14b**)		C_40_H_63_N_9_O_17_S_2_
							Micropeptin HU989 (**14c**)		C_41_H_64_ClN_9_O_15_S
**15**	9.55_876.2968n	M22	9.55	876.2968	894.3355	232, 270, 399		Trioxacarcin A (**15a**)	C_42_H_52_O_20_
							Nai414-B (**15b**)		C_44_H_55_C_l3_N_2_O_10_
**16**	6.69_1030.3751*m/z*	M22	6.69	unknown	1030.3751	219, 230 (sh), 270, 397	Stremycin A (**16a**)	Stremycin A (**16a**)	
								Kedarcidin (**16b**)	
**17**	7.94_1013.3486*m/z*	M22	7.94	unknown	1013.3486	232, 270, 399	No hit	No hit	unknown
**18**	8.43_894.3132n	M22	8.43	894.3132		232, 270, 399		Trioxacarcin B (**18**)	C_42_H_54_O_21_
**19**	8.89_878.3168n	M22	8.89	878.3168		232, 270, 399		Trioxacarcin C (**19**)	C_42_H_54_O_20_

^a^, the isolate with the high intensity; ^b^, re-checked in the LC conditions with ACN-H_2_O; ^c^, no or end absorption; ^d^, molecular formula.

**Table 4:** ^1^H (600 MHz) and ^13^C (150 MHz) NMR data for compounds **16** and **20** in CDCl_3_.

Position	16		20		Position	16		20	
	*δ*_H_ (multi, *J*, Hz)	*δ*c	*δ*_H_ (multi, *J*, Hz)	*δ*c		*δ*_H_ (multi, *J*, Hz)	*δ*c	*δ*_H_ (multi, *J*, Hz)	*δ*c
1		207.6		203.9	17_A_	5.02 (d, 15.6)	46.2	3.68 (m)	62.8
2	5.50 (br s)	68.2	4.81 (dd, 12.6, 5.4)	68.0	17_B_	4.33 (d, 15.6)		3.68 (m)	
3_A_	2.97 (d, 13.8)	37.1	2.77 (m)	36.7	1′	5.51 (d, 4.2)	100.9	5.35 (d, 4.2)	97.4
3_B_	2.21 (overlapped)		2.22 (overlapped)		2′_A_	1.99 (overlapped)	37.7	1.93 (dd, 15.0, 4.2)	36.5
4	5.34 (br s)	72.2	5.41 (t, 3.0)	66.7	2′_B_	1.92 (d, 15.0)		1.62 (d, 15.0)	
4a		128.4		126.7	3′		67.3		69.0
5	7.48 (s)	116.8	7.50 (br s)	117.1	3′-CH_3_	1.18 (s)	27.3	1.06 (s)	25.8
6		142.5		142.7	4′	5.14 (s)	75.9	4.74 (s)	74.5
6-CH_3_	2.62 (s)	20.2	2.59 (s)	20.3	4′-OCOCH_3_		173.3		170.5
7		113.2		114.2	4′-OCOCH_3_	2.25 (s)	21.1	2.12 (s)	21.0
8		152.7		152.6	5′	4.92 (br s)	62.2	4.53 (q, 6.6)	62.9
8a		114.4		114.2	5′-CH_3_	1.26 (d, 6.6)	16.9	1.23 (d, 6.6)	17.0
9		162.9		162.5	1″	5.64 (d, 3.6)	92.5	5.65 (d, 3.0)	93.7
9a		108.3		108.2	2″_A_	2.21 (overlapped)	33.8	2.28 (overlapped)	33.0
9-OH	13.84 (s)		14.61 (s)		2″_B_	1.76 (d, 15.6)		2.05 (d, 15.0)	
10		144.6		145.3	3″	3.91 (br s)	68.6	3.97 (br s)	68.4
10-OCH_3_	3.95 (s)	62.7	3.84 (s)	62.9	4″		72.1		72.6
10a		135.7		135.5	5″	4.68 (q, 6.6)	66.2	4.60 (q, 6.6)	66.3
11	5.10 (d, 4.2)	69.0	5.13 (d, 3.6)	69.3	5″-CH_3_	1.26 (d, 6.6)	15.7	1.23 (d, 6.6)	15.5
12	5.22 (d, 4.2)	71.5	5.21 (d, 3.6)	71.2	6″	3.99 (q, 6.6)	70.3	3.91 (q, 6.6)	70.7
13		103.3		105.2	7″	1.37 (d, 6.6)	17.8	1.34 (d, 6.6)	18.2
14		84.4		85.0	2‴		151.8		
15		108.8		108.2	4‴		153.8		
16	5.08 (s)	99.6	5.04 (s)	99.9	5‴		108.1		
16-OCH_3_	3.66 (s)	56.1	3.63 (s)	56.4	6‴		157.8		
16-OCH_3_	3.53 (s)	56.4	3.54 (s)	57.3	8‴	8.22 (s)	140.6		

## Data Availability

The sequencing data presented in this study are available in GenBank at NCBI (accession numbers: MW724535-MW724713).

## Supplementary Materials

The following are available online at https://www.mdpi.com/article/10.3390/md19120688/s1, Table S1. Composition of 12 different media used to isolate actinomycetial strains from 13 mangrove soil samples; Table S2. Information on genera distribution of actinomycetial strains isolated from 13 different mangrove soil samples; Table S3. Information on genera distribution of actinomycetial strains recovered from the 12 different cultural media; Table S4. Antibacterial activities of 179 actinomycetial strains by the paper disc diffusion method; Table S5. The trioxacarcin-type antibiotics isolated from the actinomycetial strains; Table S6. The information of mangrove-derived soil samples in different sites of Leizhou Peninsula, China; Figure S1. The UV spectra of three outliers (**1**–**3**) of Y46 and H12. Figure S2. The feature statistic plot of 16.71_724.4749n (**4**) in all samples; Figure S3. The MS/MS fragment pattern of the outlier 16.71_724.4749n (**4**) in sample H7; Figure S4. The MS/MS spectra of three false positive compounds in H7 acquired by DDA method; Figure S5. The MS/MS spectra of three revised compounds (**7**–**9**) in H7 acquired by DDA method; Figure S6. The positive and negative MS spectra of two revised compounds in the LC-MS of H7; Figure S7. The UV spectra of three compounds (**7**–**9)** of H7 eluting with ACN and H_2_O; Figure S8. The MS/MS spectra of two compounds (**10**–**11**) in H37 acquired by MSE method; Figure S9. The UV spectra of seven trioxacarcin-type compounds (**12**, **14**–**19**) in the UPLC-UV-HRMS chromatograms of M22; Figure S10. The MS spectra of seven trioxacarcin-type compounds (**12**, **14**–**19**) in the UPLC-UV-HRMS chromatograms of M22; Figure S11. The VIP score of selected markers in the OPLS-DA model; Figure S12. Molecular network of the cluster containing the compound 15.40_566.4171n (**13**) in M22 extract; Figure S13. Molecular network of the cluster containing the compounds 10.64_900.5435n (**10**) and 11.08_928.5742n (**11**) in H37 extract. Figures S14–S31. The HRESIMS, UV, IR, and NMR spectra of gutingimycin B (**16**) and trioxacarcin G (**20**); Figures S32–S37. The HRESIMS and NMR spectra of gutingimycin (**12**).

## References

[1] Willyard C. (2017). The drug-resistant bacteria that pose the greatest health threats. Nature.

[2] Tacconelli E., Carrara E., Savoldi A., Harbarth S., Mendelson M., Monnet D.L., Pulcini C., Kahlmeter G., Kluytmans J., Carmeli Y. (2018). Discovery, research, and development of new antibiotics: The WHO priority list of antibiotic-resistant bacteria and tuberculosis. Lancet Infect. Dis..

[3] Walsh C.T., Fischbach M.A. (2010). Natural products version 2.0: Connecting genes to molecules. J. Am. Chem. Soc..

[4] Newman D.J., Cragg G.M. (2020). Natural products as sources of new drugs over the nearly four decades from 01/1981 to 09/2019. J. Nat. Prod..

[5] Salam N., Jiao J.Y., Zhang X.T., Li W.J. (2020). Update on the classification of higher ranks in the phylum *Actinobacteria*. Int. J. Syst. Evol. Microbiol..

[6] Procópio R.E., Silva I.R., Martins M.K., Azevedo J.L., Araújo J.M. (2012). Antibiotics produced by *Streptomyces*. Braz. J. Infect. Dis..

[7] Watve M.G., Tickoo R., Jog M.M., Bhole B.D. (2001). How many antibiotics are produced by the genus *Streptomyces*?. Arch. Microbiol..

[8] Clardy J., Fischbach M.A., Walsh C.T. (2006). New antibiotics from bacterial natural products. Nat. Biotechnol..

[9] Genilloud O. (2017). Actinomycetes: Still a source of novel antibiotics. Nat. Prod. Rep..

[10] Kamjam M., Sivalingam P., Deng Z., Hong K. (2017). Deep sea actinomycetes and their secondary metabolites. Front. Microbiol..

[11] Mohammadipanah F., Wink J. (2015). Actinobacteria from arid and desert habitats: Diversity and biological activity. Front. Microbiol..

[12] Tian Y., Li Y.L., Zhao F.C. (2017). Secondary metabolites from polar organisms. Mar. Drugs.

[13] Xu D.B., Ye W.W., Han Y., Deng Z.X., Hong K. (2014). Natural products from mangrove actinomycetes. Mar. Drugs.

[14] Azman A.S., Othman I., Velu S.S., Chan K.G., Lee L.H. (2015). Mangrove rare actinobacteria: Taxonomy, natural compound, and discovery of bioactivity. Front. Microbiol..

[15] Ancheeva E., Daletos G., Proksch P. (2018). Lead compounds from mangrove-associated microorganisms. Mar. Drugs.

[16] Hong K., Gao A.H., Xie Q.Y., Gao H.G., Zhuang L., Lin H.P., Yu H.P., Li J., Yao X.S., Goodfellow M. (2009). Actinomycetes for marine drug discovery isolated from mangrove soils and plants in China. Mar. Drugs.

[17] Tong L.Y. (2011). Isolation and Identification of Actinomycetes from Soil of Root System of Mangrove Forest in Zhanjiang.

[18] Xu M., Li J., Dai S.J., Gao C.Y., Liu J.M., Tuo L., Wang F.F., Li X.J., Liu S.W., Jiang Z.K. (2016). Study on diversity and bioactivity of actinobacteria isolated from mangrove plants collected from Zhanjiang in Guangdong Province. Chin. J. Antibiot..

[19] Betancur L.A., Naranjo-Gaybor S.J., Vinchira-Villarraga D.M., Moreno-Sarmiento N.C., Maldonado L.A., Suarez-Moreno Z.R., Acosta-González A., Padilla-Gonzalez G.F., Puyana M., Castellanos L. (2017). Marine actinobacteria as a source of compounds for phytopathogen control: An integrative metabolic-profiling/bioactivity and taxonomical approach. PLoS ONE.

[20] Sebak M., Saafan A.E., AbdelGhani S., Bakeer W., El-Gendy A.O., Espriu L.C., Duncan K., Edrada-Ebel R. (2019). Bioassay- and metabolomics-guided screening of bioactive soil actinomycetes from the ancient city of Ihnasia, Egypt. PLoS ONE.

[21] Hou Y.P., Braun D.R., Michel C.R., Klassen J.L., Adnani N., Wyche T.P., Bugni T.S. (2012). Microbial strain prioritization using metabolomics tools for the discovery of natural products. Anal. Chem..

[22] Villas-Bôas S.G., Mas S., Åkesson M., Smedsgaard J., Nielsen J. (2005). Mass spectrometry in metabolome analysis. Mass Spectrom. Rev..

[23] Samat N., Tan P.J., Shaari K., Abas F., Lee H.B. (2014). Prioritization of natural extracts by LC-MS-PCA for the identification of new photosensitizers for photodynamic therapy. Anal. Chem..

[24] Forner D., Berrué F., Correa H., Duncan K., Kerr R.G. (2013). Chemical dereplication of marine actinomycetes by liquid chromatography–high resolution mass spectrometry profiling and statistical analysis. Anal. Chim. Acta.

[25] Gill K.A., Berrué F., Arens J.C., Kerr R.G. (2014). Isolation and structure elucidation of cystargamide, a lipopeptide from *Kitasatospora cystarginea*. J. Nat. Prod..

[26] Stewart A.K., Ravindra R., Van Wagoner R.M., Wright J.L.C. (2018). Metabolomics-guided discovery of microginin peptides from cultures of the cyanobacterium *Microcystis aeruginosa*. J. Nat. Prod..

[27] Gill K.A., Berrué F., Arens J.C., Carr G., Kerr R.G. (2015). Cystargolides, 20S proteasome inhibitors isolated from *Kitasatospora cystarginea*. J. Nat. Prod..

[28] Abdelmohsen U.R., Cheng C., Viegelmann C., Zhang T., Grkovic T., Ahmed S., Quinn R.J., Hentschel U., Edrada-Ebel R. (2014). Dereplication strategies for targeted isolation of new antitrypanosomal actinosporins A and B from a marine sponge associated-*Actinokineospora* sp. EG49. Mar. Drugs.

[29] Tawfike A., Attia E.Z., Desoukey S.Y., Hajjar D., Makki A.A., Schupp P.J., Edrada-Ebel R., Abdelmohsen U.R. (2019). New bioactive metabolites from the elicited marine sponge-derived bacterium *Actinokineospora spheciospongiae* sp. nov. AMB Express.

[30] Yang J.Y., Sanchez L.M., Rath C.M., Liu X.T., Boudreau P.D., Bruns N., Glukhov E., Wodtke A., de Felicio R., Fenner A. (2013). Molecular networking as a dereplication strategy. J. Nat. Prod..

[31] Tangerina M.M.P., Furtado L.C., Leite V.M.B., Bauermeister A., Velasco-Alzate K., Jimenez P.C., Garrido L.M., Padilla G., Lopes N.P., Costa-Lotufo L.V. (2020). Metabolomic study of marine *Streptomyces* sp.: Secondary metabolites and the production of potential anticancer compounds. PLoS ONE.

[32] Wang M.X., Carver J.J., Phelan V.V., Sanchez L.M., Garg N., Peng Y., Nguyen D.D., Watrous J., Kapono C.A., Luzzatto-Knaan T. (2016). Sharing and community curation of mass spectrometry data with Global Natural Products Social Molecular Networking. Nat. Biotechnol..

[33] Van Santen J.A., Jacob G., Singh A.L., Aniebok V., Balunas M.J., Bunsko D., Neto F.C., Castaño-Espriu L., Chang C., Clark T.N. (2019). The Natural products atlas: An open access knowledge base for microbial natural products discovery. ACS Cent. Sci..

[34] Moumbock A.F., Gao M., Qaseem A., Li J., Kirchner P.A., Ndingkokhar B., Bekono B.D., Simoben C.V., Babiaka S.B., Malange Y.I. (2020). StreptomeDB 3.0: An updated compendium of streptomycetes natural products. Nucleic Acids Res..

[35] Zhang X.F., Ye X.W., Chai W.Y., Lian X.Y., Zhang Z.Z. (2016). New metabolites and bioactive actinomycins from marine-derived *Streptomyces* sp. ZZ338. Mar. Drugs.

[36] Lackner H., Bahner I., Shigematsu N., Pannell L.K., Mauger A.B. (2000). Structures of five components of the actinomycin Z complex from *Streptomyces fradiae*, two of which contain 4-chlorothreonine. J. Nat. Prod..

[37] David L., Ayala H.L., Tabet J.C. (1985). Abierixin, a new polyether antibiotic. Production, structural determination and biological activities. J. Antibiot..

[38] Zhao X., Wang B., Xie K.Z., Liu J.Y., Zhang Y.Y., Wang Y.J., Guo Y.W., Zhang G.X., Dai G.J., Wang J.Y. (2018). Development and comparison of HPLC-MS/MS and UPLC-MS/MS methods for determining eight coccidiostats in beef. J. Chromatogr. B Analyt. Technol. Biomed. Life Sci..

[39] Martínez-Villalba A., Moyano E., Galceran M.T. (2009). Fast liquid chromatography/multiple-stage mass spectrometry of coccidiostats. Rapid Commun. Mass Spectrom..

[40] Takahashi K., Yoshihara T., Kurosawa K. (2005). Ushikulides A and B, immunosuppressants produced by a strain of *Streptomyces* sp. J. Antibiot..

[41] Mouslim J., Cuer A., David L., Tabet J.C. (1993). Epigrisorixin, a new polyether carboxylic antibiotic. J. Antibiot..

[42] Gachon P., Kergomard A., Veschambre H., Esteve C., Staron T. (1970). Grisorixin, a new antibiotic related to nigericin. J. Chem. Soc. D.

[43] Cui H., Liu Y.N., Li J., Huang X.S., Yan T., Cao W.H., Liu H.J., Long Y.H., She Z.G. (2018). Diaporindenes A–D: Four unusual 2,3-dihydro-1H-indene analogues with anti-inflammatory activities from the mangrove endophytic fungus *Diaporthe* sp. SYSU-HQ3. J. Org. Chem..

[44] An C.Y., Li X.M., Luo H., Li C.S., Wang M.H., Xu G.M., Wang B.G. (2013). 4-Phenyl-3,4-dihydroquinolone derivatives from *Aspergillus nidulans* MA-143, an endophytic fungus isolated from the mangrove plant *Rhizophora stylosa*. J. Nat. Prod..

[45] Sato K., Goda Y., Sasaki S.S., Shibata H., Maitani T., Yamada T. (1997). Identification of major pigments containing D-amino acid units in commercial Monascus pigments. Chem. Pharm. Bull..

[46] Guo J., Huan T. (2020). Comparison of Full-Scan, Data-Dependent, and Data-Independent Acquisition modes in liquid chromatography–mass spectrometry based untargeted metabolomics. Anal. Chem..

[47] Nikolskiy I., Mahieu N.G., Chen Y., Tautenhahn R., Patti G.J. (2013). An untargeted metabolomic workflow to improve structural characterization of metabolites. Anal. Chem..

[48] Helm S., Baginsky S. (2018). MSE for label-free absolute protein quantification in complex proteomes. Methods Mol. Biol..

[49] Baksh A., Kepplinger B., Isah H.A., Probert M.R., Clegg W., Wills C., Goodfellow M., Errington J., Allenby N., Hall M.J. (2017). Production of 17-*O*-demethyl-geldanamycin, a cytotoxic ansamycin polyketide, by *Streptomyces hygroscopicus* DEM20745. Nat. Prod. Res..

[50] Lin H.N., Wang K.L., Wu Z.H., Tian R.M., Liu G.Z., Xu Y. (2017). Biological and chemical diversity of bacteria associated with a marine flatworm. Mar. Drugs.

[51] DeBoer C., Meulman P.A., Wnuk R.J., Peterson D.H. (1970). Geldanamycin, a new antibiotic. J. Antibiot..

[52] Maskey R.P., Sevvana M., Usón I., Helmke E., Laatsch H. (2004). Gutingimycin: A highly complex metabolite from a marine *Streptomycete*. Angew. Chem. Int. Ed..

[53] Tamaoki T., Shirahata K., Iida T., Tomita F. (1981). Trioxacarcins, novel antitumor antibiotics. II. Isolation, physico-chemical properties and mode of action. J. Antibiot..

[54] Maoka T., Takemura M., Tokuda H., Suzuki N., Misawa N. (2014). 4-Ketozeinoxanthin, a novel carotenoid produced in *Escherichia coli* through metabolic engineering using carotenogenic genes of bacterium and liverwort. Tetrahedron Lett..

[55] Wong W.R., Oliver A.G., Linington R.G. (2012). Development of antibiotic activity profile screening for the classification and discovery of natural product antibiotics. Chem. Biol..

[56] Kihara T., Kusakabe H., Nakamura G., Sakurai T., Isono K. (1981). Cytovaricin, a novel antibiotic. J. Antibiot..

[57] Cai R.S., Wu J.M. (1985). Biochemical and biological characterization of ionophorous antibiotic W341. Chin. J. Antibiot..

[58] Crusemann M., O’Neill E.C., Larson C.B., Melnik A.V., Floros D.J., da Silva R.R., Jensen P.R., Dorrestein P.C., Moore B.S. (2016). Prioritizing natural product diversity in a collection of 146 bacterial strains based on growth and extraction protocols. J. Nat. Prod..

[59] Tomita F., Tamaoki T., Shirahata K., Iida T., Morimoto M., Fujimoto K. (1985). Antibiotic substances DC-45, and their use as medicaments. U.S. Patent.

[60] Maskey R.P., Helmke E., Kayser O., Fiebig H.H., Maier A., Busche A., Laatsch H. (2004). Anti-cancer and antibacterial trioxacarcins with high anti-malaria activity from a marine Streptomycete and their absolute stereochemistry. J. Antibiot..

[61] Nicolaou K.C., Cai Q., Sun H., Qin B., Zhu S. (2016). Total synthesis of trioxacarcins DC-45-A1, A, D, C, and C7″-epi-C and full structural assignment of trioxacarcin C. J. Am. Chem. Soc..

[62] Li F.N., Liu S.W., Lu Q.P., Zheng H.Y., Osterman I.A., Lukyanov D.A., Sergiev P.V., Dontsova O.A., Liu S.S., Ye J.J. (2019). Studies on antibacterial activity and diversity of cultivable actinobacteria isolated from mangrove soil in futian and maoweihai of China. Evid. Based Complement. Alternat. Med..

[63] Lu Q.P., Ye J.J., Huang Y.M., Liu D., Liu L.F., Dong K., Razumova E.A., Osterman I.A., Sergiev P.V., Dontsova O.A. (2019). Exploitation of potentially new antibiotics from mangrove actinobacteria in Maowei Sea by combination of multiple discovery strategies. Antibiotics.

[64] Feling R.H., Buchanan G.O., Mincer T.J., Kauffman C.A., Jensen P.R., Fenical W. (2003). Salinosporamide A: A Highly Cytotoxic Proteasome Inhibitor from a Novel Microbial Source, a Marine Bacterium of the New Genus *Salinospora*. Angew. Chem. Int. Ed..

[65] Chauhan D., Catley L., Li G., Podar K., Hideshima T., Velankar M., Mitsiades C., Mitsiades N., Yasui H., Letai A. (2005). A novel orally active proteasome inhibitor induces apoptosis in multiple myeloma cells with mechanisms distinct from Bortezomib. Cancer Cell.

[66] Ding L., Münch J., Goerls H., Maier A., Fiebig H.-H., Lin W.-H., Hertweck C. (2010). Xiamycin, a pentacyclic indolosesquiterpene with selective anti-HIV activity from a bacterial mangrove endophyte. Bioorg. Med. Chem. Lett..

[67] Gao X.M., Han W.D., Liu S.Q. (2009). The mangrove and its conservation in Leizhou Peninsula, China. J. For. Res..

[68] Li M.S., Mao L.J., Shen W.J., Liu S.Q., Wei A.S. (2013). Change and fragmentation trends of Zhanjiang mangrove forests in southern China using multi-temporal Landsat imagery (1977–2010). Estuar. Coast. Shelf Sci..

[69] Hazarika S.N., Thakur D., Amaresan N., Kumar M.S., Annapurna K., Kumar K., Sankaranarayanan A. (2020). Chapter 21—Actinobacteria. Beneficial Microbes in Agro-Ecology.

[70] Harikrishnan H., Shanmugaiah V., Nithya K., Balasubramanian N., Sharma M.P., Gachomo E.W., Kotchoni S.O. (2016). Enhanced production of phenazine-like metabolite produced by *Streptomyces aurantiogriseus* VSMGT1014 against rice pathogen, Rhizoctonia solani. J. Basic Microbiol..

[71] Cheng C., MacIntyre L., Abdelmohsen U.R., Horn H., Polymenakou P.N., Edrada-Ebel R., Hentschel U. (2015). Biodiversity, anti-trypanosomal activity screening, and metabolomic profiling of actinomycetes isolated from mediterranean sponges. PLoS ONE.

[72] Genilloud O., González I., Salazar O., Martín J., Tormo J.R., Vicente F. (2011). Current approaches to exploit actinomycetes as a source of novel natural products. J. Ind. Microbiol. Biotechnol..

[73] Liu X.Y., Ashforth E., Ren B., Song F.H., Dai H.Q., Liu M., Wang J., Xie Q., Zhang L.X. (2010). Bioprospecting microbial natural product libraries from the marine environment for drug discovery. J. Antibiot..

[74] Stuart K.A., Welsh K., Walker M.C., Edrada-Ebel R. (2020). Metabolomic tools used in marine natural product drug discovery. Expert Opin. Drug Discov..

[75] Gu R.H., Rybalov L., Negrin A., Morcol T., Long W.W., Myers A.K., Isaac G., Yuk J., Kennelly E.J., Long C.L. (2019). Metabolic profiling of different parts of *Acer truncatum* from the Mongolian Plateau using UPLC-QTOF-MS with comparative bioactivity assays. J. Agric. Food. Chem..

[76] Tomita F., Tamaoki T., Morimoto M., Fujimoto K. (1981). Trioxacarcins, novel antitumor antibiotics. I. Producing organism, fermentation and biological activities. J. Antibiot..

[77] Magauer T., Smaltz D.J., Myers A.G. (2013). Component-based syntheses of trioxacarcin A, DC-45-A1 and structural analogues. Nat. Chem..

[78] Arshad M., Sharif A., Ahmed E., Bariyah S. (2019). Trioxacarcins as a promising class of anticancer drugs. World J. Pharm. Pharm. Sci..

[79] Nicolaou K.C., Chen P., Zhu S., Cai Q., Erande R.D., Li R., Sun H., Pulukuri K.K., Rigol S., Aujay M. (2017). Streamlined total synthesis of trioxacarcins and its application to the design, synthesis, and biological evaluation of analogues thereof. discovery of simpler designed and potent trioxacarcin analogues. J. Am. Chem. Soc..

[80] Nicolaou K.C., Cai Q., Qin B., Petersen M.T., Mikkelsen R.J., Heretsch P. (2015). Total synthesis of trioxacarcin DC-45-A2. Angew. Chem. Int. Ed. Engl..

[81] Zhang M., Hou X.-F., Qi L.-H., Yin Y., Li Q., Pan H.-X., Chen X.-Y., Tang G.-L. (2015). Biosynthesis of trioxacarcin revealing a different starter unit and complex tailoring steps for type II polyketide synthase. Chem. Sci..

[82] Shen Y., Nie Q.-Y., Yin Y., Pan H.-X., Xu B., Tang G.-L. (2019). Production of a trioxacarcin analog by introducing a C-3 dehydratase into deoxysugar biosynthesis. Acta Biochim. Biophys. Sin..

[83] Qi L.-H., Zhang M., Pan H.-X., Chen X.-D., Tang G.-L. (2014). Production of a trioxacarcin analogue by engineering of its biosynthetic pathway. Chin. J. Org. Chem..

[84] Yin Y., Shen Y., Meng S., Zhang M., Pan H.-X., Tang G.-L. (2020). Characterization of a membrane-bound O-acetyltransferase involved in trioxacarcin biosynthesis offers insights into its catalytic mechanism. Chin. J. Chem.

[85] Yang K., Qi L.H., Zhang M., Hou X.F., Pan H.X., Tang G.L., Wang W., Yuan H. (2015). The SARP family regulator Txn9 and two-component response regulator Txn11 are key activators for trioxacarcin biosynthesis in *Streptomyces bottropensis*. Curr. Microbiol..

[86] Pfoh R., Laatsch H., Sheldrick G.M. (2008). Crystal structure of trioxacarcin A covalently bound to DNA. Nucleic Acids Res..

[87] Pröpper K., Dittrich B., Smaltz D.J., Magauer T., Myers A.G. (2014). Crystalline guanine adducts of natural and synthetic trioxacarcins suggest a common biological mechanism and reveal a basis for the instability of trioxacarcin A. Bioorg. Med. Chem. Lett..

[88] Macintyre L., Zhang T., Viegelmann C., Martinez I.J., Cheng C., Dowdells C., Abdelmohsen U.R., Gernert C., Hentschel U., Edrada-Ebel R.A. (2014). Metabolomic tools for secondary metabolite discovery from marine microbial symbionts. Mar. Drugs.

[89] Zhou S.Q., Huang X.L., Huang D.Y., Hu X.W., Chen J.L. (2010). A rapid method for extracting DNA from actinomycetes by Chelex-100. Shengwu Jishu Tongbao.

[90] Yoon S.H., Ha S.M., Kwon S., Lim J., Kim Y., Seo H., Chun J. (2017). Introducing EzBioCloud: A taxonomically united database of 16S rRNA gene sequences and whole-genome assemblies. Int. J. Syst. Evol. Microbiol..

[91] Kumar S., Stecher G., Tamura K. (2016). MEGA7: Molecular evolutionary genetics analysis version 7.0 for bigger datasets. Mol. Biol. Evol..

[92] Saitou N., Nei M. (1987). The neighbor-joining method: A new method for reconstructing phylogenetic trees. Mol. Biol. Evol..

[93] Letunic I., Bork P. (2019). Interactive Tree Of Life (iTOL) v4: Recent updates and new developments. Nucleic Acids Res..

[94] Chambers M.C., Maclean B., Burke R., Amodei D., Ruderman D.L., Neumann S., Gatto L., Fischer B., Pratt B., Egertson J. (2012). A cross-platform toolkit for mass spectrometry and proteomics. Nat. Biotechnol..

[95] Shannon P., Markiel A., Ozier O., Baliga N.S., Wang J.T., Ramage D., Amin N., Schwikowski B., Ideker T. (2003). Cytoscape: A software environment for integrated models of biomolecular interaction networks. Genome Res..

