# Supplementary material for: Metabolomics Tools Assisting Classic Screening Methods in Discovering New Antibiotics from Mangrove Actinomycetia in Leizhou Peninsula
Source: Mar Drugs. 2021 Dec 1;19(12):688. doi: 10.3390/md19120688 (PMC8707991; doi:10.3390/md19120688)
Supplement: Supplementary file 1 [file marinedrugs-19-00688-s001.zip › marinedrugs-1389549 SI fianl -lqp.pdf]

Supplementary Materials

Metabolomics Tools Assisting Classic Screening Methods in Discovering New Antibiotics from Mangrove Actinomycetia in Leizhou Peninsula

Qin-Pei Lu [1,2,†], Yong-Mei Huang [3,4,†], Shao-Wei Liu [1,2], Gang Wu [1,2], Qin Yang [1,2], Li-Fang Liu [1,2], Hai-Tao Zhang[3], Yi Qi [3,4], Ting Wang [1,2], Zhong-Ke Jiang [1,2], Jun-Jie Li [3], Hao Cai [5], Xiu-Jun Liu [5], Hui Luo [3,4,*] and Cheng-Hang Sun [1,2,*]

[1] Department of Microbial Chemistry, Institute of Medicinal Biotechnology, Chinese Academy of Medical Sciences & Peking Union Medical College, Beijing 100050, China; qinpei89@hotmail.com (Q.-P.L.); liushaowei3535@163.com (S.-W.L.); gangwu@aliyun.com (G.W.); tingwang0707@imb.pumc.edu.cn (T.W.); yangqin@imb.pumc.edu.cn (Q.Y.); LiuLiFang@imb.pumc.edu.cn (L.-F.L.); jiangzhongke@126.com (Z.-K.J.)
[2] Beijing Key Laboratory of Antimicrobial Agents, Institute of Medicinal Biotechnology, Chinese Academy of Medical Sciences & Peking Union Medical College, Beijing 100050, China.
[3] The Key Lab of Zhanjiang for R&D Marine Microbial Resources in the Beibu Gulf Rim, Marine Biomedical Research Institute, Guangdong Medical University, Zhanjiang 524023, China; huangym@gdmu.edu.cn (Y.-M.H.); taohaizhang33@163.com (H.-T.Z.); qiyi7272@gdmu.edu.cn (Y.Q.); jjleeee@gdmu.edu.cn (J.-J.L.)
[4] Marine Biomedical Research Institute of Guangdong Zhanjiang, Zhanjiang 524023, China.
[5] Department of Oncology, Institute of Medicinal Biotechnology, Chinese Academy of Medical Sciences & Peking Union Medical College, Beijing 100050, China; caihao@imb.pumc.edu.cn (H.C.); Liuxiujun2000@imb.pumc.edu.cn (X.-J. L.)
* Correspondence: chenghangsun@hotmail.com or sunchenghang@imb.pumc.edu.cn (C.-H.S.); Tel.: +86-10-63165272 (C.-H.S.); luohui@gdmu.edu.cn (H.L.)
† These authors contributed equally to this work.

Content

Table S1. Composition of 12 different media used to isolate actinomycetial strains from 13 mangrove soil samples.
Table S2. Information on genera distribution of actinomycetial strains isolated from 13 different mangrove soil samples.
Table S3. Information on genera distribution of actinomycetial strains recovered from the 12 different cultural media.
Table S4. Antibacterial activities of 179 actinomycetial strains by the paper disc diffusion method.
Table S5. The trioxacarcin-type antibiotics isolated from the actinomycetial strains.
Table S6. The information of mangrove-derived soil samples in different sites of Leizhou Peninsula, China.
Figure S1. The UV spectra of three outliers (**1**-**3**) of samples Y46 and H12.
Figure S2. The feature statistic plot of 16.71_724.4749n (**4**) in all samples.
Figure S3. The MS/MS fragment pattern of the outlier 16.71_724.4749n (**4**) in sample H7.
Figure S4. The MS/MS spectra of three false positive compounds in H7 acquired by DDA method.
Figure S5. The MS/MS spectra of three revised compounds (**7**-**9**) in H7 acquired by DDA method.
Figure S6. The positive and negative MS spectra of two revised compounds in the LC-MS of H7.
Figure S7. The UV spectra of three compounds (**7**-**9)** of H7 eluting with ACN and H_2O.
Figure S8. The MS/MS spectra of two compounds (**10**-**11**) in H37 acquired by MSE method.
Figure S9. The UV spectra of seven trioxacarcin-type compounds (**12**, **14**-**19**) in the UPLC-UV-HRMS chromatograms of M22.
Figure S10. The MS spectra of seven trioxacarcin-type compounds (**12**, **14**-**19**) in the UPLC-UV-HRMS chromatograms of M22.
Figure S11. The VIP score of selected markers in the OPLS-DA model.
Figure S12. Molecular network of the cluster containing the compound 15.40_566.4171n (**13**) in M22 extract.
Figure S13. Molecular network of the cluster containing the compounds 10.64_900.5435n (**10**) and 11.08_928.5742n (**11**) in H37 extract.
Figure S14. The UV spectrum of compound **16**.
Figure S15. The HRESIMS of compound **16**.
Figure S16. The IR spectrum of compound **16**.
Figure S17. The ^{1}H NMR (600 MHz) spectrum of compound **16** in $CDCl_3$.
Figure S18. The ^{13}C NMR (150 MHz) spectrum of compound **16** in $CDCl_3$.
Figure S19. The DEPT spectrum of compound **16**.
Figure S20. The ^{1}H-^{1}H COSY spectrum of compound **16**.
Figure S21. The HSQC spectrum of compound **16**.
Figure S22. The HMBC spectrum of compound **16**.
Figure S23. The UV spectrum of compound **20**.

Figure S24. The HRESIMS of compound **20**.
Figure S25. The IR spectrum of compound **20**.
Figure S26. The ^{1}H NMR (600 MHz) spectrum of compound **20** in $CDCl_3$.
Figure S27. The ^{13}C NMR (150 MHz) spectrum of compound **20** in $CDCl_3$.
Figure S28.The DEPT spectrum of compound **20**.
Figure S29. The ^{1}H-^{1}H COSY spectrum of compound **20**.
Figure S30. The HSQC spectrum of compound **20**.
Figure S31. The HMBC spectrum of compound **20**.
Figure S32. The HRESIMS of compound **12**.
Figure S33. The ^{1}H NMR (500 MHz) spectrum of compound **12** in $CDCl_3$.
Figure S34. The ^{13}C NMR (125 MHz) spectrum of compound **12** in $CDCl_3$.
Figure S35. The DEPT spectrum of compound **12**.
Figure S36. The HSQC spectrum of compound **12**.
Figure S37. The HMBC spectrum of compound **12**.

Table S1. Composition of 12 different media used to isolate actinomycetial strains from 13 mangrove soil samples.

No.	Name	Composition
M1	ISP 2 medium	Glucose 4.0 g, yeast extract 4.0 g, malt extract 10.0 g, water[a] 1000 mL, agar 15.0 g, pH 7.2-7.4
M2	GPT medium	Glucose 10.0 g, peptone 5.0 g, tryptone 3.0 g, NaCl 5.0 g, water[a] 1000 mL, agar 15.0 g, pH 7.2-7.4
M3	R2A medium	R2A agar 18.1 g, water[a] 1000 mL, pH 7.2-7.4
M4	TP medium	Trehalose 5.0 g, proline 1.0 g, peptone 1.0 g, yeast extract 0.5 g, $(NH_4)_2SO_4$ 1.0 g, NaCl 1.0 g, $CaCl_2$ 2.0 g, K_2HPO_4 1.0 g, chlorhematin 0.01 mg, $MgSO_4{\cdot}7H_2O$ 1.0 g, vitamin mixture[d] 1.0 mL, water[a] 1000 mL, agar 15.0 g, pH 7.2-7.4
M5	ISP 7 medium	Glycerol 15.0 mL, L-tyrosine 0.5 g, L-asparagine 1.0 g, K_2HPO_4 0.5 g, $MgSO_4{\cdot}7H_2O$ 0.5 g, NaCl 0.5 g, $FeSO_4{\cdot}7H_2O$ 0.01 g, trace salt solution[e] 1.0 mL, water[a] 1000 mL, agar 15.0 g, pH 7.2-7.4
M6	Arginine-glycerol medium	Arginine 1.0 g, glycerol 6.0 mL, vitamin mixture 1.0 mL, trace salt solution 1.0 mL, water[a] 1000 mL, agar 15.0 g, pH 7.2-7.4
M7	Starch-casein medium	Soluble starch 10.0 g, casein 0.3 g, KNO_3 2.0 g, $MgSO_4{\cdot}7H_2O$ 0.05 g, NaCl 30.0 g, K_2HPO_4 2.0 g, $CaCO_3$ 0.02 g, $FeSO_4{\cdot}7H_2O$ 0.01 g, water[a] 1000 mL, agar 15.0 g, pH 7.2-7.4
M8	Asparagine-glycerol medium	L-asparagine 1.0 g, glycerol 10.0 mL, K_2HPO_4 1.0 g, trace salt solution 1.0 mL, water[a] 1000 mL, agar 15.0 g, pH 7.2-7.4
M9	1/10 ATCC 172 medium	Soluble starch 2.0 g, glucose 1.0 g, yeast extract 0.5 g, $CaCO_3$ 1.5 g, N-Z-amine 0.5 g, water[a] 1000 mL, agar 15.0 g, pH 7.2-7.4
M10	RH medium	Raffinose 10.0 g, L-histidine 1.0 g, K_2HPO_4 1.0 g, $MgSO_4$ 1.0 g, $FeSO_4{\cdot}7H_2O$ 0.01 g, water[a] 1000 mL, agar 15.0 g, pH 7.2-7.4
M11	modified ISP 2 medium	Glucose 4.0 g, yeast extract 4.0 g, malt extract 10.0 g, *Laminaria japonica* juice (kelp juice) 15 mL, water[b] 985 mL, agar 15.0 g, pH 7.2-7.4
M12	modified TP medium	Trehalose 5.0 g, proline 1.0 g, peptone 1.0 g, yeast extract 0.5 g, $(NH_4)_2SO_4$ 1.0 g, NaCl 1.0 g, $CaCl_2$ 2.0 g, K_2HPO_4 1.0 g, $MgSO_4{\cdot}7H_2O$ 1.0 g, chlorhematin 0.01 mg, vitamin mixture 1.0 mL, fresh coconut juice 10 mL, water[c] 990 mL, agar 15.0 g, pH 7.2-7.4

[a]water: 900.0 mL distilled water and 100.0 mL sea water; [b]water: 885.0 mL distilled water and 100.0 mL sea water; [c]water: 890.0 mL distilled water and 100.0 mL sea water [d]vitamin mixture: thiamine (0.10 g); pyridoxine (0.10 g); riboflavin (0.10 g); niacin (0.10 g); biotin (0.10 g); distilled water (100 mL); [e]Trace salt mixture: $FeSO_4{\cdot}7H_2O$ (0.20 g); $MnCl_2{\cdot}4H_2O$ (0.01 g); $ZnSO_4{\cdot}7H_2O$ (0.01 g); distilled water (100 mL).

Table S2. Information on genera distribution of actinomycetial strains isolated from 13 different mangrove soil samples.

Genus	S1	S2	S3	S4	S5	S6	S7	S8	S9	S10	S11	S12	S13	Isolates
Micromonospora	9	-	4	4	23	11	10	8	20	13	15	3	1	121
Streptomyces	34	8	2	-	15	-	2	22	6	8	8	4	7	116
Microbacterium	2	5	4	2	1	-	-	7	4	4	-	-	7	36
Rhodococcus	7	6	-	-	11	-	-	1	-	-	4	4	2	35
Brachybacterium	-	-	-	-	-	-	1	1	8	1	-	-	17	28
Isoptericola	-	1	-	-	-	-	-	-	13	8	-	-	3	25
Cellulosimicrobium	3	9	4	1	2	-	-	1	-	-	-	-	1	21
Brevibacterium	2	2	2	2	2	-	-	-	2	3	-	-	2	17
Serinibacter	-	-	-	1	-	-	-	3	8	-	-	-	1	13
Agromyces	-	2	2	-	-	-	1	3	-	1	-	1	-	10
Micrococcus	-	-	-	-	-	1	-	6	-	1	1	1	-	10
Mycolicibacterium	2	1	2	-	-	-	-	1	1	2	-	-	1	10
Kocuria	-	1	1	1	-	-	-	3	1	1	1	-	-	9
Gordonia	2	3	-	-	-	-	-	1	-	-	-	-	-	6
Mycobacterium	-	-	-	-	-	-	-	-	-	6	-	-	-	6
Aeromicrobium	1	-	1	-	1	1	-	-	1	-	-	-	-	5
Arthrobacter	-	1	-	-	-	-	1	1	-	-	1	1	-	5
Citricoccus	-	-	-	1	1	-	-	3	-	-	-	-	-	5
Janibacter	2	1	2	-	-	-	-	-	-	-	-	-	-	5
Nocardia	2	-	1	-	-	-	-	-	-	-	2	-	-	5
Corynebacterium	2	-	2	-	-	-	-	-	-	-	-	-	-	4
Glutamicibacter	-	1	1	-	-	-	-	-	-	-	-	-	2	4
Agrococcus	1	-	-	-	-	-	-	2	-	-	-	-	-	3
Intrasporangium	2	-	-	-	-	-	-	-	-	-	-	-	-	2
Kineococcus	-	-	-	-	-	-	-	-	2	-	-	-	-	2
Phycicoccus	-	-	-	-	-	-	-	-	-	-	-	-	2	2
Serinicoccus	-	-	-	-	-	-	-	-	-	-	-	-	2	2
Sinomonas	1	-	1	-	-	-	-	-	-	-	-	-	-	2
Actinomadura	1	-	-	-	-	-	-	-	-	-	-	-	-	1
Actinopolymorpha	-	-	-	-	-	-	-	-	1	-	-	-	-	1
Blastococcus	-	-	-	-	-	-	-	-	-	-	-	1	-	1
Demequina	-	-	-	-	-	-	-	-	-	-	-	-	1	1
Georgenia	-	-	-	1	-	-	-	-	-	-	-	-	-	1
Gulosibacter	1	-	-	-	-	-	-	-	-	-	-	-	-	1
Jonesia	-	-	1	-	-	-	-	-	-	-	-	-	-	1
Leucobacter	-	-	-	-	-	-	-	-	1	-	-	-	-	1
Motilibacter	-	-	-	-	-	-	-	-	1	-	-	-	-	1

Mumia	-	-	-	-	-	-	-	-	1	-	-	-	-	1
Salinibacterium	-	-	-	-	-	1	-	-	-	-	-	-	-	1
Streptacidiphilus	-	-	-	-	-	-	-	-	-	-	1	-	-	1
Isolates	74	41	30	13	56	14	15	63	70	48	33	15	49	521
Genera	17	13	15	8	8	4	5	15	15	11	8	7	14	40

-: No isolate

Table S3. Information on genera distribution of actinomycetial strains recovered from 12 different cultural media.

**Genus**	**M1**	**M2**	**M3**	**M4**	**M5**	**M6**	**M7**	**M8**	**M9**	**M10**	**M11**	**M12**	**Isolates**
Micromonospora	17	23	12	-	15	7	1	10	12	19	5	-	121
Streptomyces	7	6	12	5	21	10	10	18	12	9	5	1	116
Microbacterium	9	1	4	-	1	1	-	1	5	3	10	1	36
Rhodococcus	3	4	7	-	1	5	2	1	3	7	2	-	35
Brachybacterium	3	1	1	3	3	1	4	1	5	-	6	-	28
Isoptericola	7	4	2	-	4	-	-	-	1	1	6	-	25
Cellulosimicrobium	3	2	3	1	3	-	1	1	2	1	3	1	21
Brevibacterium	-	2	3	2	2	2	-	-	4	-	-	2	17
Serinibacter	2	1	1	1	-	1	-	2	3	1	1	-	13
Mycolicibacterium	3	2	-	-	-	-	-	3	-	-	2	-	10
Micrococcus	-	2	-	-	-	3	-	1	-	-	3	1	10
Agromyces	1	-	2	-	-	1	4	-	-	-	1	1	10
Kocuria	-	-	-	-	1	-	-	-	-	1	4	3	9
Mycobacterium	2	-	1	-	-	-	-	-	-	-	3	-	6
Gordonia	-	2	1	-	-	-	-	-	1	1	1	-	6
Aeromicrobium	-	1	2	-	1	-	-	-	-	-	1	-	5
Arthrobacter	1	1	-	-	-	2	-	-	-	-	-	1	5
Citricoccus	1	-	-	-	-	2	-	-	-	-	-	2	5
Janibacter	-	-	1	1	1	-	1	-	-	-	1	-	5
Nocardia	2	-	1	-	-	-	-	1	-	-	1	-	5
Corynebacterium	1	-	1	-	-	-	1	-	-	-	1	-	4
Glutamicibacter	-	-	1	-	-	-	-	-	-	-	-	3	4
Agrococcus	-	1	-	-	-	1	1	-	-	-	-	-	3
Intrasporangium	-	-	-	-	1	1	-	-	-	-	-	-	2
Kineococcus	1	1	-	-	-	-	-	-	-	-	-	-	2
Phycicoccus	-	-	-	-	-	-	-	-	-	-	2	-	2
Sinomonas	1	-	-	-	1	-	-	-	-	-	-	-	2
Serinicoccus	-	-	-	-	-	-	-	-	-	-	2	-	2
Actinomadura	-	-	1	-	-	-	-	-	-	-	-	-	1
Actinopolymorpha	-	-	-	-	1	-	-	-	-	-	-	-	1
Blastococcus	-	-	-	-	-	-	-	-	-	-	1	-	1
Demequina	-	-	-	-	-	-	-	-	-	-	1	-	1
Georgenia	-	-	-	-	-	-	-	-	-	-	-	1	1
Gulosibacter	-	-	-	-	-	-	-	-	1	-	-	-	1

Jonesia	-	-	-	-	-	-	-	-	-	-	-	1	1
Leucobacter	-	-	-	-	-	-	-	-	1	-	-	-	1
Motilibacter	-	1	-	-	-	-	-	-	-	-	-	-	1
Mumia	1	-	-	-	-	-	-	-	-	-	-	-	1
Salinibacterium	-	-	-	-	-	-	-	-	-	-	1	-	1
Streptacidiphilus	-	-	-	-	1	-	-	-	-	-	-	-	1
Isolates	65	55	56	13	57	37	25	39	50	43	63	18	521
Genera	18	17	18	6	15	13	9	10	12	9	23	12	40

-: No isolate

Table S4. Antibacterial activities of 179 actinomycetial strains by the paper disc diffusion method.

Isolates ID	Top-hit taxon (Pairwise similarity, %)	Diameter of Inhibition Zone (mm)											
		E. coli		***P. aeruginosa***		***K. peneumoniae***		***A. baumannii***		***S. aureus***		***E. faecalis***	***E. faecium***
		**S**	**R**	**S**	**R**	**S**	**R**	**S**	**R**	**S**	**R**	**S**	**R**
H3 (MW724537)	*Micromonospora aurantiaca* ATCC 27029^T (100.00)	-	-	-	-	-	-	-	-	-	-	-	-
H7 (MW724538)	*Streptomyces malaysiensis* NBRC 16446^T (100.00)	-	-	-	-	-	-	-	-	19	24	25	25
H8 (MW724539)	*Streptomyces bungoensis* DSM 41781^T (99.62)	-	-	-	-	-	-	-	-	9	10	-	-
H21 (MW724540)	*Streptomyces hyaluromycini* NBRC 110483^T (99.87)	-	-	-	-	-	-	-	-	-	-	-	-
H24 (MW724541)	*Streptomyces bauhiniae* Bv016^T (100.00)	-	-	-	-	-	-	-	-	-	-	-	-
H30 (MW724542)	*Streptomyces corchorusii* DSM 40340^T (99.02)	-	-	-	-	-	-	-	-	-	8	-	-
H37 (MW724543)	*Streptomyces thermoviolaceus subsp. Thermoviolaceus* DSM 40443^T (98.50)	16	17	-	-	10	11	13	14	14	17	-	-
H38 (MW724544)	*Streptomyces thermocarboxydus* DSM 44293^T (99.88)	-	-	-	-	-	-	-	-	-	-	-	-
H40 (MW724545)	*Rhodococcus ruber* DSM 43338^T (99.01)	-	-	-	-	-	-	-	-	-	-	-	-
H41 (MW724546)	*Streptomyces rubrogriseus* LMG 20318^T (99.26)	-	-	-	-	-	-	-	-	-	-	-	-
H42 (MW724547)	*Streptomyces bungoensis* DSM 41781^T (99.64)	-	-	-	-	-	-	-	-	-	-	-	-
H47 (MW724548)	*Streptomyces rubidus* 13C15^T (99.14)	-	-	-	-	-	-	-	-	-	-	-	-
H49 (MW724549)	*Micromonospora profundi* DS3010^T (99.75)	-	-	-	-	-	-	-	-	-	-	-	-
H51 (MW724550)	*Streptomyces griseochromogenes* ATCC 14511^T (99.38)	-	-	-	-	-	-	-	-	19	23	26	26
H52 (MW724551)	*Streptacidiphilus bronchialis* DSM 106435^T (98.75)	-	-	-	-	-	-	-	-	7	-	-	-
H55 (MW724552)	*Streptomyces galbus* DSM 40089^T (99.50)	-	-	-	-	-	-	-	-	20	24	22	19
H59 (MW724553)	*Streptomyces hawaiiensis* NBRC 12784^T (99.76)	-	-	-	-	-	-	-	-	7	9	-	-
H65 (MW724554)	*Streptomyces griseoincarnatus* LMG 19316^T (100.00)	-	-	-	-	10	-	-	-	10	18	-	-
H93 (MW724555)	*Micromonospora avicenniae* DSM 45758^T (99.75)	-	-	-	-	-	-	-	-	-	-	-	-
H112 (MW724556)	*Gordonia polyisoprenivorans* NBRC 16320^T (98.54)	-	-	-	-	-	-	-	-	9	10	-	-
H113 (MW724557)	*Streptomyces phaeoluteigriseus* DSM 41896^T (100.00)	-	-	-	-	-	-	-	-	-	-	-	-
H115 (MW724558)	*Streptomyces glauciniger* CGMCC 4.1858^T (99.60)	-	-	-	-	-	-	-	-	-	-	-	-

H121 (MW724559)	*Rhodococcus zopfii* NBRC 100606^T (100.00)	-	-	-	-	-	-	-	-	9	10	17	-
H123 (MW724560)	*Streptomyces roseicoloratus* TRM 44457^T (99.87)	-	-	-	-	-	-	-	-	-	-	-	-
H124 (MW724561)	*Streptomyces angustmyceticus* NRRL B-2347^T (100.00)	-	-	-	-	-	-	-	7	-	-	-	-
H127 (MW724562)	*Streptomyces kebangsaanensis* SUK12^T (99.50)	-	-	-	-	-	-	-	-	-	-	-	-
H132 (MW724563)	*Micromonospora pisi* DSM 45175^T (97.89)	-	-	-	-	-	-	-	-	-	-	-	-
H133 (MW724564)	*Streptomyces carpaticus* NBRC 15390^T (99.75)	-	-	-	-	-	-	-	-	-	-	-	-
H137 (MW724565)	*Streptomyces naganishii* NBRC 12892^T (99.01)	-	-	-	-	-	-	-	-	-	-	-	-
H138 (MW724566)	*Streptomyces lannensis* TA4-8^T (100.00)	-	-	-	-	-	-	-	-	-	-	-	-
H140 (MW724567)	*Streptomyces youssoufiensis* X4^T (99.50)	-	-	-	-	-	-	-	-	7	9	17	-
M3 (MW724568)	*Mycolicibacterium porcinum* CIP 10539^T (100.00)	-	-	-	-	-	-	-	-	-	-	-	-
M4 (MW724569)	*Gordonia alkanivorans* NBRC 16433^T (100.00)	-	-	-	-	-	-	-	-	-	-	-	-
M5 (MW724570)	*Isoptericola jiangsuensis* DSM 21863^T (99.74)	-	-	-	-	-	-	-	-	-	-	-	-
M7 (MW724571)	*Streptomyces fragilis* NRRL 2424^T (99.74)	-	-	-	-	-	-	-	-	-	-	-	-
M19 (MW724572)	*Kocuria flava* HO-9041^T (100.00)	-	-	-	-	-	-	-	-	-	-	-	-
M28 (MW724573)	*Streptomyces sundarbansensis* MS1/7^T (99.76)	-	-	-	-	-	-	-	-	12	11	10	19
M32 (MW724574)	*Brevibacterium linens* DSM 20425^T (99.09)	-	-	-	-	-	-	-	-	-	-	-	-
M33 (MW724575)	*Microbacterium oleivorans* NBRC 103075^T (98.37)	-	-	-	-	-	-	-	-	-	-	-	-
M36 (MW724576)	*Microbacterium radiodurans* GIMN1.002^T (98.18)	-	-	-	-	-	-	-	-	-	-	-	-
M37 (MW724577)	*Microbacterium saccharophilum* K-1^T (98.91)	-	-	-	-	-	-	-	-	-	-	-	-
M49 (MW724578)	*Microbacterium paraoxydans* NBRC 103076^T (99.74)	-	-	-	-	-	-	-	-	-	-	-	-
M51 (MW724579)	*Rhodococcus pyridinivorans* DSM44555^T (100.00)	-	-	-	-	-	-	-	-	-	-	-	-
M64 (MW724580)	*Isoptericola dokdonensis* DS-3^T (100.00)	-	-	-	-	-	-	-	-	-	7	-	-
M70 (MW724581)	*Brevibacterium celere* KMM 3637^T (99.74)	-	-	-	-	-	-	-	-	-	-	-	-
M72 (MW724582)	*Microbacterium hydrothermale* 0704C9-2^T (99.02)	-	-	-	-	-	-	-	-	-	-	-	-
M73 (MW724583)	*Rhodococcus hoagii* DSM 20295^T (100.00)	-	-	-	-	-	-	-	-	-	-	-	-
M74 (MW724584)	*Corynebacterium variabile* DSM 20132^T (99.36)	-	-	-	-	-	-	-	-	-	-	-	-

M76 (MW724585)	*Janibacter indicus* CGMCC 1.12511^{T} (99.88)	-	-	-	-	-	-	-	-	-	-	-	-
M81 (MW724586)	*Citricoccus alkalitolerans* YIM 70010^{T} (99.63)	-	-	-	-	-	-	-	-	-	-	-	-
M84 (MW724587)	*Micromonospora auratinigra* DSM 44815^{T} (99.07)	-	-	-	-	-	-	-	-	-	-	-	-
M86 (MW724588)	*Aeromicrobium tamlense* SSW1-57^{T} (99.87)	-	-	-	-	-	-	-	-	-	-	-	-
M90 (MW724589)	*Kocuria dechangensis* NEAU-ST5-33^{T} (99.09)	-	-	-	-	-	-	-	-	-	-	-	-
M91 (MW724590)	*Brevibacterium permense* VKM AC-2280^{T} (99.87)	-	-	-	-	-	-	-	-	-	-	-	-
M102 (MW724591)	*Arthrobacter gandavensis* R812^{T} (99.60)	-	-	-	-	-	-	-	-	-	-	-	-
M104 (MW724592)	*Streptomyces griseoflavus* LMG 19344^{T} (99.75)	-	-	-	-	-	-	-	-	-	-	-	-
M106 (MW724593)	*Streptomyces geysiriensis* NBRC 15413^{T} (99.75)	14	9	7	-	16	-	10	8	14	20	11	13
M107 (MW724594)	*Streptomyces andamanensis* KC-112^{T} (99.75)	-	-	-	-	-	-	-	-	7	7	-	-
M108 (MW724595)	*Phycicoccus endophyticus* IP6SC6^{T} (98.61)	-	-	-	-	-	-	-	-	-	-	-	-
M110 (MW724596)	*Agromyces tropicus* CM9-9^{T} (99.88)	-	-	-	-	-	-	-	-	-	-	-	-
M111 (MW724597)	*Streptomyces smyrnaeus* SM3501^{T} (99.74)	-	-	-	-	9	-	-	-	13	16	17	15
M112 (MW724598)	*Micromonospora globispora* S2901^{T} (99.63)	-	-	-	-	-	-	-	-	-	-	-	-
M118 (MW724599)	*Microbacterium hominis* NBRC 15708^{T} (98.91)	-	-	-	-	-	-	-	-	-	-	-	-
M129 (MW724600)	*Streptomyces nanshensis* SCSIO 01066^{T} (99.63)	-	-	-	-	-	-	-	-	-	-	-	-
M131 (MW724601)	*Cellulosimicrobium marinum* RS-7-4^{T} (100.00)	-	-	-	-	-	-	-	-	-	-	-	-
M133 (MW724602)	*Blastococcus aggregatus* DSM 4725^{T} (99.61)	-	-	-	-	-	-	-	-	-	-	-	-
Y2 (MW724603)	*Streptomyces hygroscopicus subsp. hygroscopicus* NBRC 13472^{T} (99.63)	-	-	-	-	-	-	-	-	16	21	18	23
Y3 (MW724604)	*Streptomyces aurantiogriseus* NBRC 12842^{T} (99.40)	-	-	-	-	-	-	-	-	7	8	-	-
Y4 (MW724605)	*Streptomyces albogriseolus* NRRL B-1305^{T} (100.00)	12	13	-	-	17	10	10	9	17	26	-	16
Y8 (MW724606)	*Streptomyces pseudogriseolus* NRRL B-3288^{T} (100.00)	-	-	-	-	-	-	-	-	7	-	11	-
Y9 (MW724607)	*Streptomyces pluripotens* MUSC 135^{T} (99.87)	14	17	7	8	16	-	17	-	16	23	-	-
Y13 (MW724608)	*Streptomyces qinglanensis* 172205^{T} (100.00)	-	-	-	-	-	-	-	-	8	8	14	-
Y14 (MW724609)	*Streptomyces coelicoflavus* NBRC 15399^{T} (100.00)	-	-	-	-	-	-	-	-	-	-	10	-
Y15 (MW724610)	*Streptomyces cellulosae* NBRC 13027^{T} (100.00)	-	-	-	-	-	-	-	-	8	11	-	13

Y20 (MW724611)	*Streptomyces levis* NBRC 15423^T (99.75)	-	-	-	-	-	-	-	-	15	18	8	19
Y25 (MW724612)	*Streptomyces hydrogenans* NBRC 13475^T (100.00)	-	-	-	-	-	-	-	-	-	-	-	-
Y26 (MW724613)	*Streptomyces althioticus* NRRL B-3981^T (99.50)	-	-	-	-	-	-	-	-	-	-	-	-
Y27 (MW724614)	*Streptomyces parvulus* NBRC 13193^T (99.75)	-	-	-	-	-	-	-	-	-	-	-	-
Y36 (MW724615)	*Serinibacter salmoneus* DSM 21801^T (100.00)	-	-	-	-	-	-	-	-	-	-	9	-
Y46 (MW724616)	*Streptomyces antibioticus* NBRC 12838^T (100.00)	-	-	-	-	-	-	9	9	19	25	17	24
Y47 (MW724617)	*Streptomyces viridochromogenes* NBRC 3113^T (99.75)	-	-	-	-	-	-	-	-	-	-	-	-
Y50 (MW724618)	*Micromonospora saelicesensis* Lupac 09^T (99.36)	-	-	-	-	-	-	-	-	-	-	-	-
Y68 (MW724619)	*Micromonospora chaiyaphumensis* DSM 45246^T (99.45)	-	-	-	-	-	-	-	-	-	-	-	-
Y84 (MW724620)	*Streptomyces pseudovenezuelae* DSM 40212^T (99.23)	-	-	-	-	-	-	-	-	7	7	-	9
Y87 (MW724621)	*Micromonospora andamanensis* SP03-05^T (100.00)	-	-	-	-	-	-	-	-	-	7	-	-
Y95 (MW724622)	*Micromonospora carbonacea* DSM 43168^T (99.88)	-	-	-	-	-	-	-	-	-	-	-	-
Y100 (MW724623)	*Micromonospora maritima* D10-9-5^T (99.61)	-	-	-	-	-	-	-	-	-	-	-	-
Y101 (MW724624)	*Micromonospora humi* DSM 45647^T (99.61)	-	-	-	-	-	-	-	-	7	10	-	9
Y121 (MW724625)	*Streptomyces tendae* ATCC 19812^T (99.87)	-	-	-	-	-	-	-	-	-	-	-	-
Y128 (MW724626)	*Micromonospora maritima* D10-9-5^T (99.75)	-	-	-	-	-	-	-	-	-	-	-	-
Y129 (MW724627)	*Streptomyces cacaoi subsp. cacaoi* NRRL B-1220^T (99.88)	-	-	-	-	13	-	-	-	-	-	-	-
Y135 (MW724628)	*Streptomyces malachitofuscus* NBRC 13059^T (99.40)	-	-	-	-	-	-	-	-	-	-	-	-
Y150 (MW724629)	*Micromonospora narathiwatensis* DSM 45248^T (100.00)	-	-	-	-	-	-	-	-	-	-	-	-
Y168 (MW724630)	*Streptomyces sparsus* YIM 90018^T (99.88)	-	-	-	-	-	-	-	-	-	-	-	-
Y170 (MW724631)	*Streptomyces carpinensis* NRRL B-16921^T (98.88)	-	-	-	-	-	-	-	-	-	-	-	-
Y172 (MW724632)	*Salinibacterium hongtaonis* 194^T (97.97)	-	-	-	-	-	-	-	-	-	-	-	-
Y179 (MW724633)	*Motilibacter rhizosphaerae* RS-16^T (96.83)	-	-	-	-	-	-	-	-	-	-	-	-
Y198 (MW724634)	*Micromonospora pallida* DSM 43817^T (99.13)	-	-	-	-	-	-	-	-	10	14	8	8
Y199 (MW724635)	*Mycobacterium grossiae* SCH^T (99.12)	-	-	-	-	-	-	-	-	-	-	-	-
H11 (MW724636)	*Kocuria palustris* DSM 11925^T (100.00)	-	-	-	-	-	-	-	-	-	-	-	-

H12 (MW724637)	*Streptomyces similanensis* KC-106^T (99.13)	-	-	-	-	-	-	10	9	20	23	12	27
H18 (MW724638)	*Streptomyces seoulensis* NRRL B-24310^T (100.00)	-	7	-	-	-	-	8	7	17	20	19	23
H25 (MW724639)	*Citricoccus zhacaiensis* FS24^T (98.62)	-	-	-	-	-	-	-	-	-	-	-	-
H28 (MW724640)	*Intrasporangium oryzae* NRRL B-24470^T (98.36)	-	-	-	-	-	-	-	-	-	-	-	-
H29 (MW724641)	*Janibacter hoylei* DSM 21601^T (99.00)	-	-	-	-	-	-	-	-	-	-	-	-
H36 (MW724642)	*Gulosibacter faecalis* ATCC 13722^T (99.49)	-	-	-	-	-	-	-	-	-	-	-	-
H43 (MW724643)	*Sinomonas flava* CW 108^T (99.76)	-	-	-	-	-	-	-	-	-	-	-	-
H58 (MW724644)	*Intrasporangium flavum* MUSC 78^T (99.01)	-	-	-	-	-	-	-	-	-	-	-	-
H64 (MW724645)	*Corynebacterium xerosis* ATCC 373^T (100.00)	-	-	-	-	-	-	-	-	-	-	-	-
H81 (MW724646)	*Corynebacterium xerosis* ATCC 373^T (99.22)	-	-	-	-	-	-	-	-	-	-	-	-
H85 (MW724647)	*Microbacterium esteraromaticum* DSM 8609^T (99.76)	-	-	-	-	-	-	-	-	-	-	-	-
H86 (MW724648)	*Nocardia grenadensis* NBRC 108939^T (100.00)	-	-	-	-	-	-	-	-	-	-	-	-
H88 (MW724649)	*Janibacter alkaliphilus* SCSIO 10480^T (98.76)	-	-	-	-	-	-	-	-	-	-	-	-
H90 (MW724650)	*Brevibacterium luteolum* CF87^T (99.45)	-	-	-	-	-	-	-	-	7	7	-	-
H96 (MW724651)	*Gordonia terrae* NBRC 100016^T (100.00)	-	-	-	-	-	-	-	-	-	-	-	-
H97 (MW724652)	*Mycolicibacterium pallens* CZH-8^T (100.00)	-	-	-	-	-	-	-	-	-	-	-	-
H100 (MW724653)	*Aeromicrobium massiliense* JC14^T (99.40)	-	-	-	-	-	-	-	-	-	-	-	-
H117 (MW724654)	*Mycolicibacterium anyangense* QIA-38^T (100.00)	-	-	-	-	-	-	-	-	-	-	-	-
H120 (MW724655)	*Nocardia niigatensis* NBRC 100131^T (100.00)	-	-	-	-	-	-	-	-	-	-	-	-
H149 (MW724656)	*Actinomadura glauciflava* AS 4.1202^T (99.74)	-	-	-	-	-	-	-	-	13	-	-	-
H150 (MW724657)	*Agrococcus terreus* DNG5^T (99.88)	-	-	-	-	-	-	-	-	-	-	-	-
H156 (MW724658)	*Arthrobacter ruber* MDB1-42^T (98.98)	-	-	-	-	-	-	-	-	-	-	-	-
H157 (MW724659)	*Micrococcus luteus* NCTC 2665^T (99.63)	-	-	-	-	-	-	-	-	7	-	-	-
M2 (MW724660)	*Glutamicibacter mysorens* LMG 16219^T (99.11)	-	-	-	-	-	-	-	-	-	-	-	-
M8 (MW724661)	*Streptomyces fradiae* DSM 40063^T (100.00)	-	-	-	-	-	-	-	-	-	-	-	-
M9 (MW724662)	*Micromonospora krabiensis* DSM 45344^T (100.00)	-	-	-	-	-	-	-	-	-	-	-	-

M18 (MW724663)	*Rhodococcus rhodochrous* NBRC 16069^{T} (99.88)	-	-	-	-	-	-	-	-	-	-	-	-
M22 (MW724664)	*Streptomyces shenzhenensis* 172115^{T} (99.88)	-	-	-	-	-	-	16	17	10	10	7	-
M25 (MW724665)	*Streptomyces coeruleoprunus* NBRC 15400^{T} (99.38)	-	-	-	-	-	-	-	-	-	-	-	-
M26 (MW724666)	*Micromonospora tulbaghiae* DSM 45142^{T} (100.00)	-	-	-	-	-	-	-	-	-	-	-	-
M27 (MW724667)	*Georgenia muralis* DSM 14418^{T} (99.87)	-	-	-	-	-	-	-	-	8	8	8	-
M30 (MW724668)	*Micromonospora echinofusca* DSM 43913^{T} (99.75)	-	-	-	-	-	-	-	-	8	-	9	-
M35 (MW724669)	*Glutamicibacter soli* SYB2^{T} (99.87)	-	-	-	-	-	-	-	-	-	-	-	-
M40 (MW724670)	*Streptomyces misionensis* DSM 40306^{T} (99.17)	-	-	-	-	-	-	-	-	-	-	-	-
M45 (MW724671)	*Demequina salsinemoris* NBRC 105323^{T} (99.63)	-	-	-	-	-	-	-	-	14	17	14	17
M61 (MW724672)	*Cellulosimicrobium funkei* ATCC BAA-886^{T} (99.51)	-	-	-	-	-	-	-	-	-	-	-	-
M65 (MW724673)	*Streptomyces wuyuanensis* CGMCC 4.7042^{T} (100.00)	-	-	-	-	-	-	-	-	-	-	-	-
M69 (MW724674)	*Jonesia denitrificans* DSM 20603^{T} (99.87)	-	-	-	-	-	-	-	-	-	-	-	-
M71 (MW724675)	*Cellulosimicrobium cellulans* LMG 16121^{T} (99.74)	-	-	-	-	-	-	-	-	-	-	-	-
Y24 (MW724676)	*Kocuria rosea* DSM 20447^{T} (99.34)	-	-	-	-	-	-	-	-	-	-	-	-
Y32 (MW724677)	*Micrococcus endophyticus* YIM 56238^{T} (99.74)	-	-	-	-	-	-	-	-	-	-	-	-
Y33 (MW724678)	*Microbacterium hibisci* THG-T2.14^{T} (98.98)	-	-	-	-	-	-	-	-	-	-	-	-
Y35 (MW724535)	*Microbacterium testaceum* NBRC 12675^{T} (98.47)	-	-	-	-	-	-	-	-	-	-	-	-
Y43 (MW724679)	*Agrococcus carbonis* DSM 22965^{T} (99.50)	-	-	-	-	-	-	-	-	-	-	-	-
Y56 (MW724680)	*Kineococcus mangrovi* L2-1-L1^{T} (99.50)	-	-	-	-	-	-	-	-	-	-	-	-
Y67 (MW724681)	*Mumia xiangluensis* NEAU-KD1^{T} (98.81)	-	-	-	-	-	-	-	-	-	-	-	-
Y78 (MW724682)	*Agromyces binzhouensis* OACT353^{T} (99.75)	-	-	-	-	-	-	-	-	-	-	-	-
Y80 (MW724683)	*Micrococcus terreus* CGMCC 1.7054^{T} (100.00)	-	-	-	-	-	-	-	-	10	-	-	-
Y97 (MW724684)	*Brachybacterium squillarum* M-6-3^{T} (99.11)	-	-	-	-	-	-	-	-	-	-	-	-
Y106 (MW724685)	*Microbacterium fluvii* YSL3-15^{T} (98.39)	-	-	-	-	-	-	-	-	-	-	-	-
Y108 (MW724686)	*Mycolicibacterium confluentis* DSM 44017^{T} (100.00)	-	-	-	-	-	-	-	-	-	-	-	-
Y111 (MW724687)	*Isoptericola chiayiensis* 06182M-1^{T} (99.00)	-	-	-	-	-	-	-	-	-	-	-	-

Y112 (MW724688)	*Mycolicibacterium poriferae* ATCC 35087^T (99.74)	–	–	–	–	–	–	–	–	–	–	–	–
Y119 (MW724689)	*Cellulosimicrobium funkei* ATCC BAA-886^T (99.88)	–	–	–	–	–	–	–	–	–	–	–	–
Y122 (MW724690)	*Arthrobacter gandavensis* R 5812^T (99.65)	–	–	–	–	–	–	–	–	–	–	–	–
Y124 (MW724691)	*Leucobacter chromiiresistens* JG 31^T (99.88)	–	–	–	–	–	–	–	–	–	–	–	–
Y126 (MW724692)	*Micrococcus luteus* NCTC 2665^T (99.63)	-	-	-	-	-	-	-	-	7	7	-	-
Y130 (MW724693)	*Gordonia rubripertincta* NBRC 101908^T (99.74)	–	–	–	–	–	–	–	–	–	–	–	–
Y136 (MW724694)	*Brevibacterium senegalense* JC43^T (99.34)	–	–	–	–	–	–	–	–	–	–	–	–
Y143 (MW724695)	*Actinopolymorpha singaporensis* DSM 22024^T (99.38)	–	–	–	–	–	–	–	–	–	–	–	–
Y151 (MW724696)	*Mycolicibacterium sphagni* ATCC 33027^T (98.91)	–	–	–	–	–	–	–	–	–	–	–	–
Y153 (MW724697)	*Microbacterium fluvii* YSL3-15^T (99.25)	–	–	–	–	–	–	–	–	–	–	–	–
Y154 (MW724698)	*Mycobacterium palauense* CECT 8779^T (99.89)	–	–	–	–	–	–	–	–	–	–	–	–
Y157 (MW724699)	*Micromonospora citrea* DSM 43903^T (99.50)	–	–	–	–	–	–	–	–	–	–	–	–
Y162 (MW724700)	*Agromyces indicus* NIO-1018^T (100.00)	–	–	–	–	–	–	–	–	–	–	–	–
Y165 (MW724701)	*Brachybacterium saurashtrense* JG 06^T (99.76)	–	–	–	–	–	–	–	–	–	–	–	–
Y171 (MW724702)	*Citricoccus nitrophenolicus* PNP1^T (99.63)	–	–	–	–	–	–	–	–	–	–	–	–
Y180 (MW724703)	*Serinibacter arcticus* K3-2^T (99.44)	–	–	–	–	–	–	–	–	–	–	–	–
Y185 (MW724704)	*Brachybacterium muris* C3H-21^T (99.62)	–	–	–	–	–	–	–	–	–	–	–	–
Y188 (MW724705)	*Aeromicrobium* halocynthiae KME 001^T (97.57)	–	–	–	–	–	–	–	–	–	–	–	–
Y192 (MW724706)	*Agrococcus jenensis* DSM 9580^T (99.50)	–	–	–	–	–	–	–	–	–	–	–	–
Y193 (MW724707)	*Brevibacterium sediminis* FXJ8.269^T (99.12)	–	–	–	–	–	–	–	–	–	–	–	–
Y194 (MW724708)	*Rhodococcus corynebacterioides* DSM 20151^T (100.00)	-	-	-	-	-	-	-	-	-	7	-	-
Y197 (MW724709)	*Microbacterium thalassium* IFO 16060^T (99.64)	-	-	-	-	-	-	-	-	7	7	-	-
M59 (MW724710)	*Microbacterium oleivorans* NBRC 103075^T (98.13)	–	–	–	–	–	–	–	–	–	–	–	–
M87 (MW724711)	*Brachybacterium squillarum* M-6-3^T (99.35)	–	–	–	–	–	–	–	–	–	–	–	–
M98 (MW724536)	*Serinibacter tropicus* PS-14-7^T (98.45)	–	–	–	–	–	–	–	–	–	–	–	–
M115 (MW724712)	*Phycicoccus endophyticus* IP6SC6^T (98.59)	–	–	–	–	–	–	–	–	–	–	–	–

M116 (MW724713)	*Serinicoccus profundi* MCCC 1A05965^T (98.69)	-	-	-	-	-	-	-	-	-	-	-	-
Methanol		-	-	-	-	-	-	-	-	-	-	-	-
Levofloxacin (10 µg)		36	39	26	19	40	24	28	23	24	15	19	-

Paper disk diameter, 6 mm; -, no Inhibitory zone.

Table S5. The trioxacarcin-type antibiotics isolated from actinomycetial strains.

Name	UVmax (nm)	Exact mass	Molecular Formula	Ref.
LL-D49194α1	230, 270, 399	992	$C_{48}H_{64}O_{22}$	[1,2]
LL-D49194β1	230, 270, 399	1010	$C_{48}H_{66}O_{23}$	[1,2]
LL-D49194β2	230, 270, 399	950	$C_{46}H_{62}O_{21}$	[1,2]
LL-D49194β3	230, 270, 399	806	$C_{39}H_{50}O_{18}$	[1]
LL-D49194γ	225, 270, 399(sh)	-	-	[1]
LL-D49194δ	230, 270, 399	-	-	[1]
LL-D49194ε	230, 270, 399	1052	$C_{50}H_{68}O_{24}$	[1]
LL-D49194ζ	230, 270, 399	-	-	[1]
LL-D49194η	230, 270, 399	848	$C_{41}H_{52}O_{19}$	[1]
LL-D49194ω1	230, 270, 399	968	$C_{46}H_{64}O_{22}$	[1]
LL-D49194ω2	230, 270, 399	-	-	[1]
LL-D49194ω3	230, 270, 399	866	$C_{41}H_{54}O_{20}$	[1]
Parimycin	260, 423, 447	396	$C_{22}H_{20}O_{7}$	[3]
trioxacarcin A	233, 271, 399	876	$C_{42}H_{52}O_{20}$	[4,5]
trioxacarcin B	233, 271, 399	894	$C_{42}H_{54}O_{21}$	[4,5]
trioxacarcin C	233, 271, 399	878	$C_{42}H_{54}O_{20}$	[4,5]
trioxacarcin D	270, 396	834	$C_{40}H_{50}O_{19}$	[5]
trioxacarcin E	-	740	$C_{34}H_{44}O_{18}$	[5]
trioxacarcin F	-	912	$C_{42}H_{56}O_{22}$	[5]
Gutingimycin	269, 399	1027	$C_{47}H_{57}N_{5}O_{21}$	[5,6]
DC-45-A1	-	704	$C_{34}H_{40}O_{16}$	[7-9]
DC-45-A2	-	518	$C_{25}H_{26}O_{12}$	[7-9]

-: no data

Table S6. The information of mangrove-derived soil samples in different sites of Leizhou Peninsula, China.

**Samples**	**Sampling sites**	**The characteristic of soil**	**Longitude (E)**	**Latitude (N)**	**Sampling depth**
Sample 1	He'an Town, Xuwen County	Rhizosphere soil of *Sonneratia apetala*	110.3708830	20.6395330	5-10 cm under surface
Sample 2	He'an Town, Xuwen County	Rhizosphere soil of *Sonneratia apetala*	110.3708830	20.6396330	5-10 cm under surface
Sample 3	He'an Town, Xuwen County	Rhizosphere soil of *Aegiceras corniculatum*	110.3709170	20.6395830	5-10 cm under surface
Sample 4	Dongsong Island, He'an Town, Xuwen County	Rhizosphere soil of *Avicennia marina*	110.3666000	20.6785170	5-10 cm under surface
Sample 5	Dongsong Island, He'an Town, Xuwen County	Rhizosphere soil of *Rhizophora stylosa*	110.3665170	20.6787170	5-10 cm under surface
Sample 6	Dongsong Island, He'an Town, Xuwen County	Rhizosphere soil of *Kandelia candel*	110.3665330	20.6787170	5-10 cm under surface
Sample 7	Dongsong Island, He'an Town, Xuwen County	Muddy soil of intertidal zone in mangrove	110.3674500	20.6799330	5-10 cm under surface
Sample 8	Dongsong Island, He'an Town, Xuwen County	Rhizosphere soil of *Sonneratia apetala*	110.3686330	20.6665330	5-10 cm under surface
Sample 9	Maichen Town, Xuwen County	Rhizosphere soil of *Rhizophora stylosa*	110.0107830	20.4205000	5-10 cm under surface
Sample 10	Maichen Town, Xuwen County	Rhizosphere soil of *Avicennia marina*	110.0107670	20.4205670	5-10 cm under surface
Sample 11	Techeng Island, Xiashan District	Rhizosphere soil of *Avicennia marina*	110.4404500	21.1553670	5-10 cm under surface
Sample 12	Techeng Island, Xiashan District	Rhizosphere soil of *Avicennia marina*	110.4403830	21.1554000	5-10 cm under surface
Sample 13	Techeng Island, Xiashan District	Rhizosphere soil of *Avicennia marina*	110.4413500	21.1574500	5-10 cm under surface

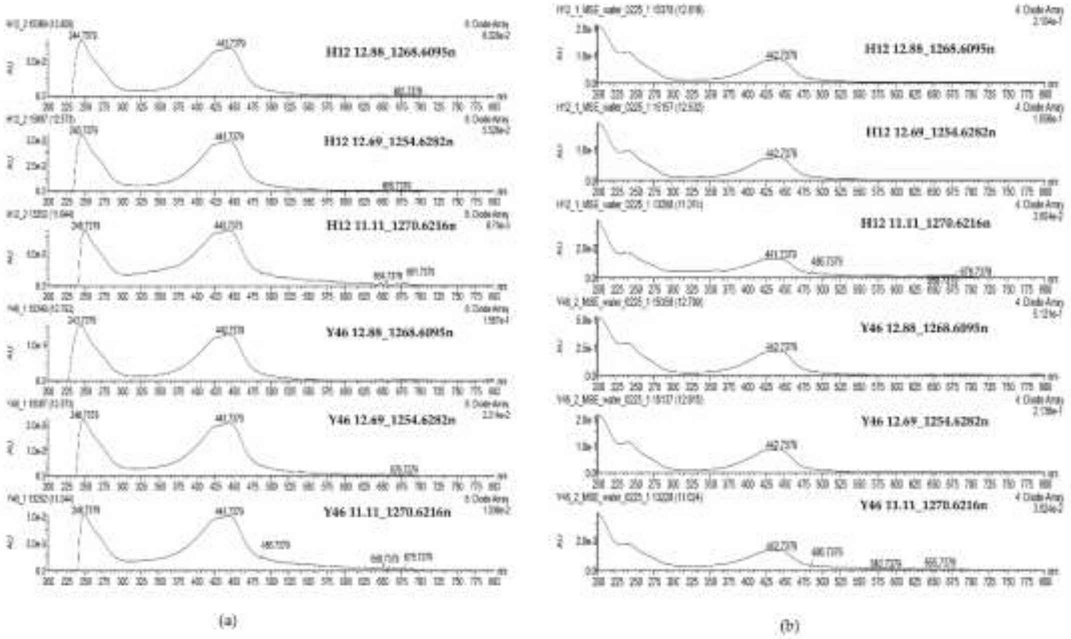

Figure S1. The UV spectra of three outliers (**1**-**3**) of samples Y46 and H12. (a) LC condition: ACN and water containing 0.1% HCOOH; (b) LC condition: ACN and water

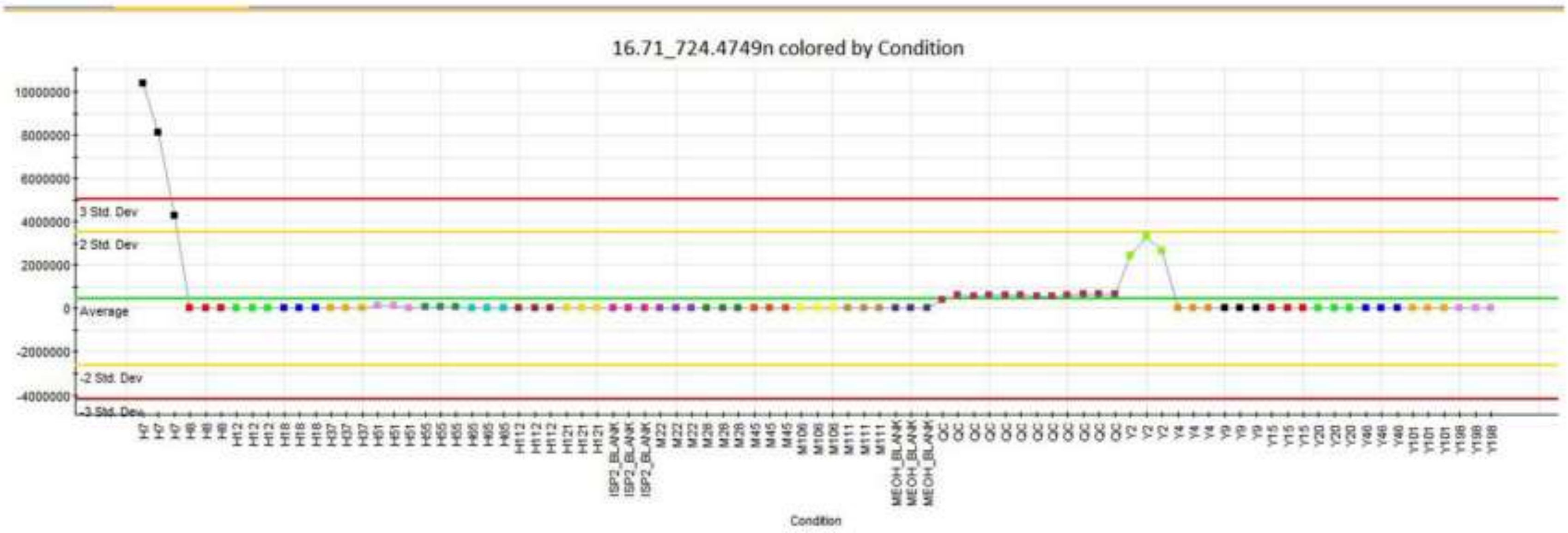

Figure S2. The feature statistic plot of 16.71_724.4749n (**4**) in all samples.

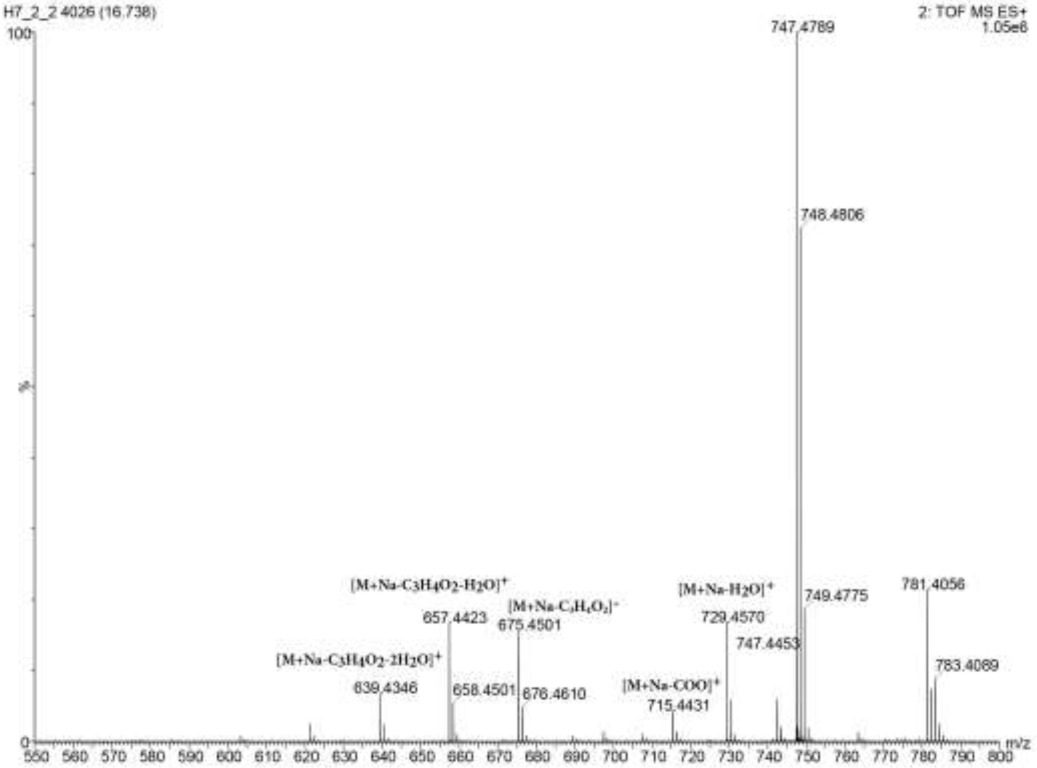

Figure S3. The MS/MS fragment pattern of the outlier 16.71_724.4749n (**4**) in sample H7.

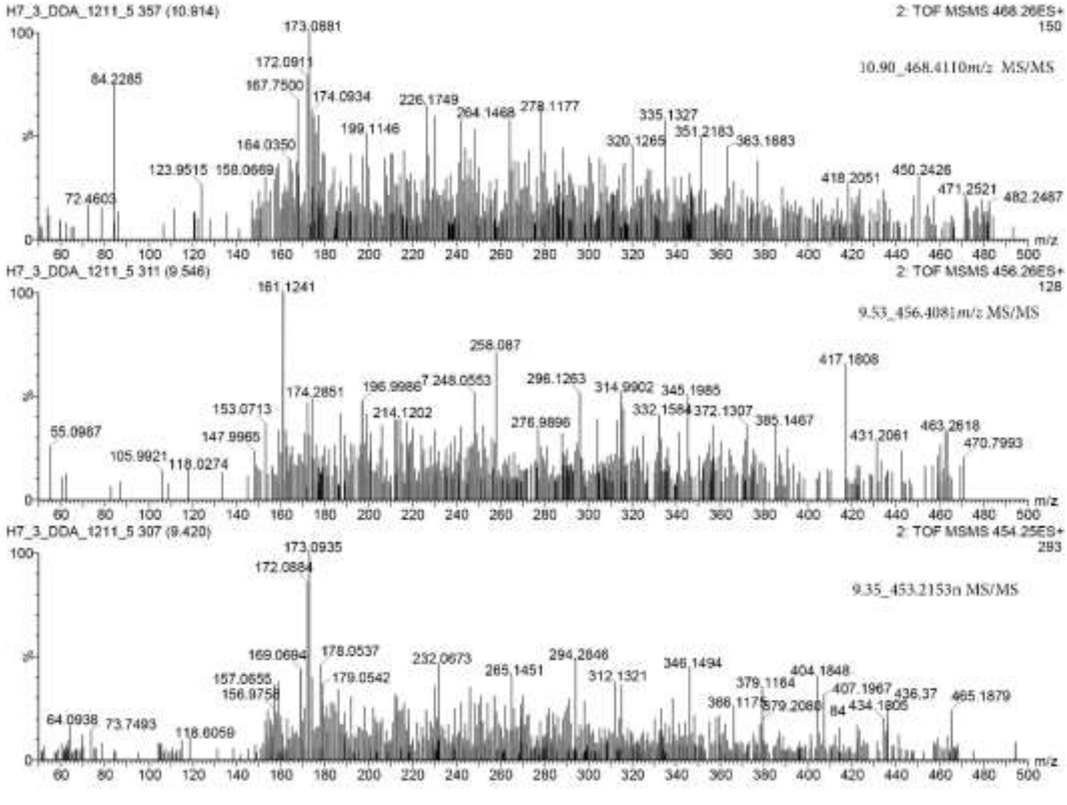

Figure S4. The MS/MS spectra of three false positive compounds in H7 acquired by DDA method.

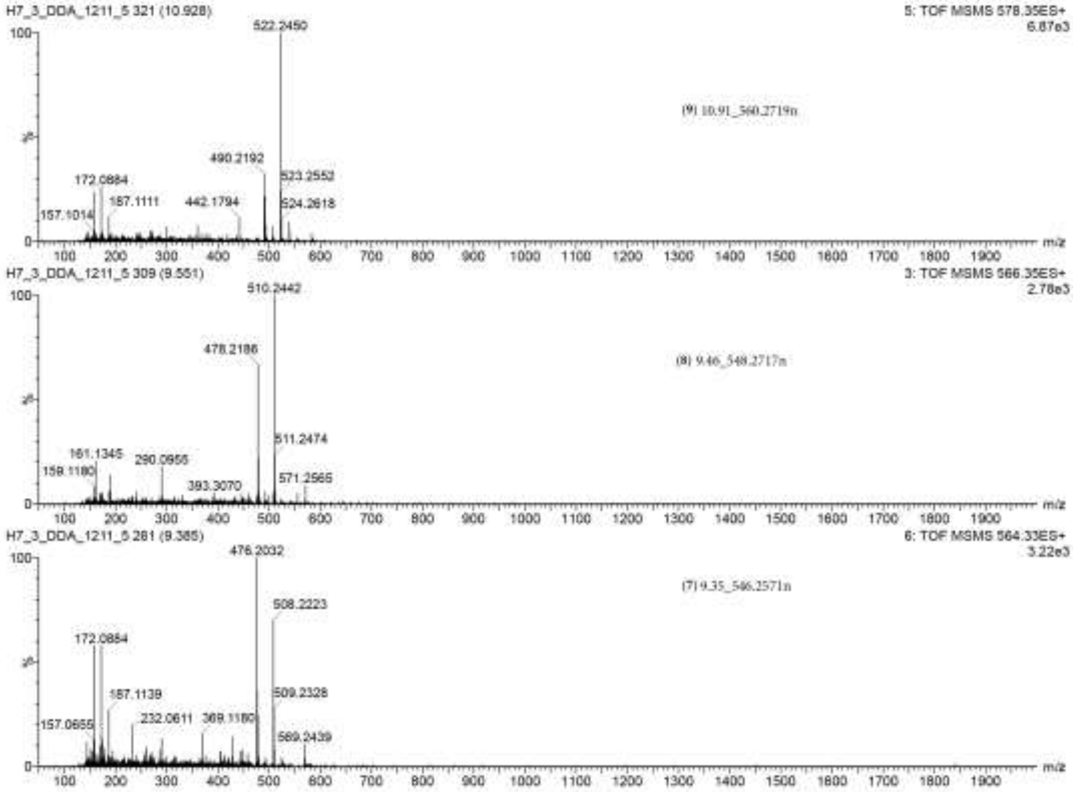

Figure S5. The MS/MS spectra of three revised compounds (**7-9**) in H7 acquired by DDA method.

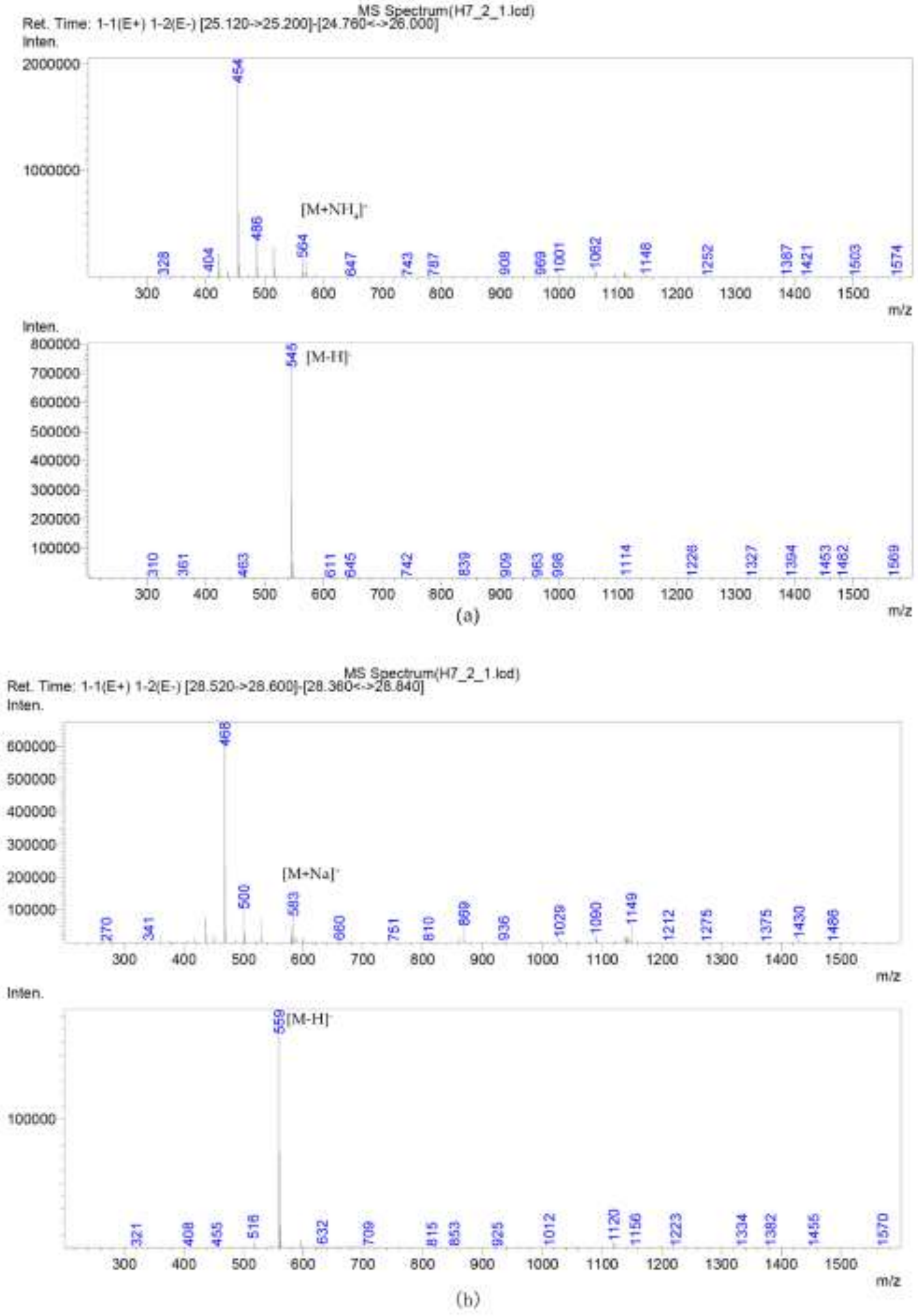

Figure S6. The positive and negative MS spectra of two revised compounds in the LC-MS of H7 (a: 9.35_546.2571n (**7**); b: 10.91_560.2719n (**9**)).

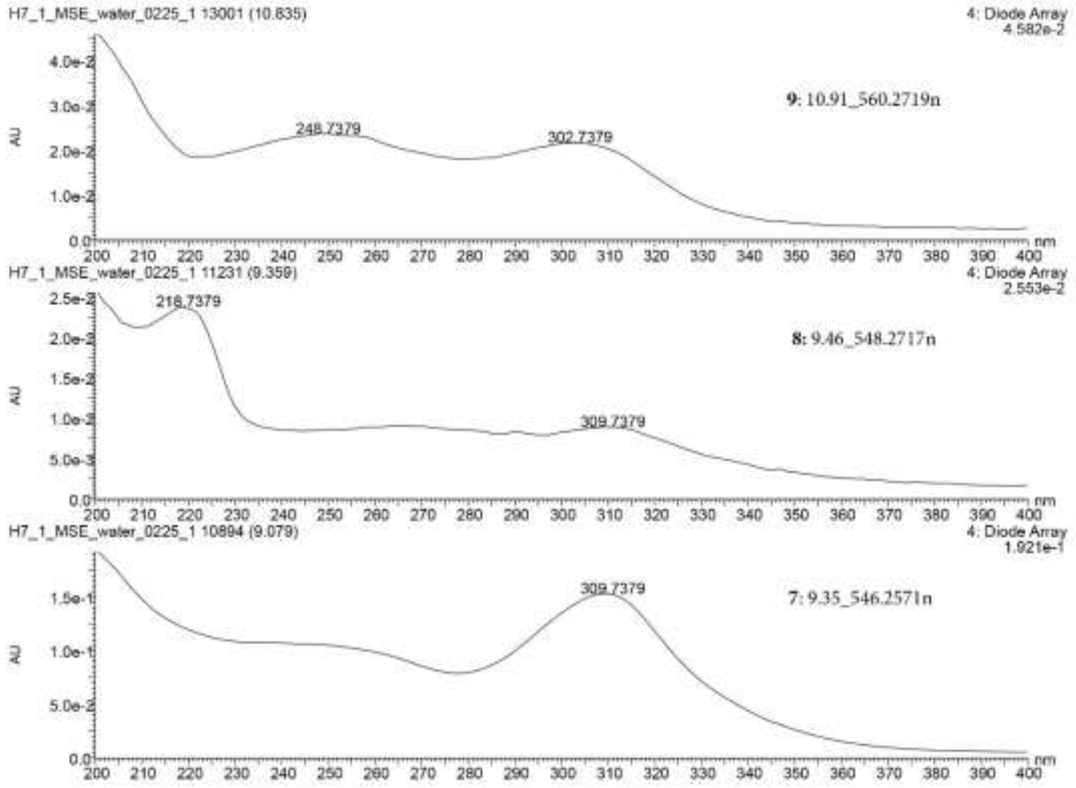

Figure S7. The UV spectra of three compounds **7-9** of the H7 eluting with ACN-H_2O (**7**: 9.35_546.2571n; **8**: 9.46_548.2717n; **9**: 10.91_560.2719n).

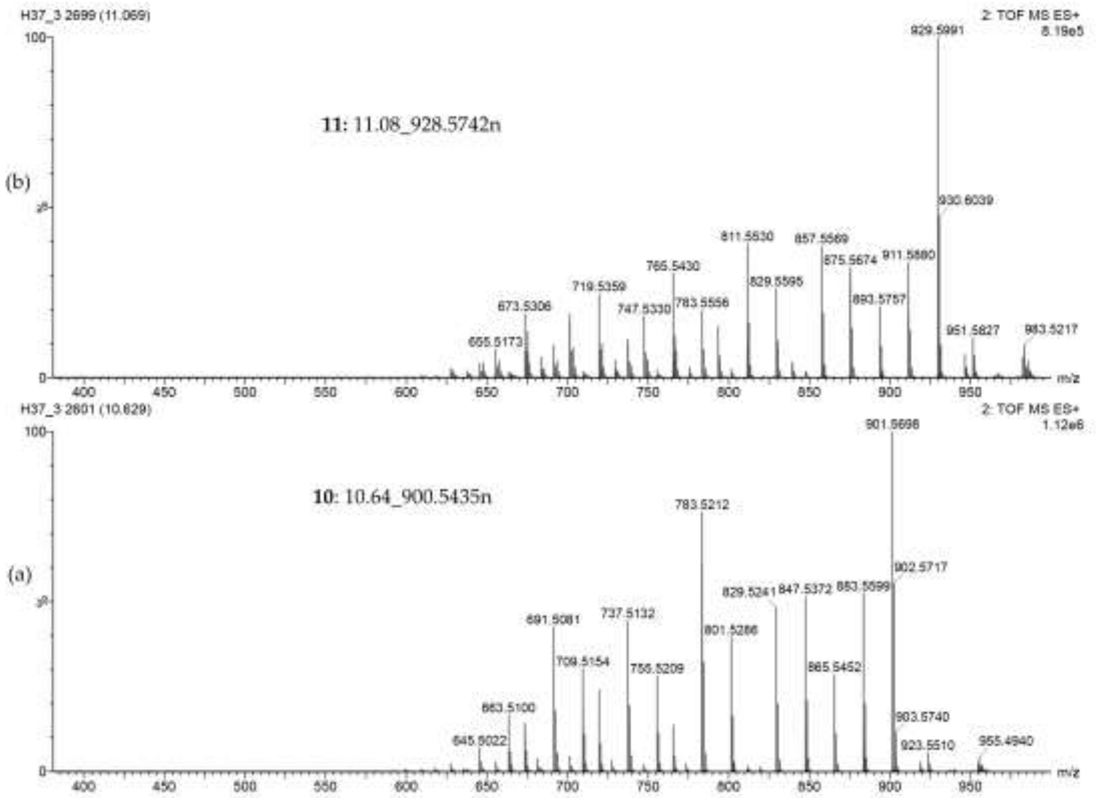

Figure S8. The MS/MS spectra of two compounds in H37 acquired by MSE method (a, 10.64_900.5435n (**10**); b, 11.08_928.5742n (**11**)).

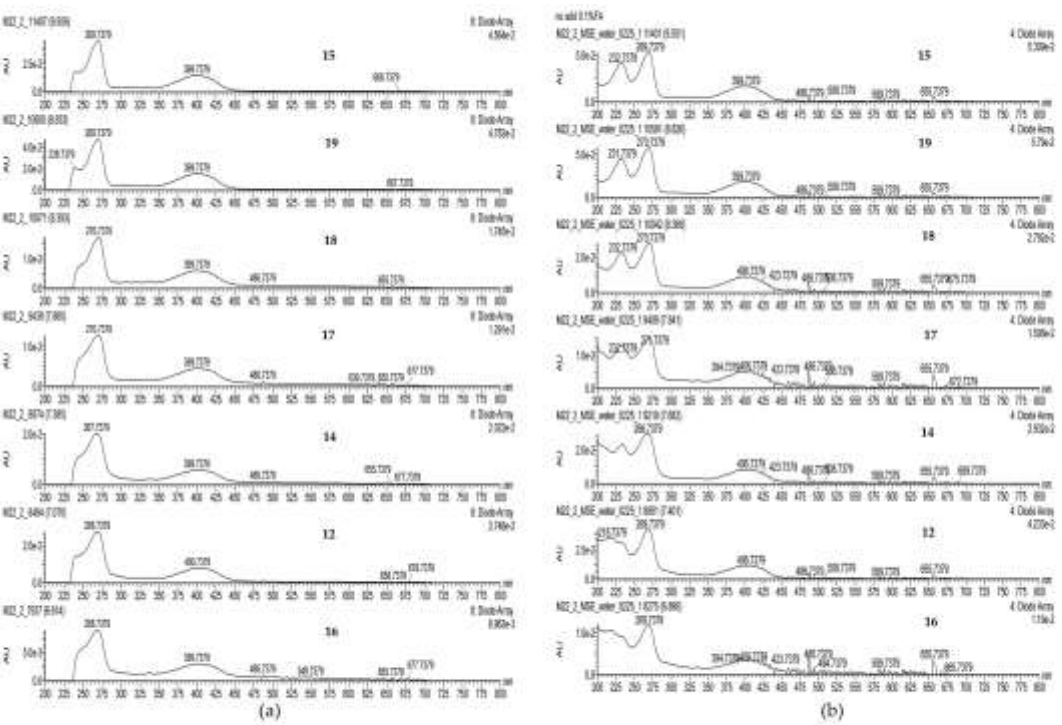

Figure S9. The UV spectra of seven trioxacarcin-type compounds in the UPLC-UV-HRMS chromatograms of M22. (a, LC conditions: ACN and H_2O containing with 0.1% HCOOH; b, LC conditions: ACN and H_2O; **12**, 7.16_1028.3600*m/z*; **14**, 7.47_1028.3592*m/z*; **15**, 9.55_876.2958n, trioxacarcin A; **16**, 6.69_1030.3751*m/z*; **17**, 7.94_1013.3486*m/z*; **18**, 8.43_894.3132n, trioxacarcin B; **19**, 8.89_878.3168n, trioxacarcin C).

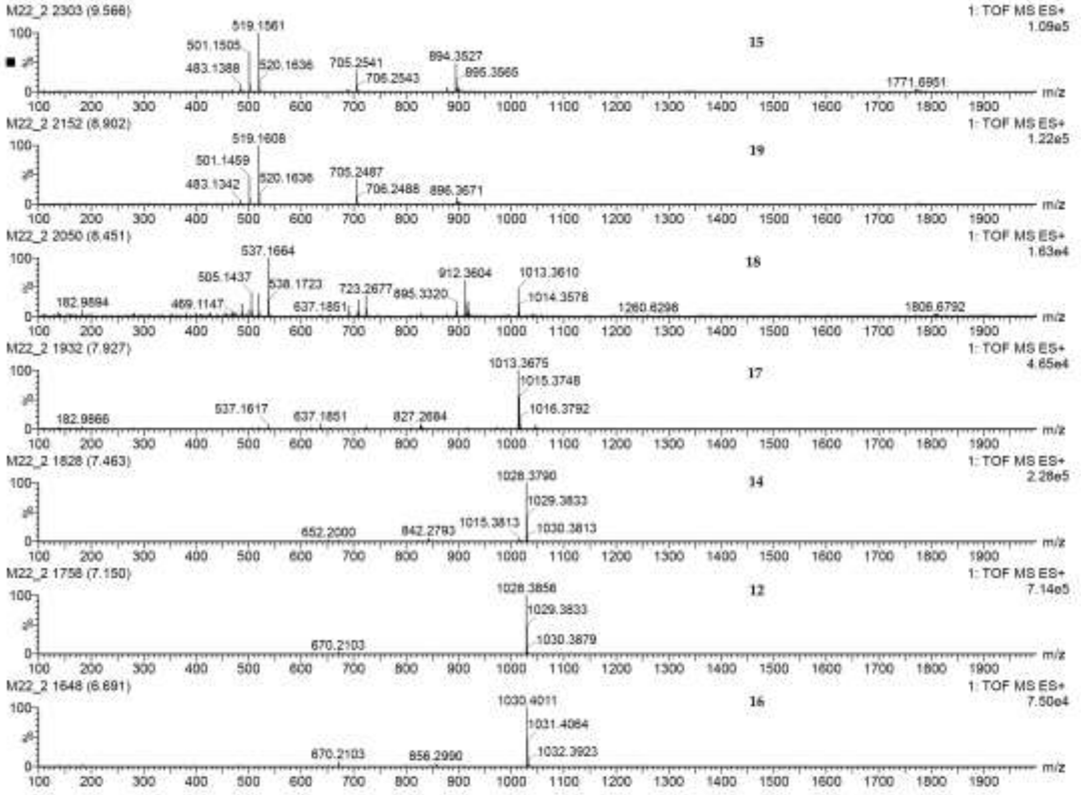

Figure S10. The MS spectra of seven trioxacarcin-type compounds in the UPLC-UV-HRMS chromatograms of M22. (**12**, 7.16_1028.3600*m/z*; **14**, 7.47_1028.3592*m/z*; **15**, 9.55_876.2958n, trioxacarcin A; **16**, 6.69_1030.3751*m/z*; **17**, 7.94_1013.3486*m/z*; **18**, 8.43_894.3132n, trioxacarcin B; **19**, 8.89_878.3168n, trioxacarcin C).

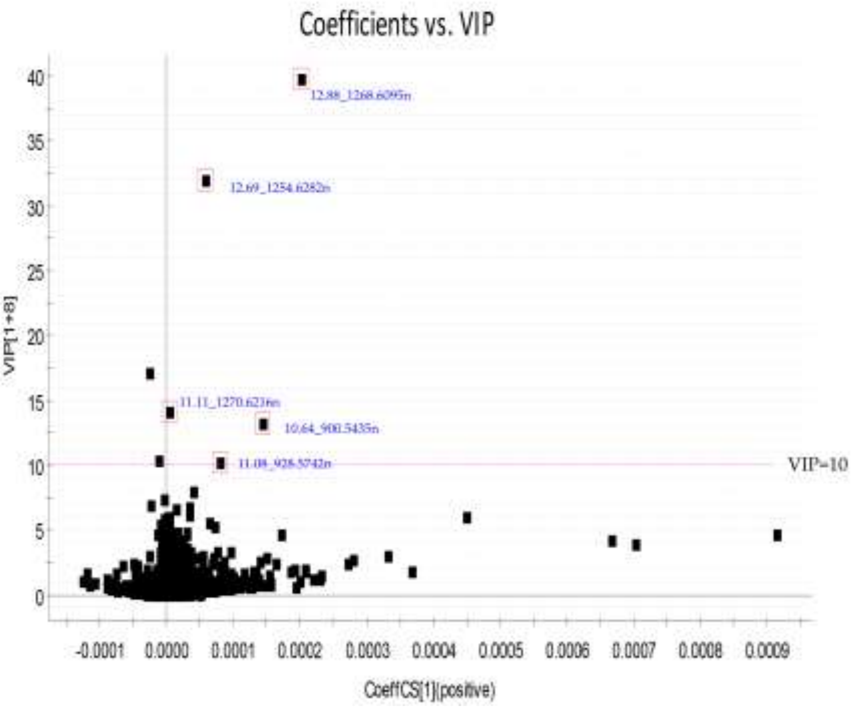

Figure S11. The VIP score of selected markers in the OPLS-DA model.

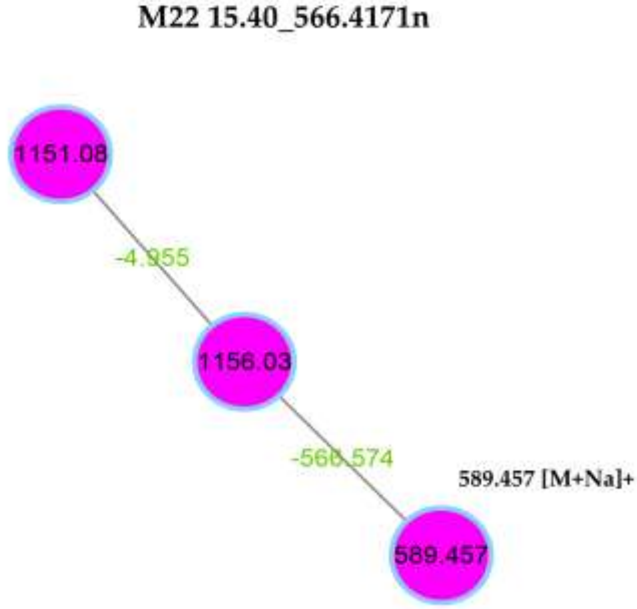

Figure S12. Molecular network of the cluster containing the compound 15.40_566.4171n (**13**) in M22 extract.

H37 10.64_900.5435n and 11.08_928.5742n

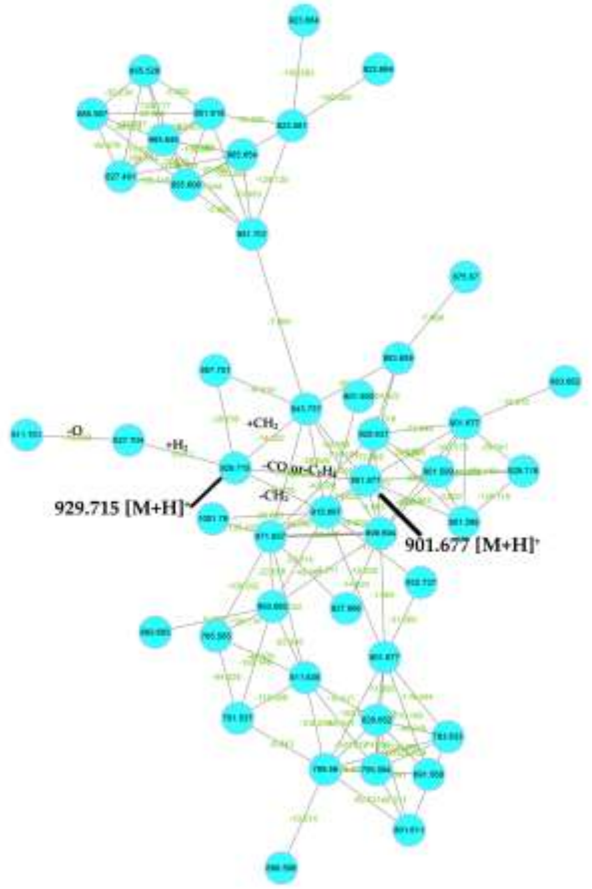

Figure S13. Molecular network of the cluster containing the compounds 10.64_900.5435n (**10**) and 11.08_928.5742n (**11**) in H37 extract.

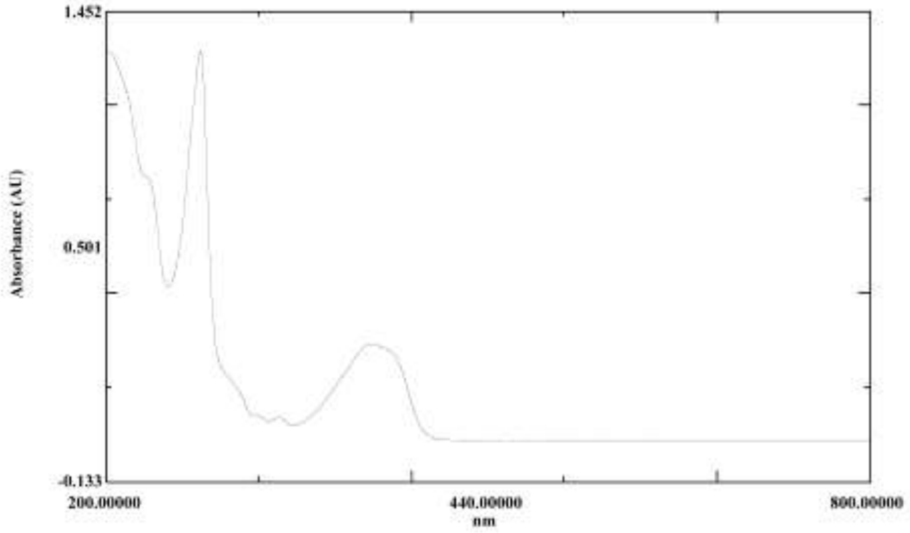

Figure S14. The UV spectrum of compound **16**

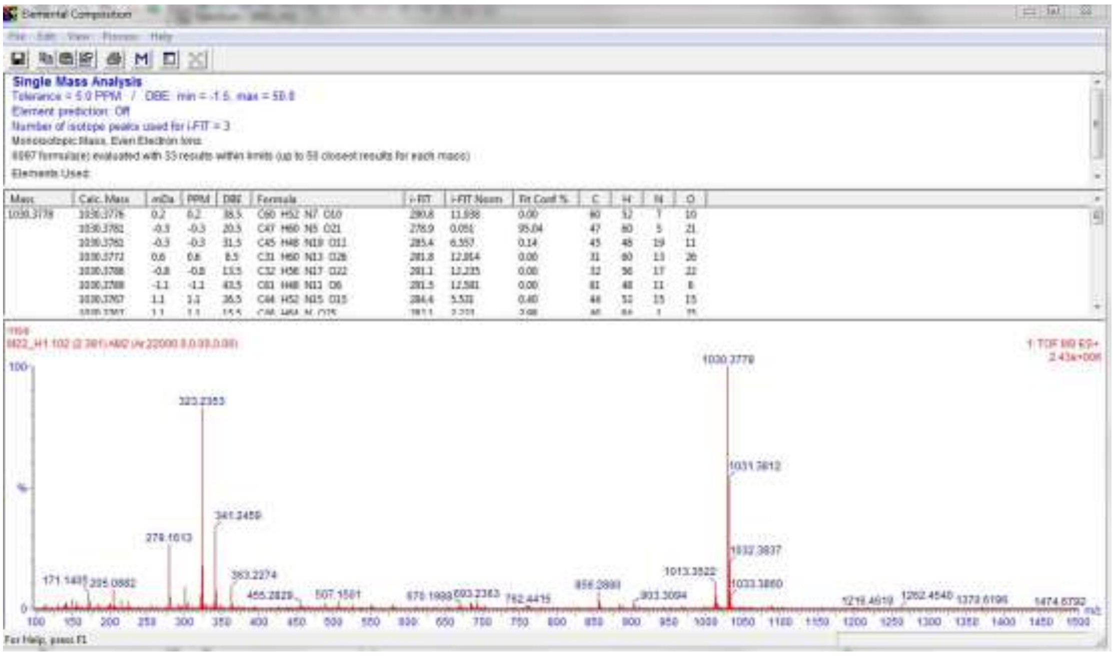

Figure S15. The HRESIMS of compound **16**

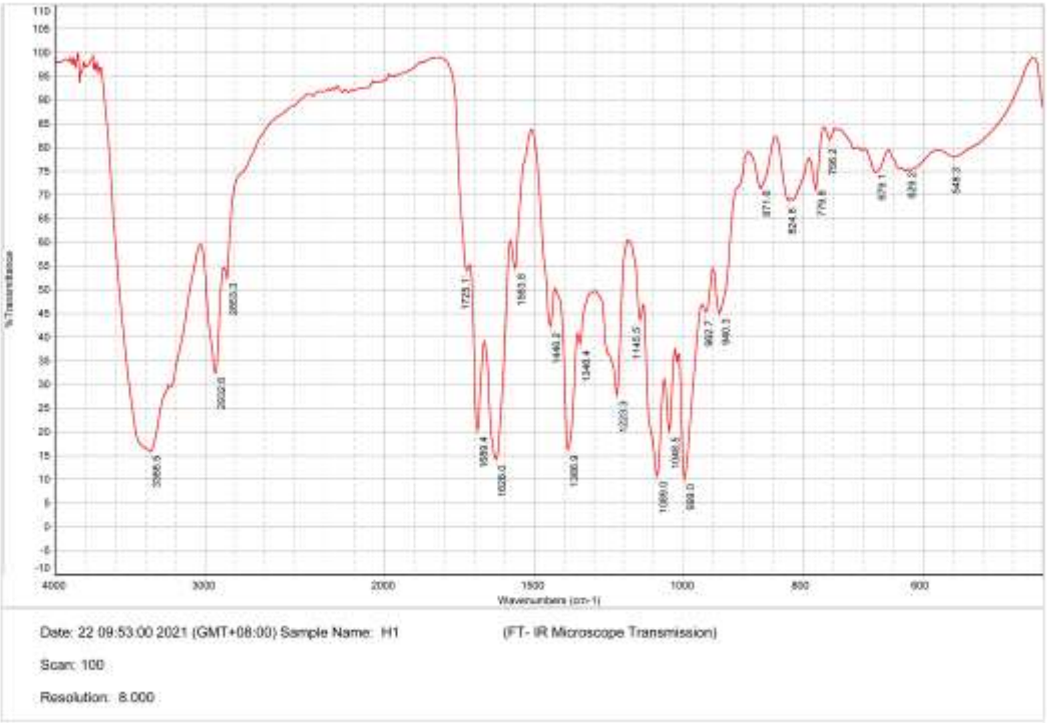

Figure S16. The IR spectrum of compound **16**

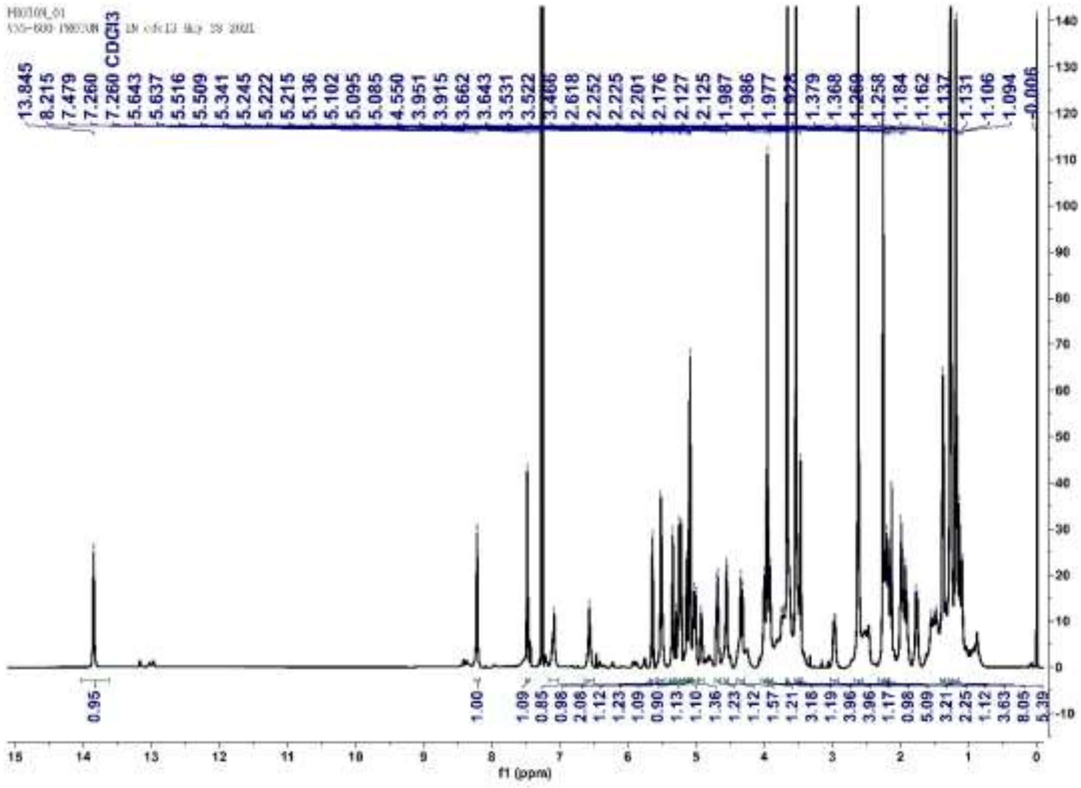

Figure S17. The ^{1}H NMR (600 MHz) spectrum of compound **16** in $CDCl_3$

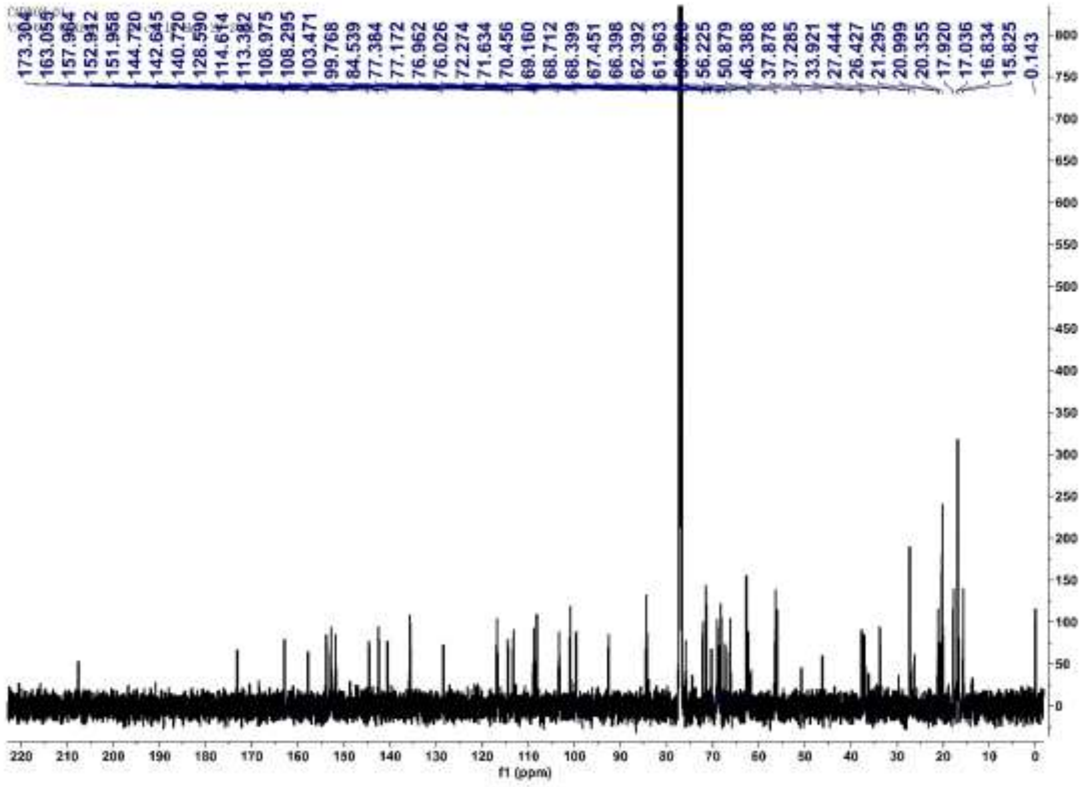

Figure S18. The ^{13}C NMR (150 MHz) spectrum of compound **16** in $CDCl_3$

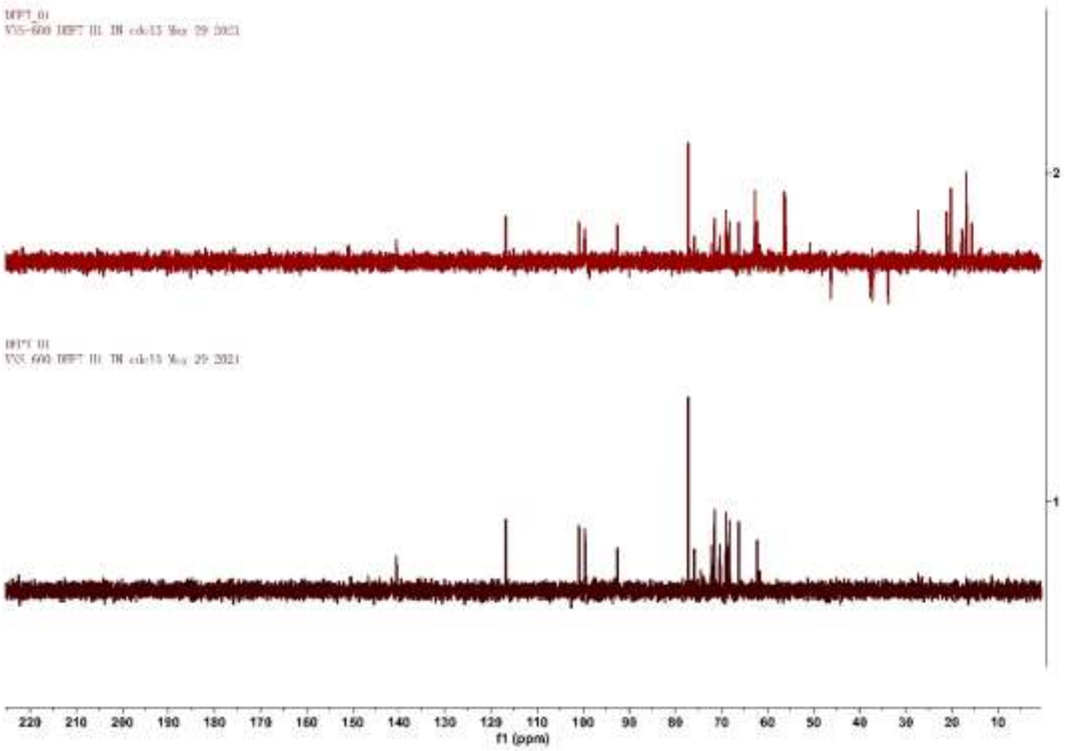

Figure S19. The DEPT spectrum of compound **16**

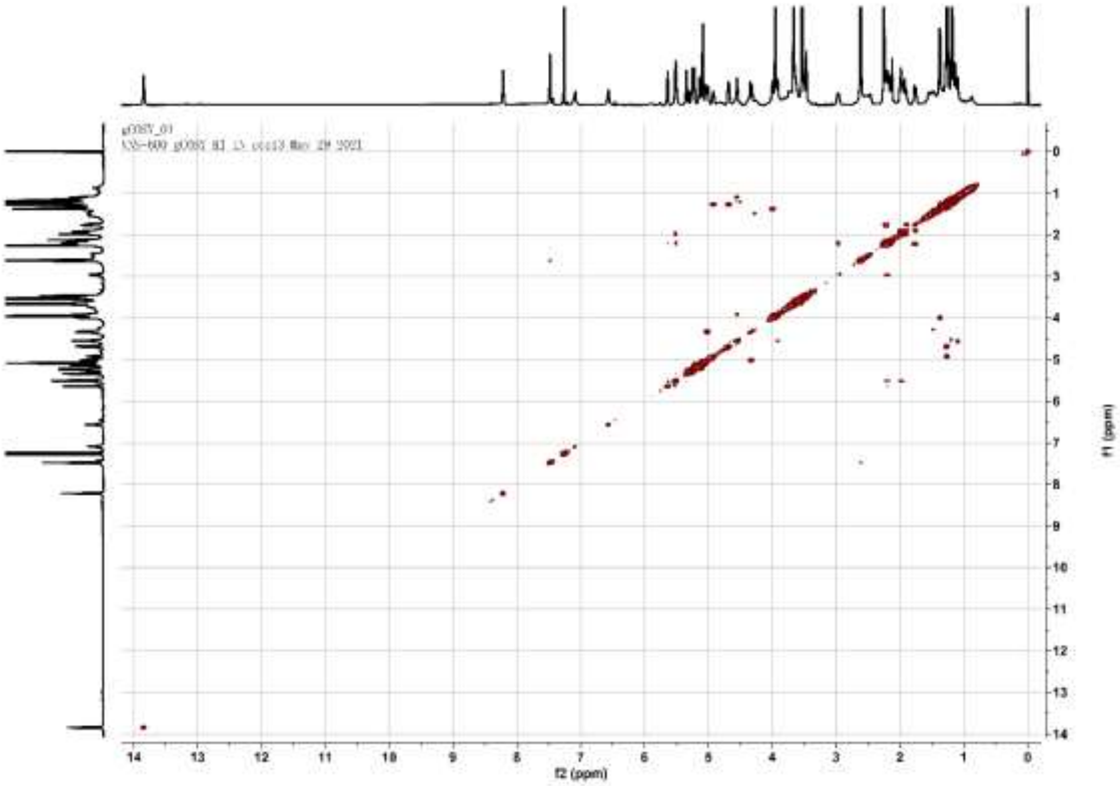

Figure S20. The ^{1}H-^{1}H COSY spectrum of compound **16**

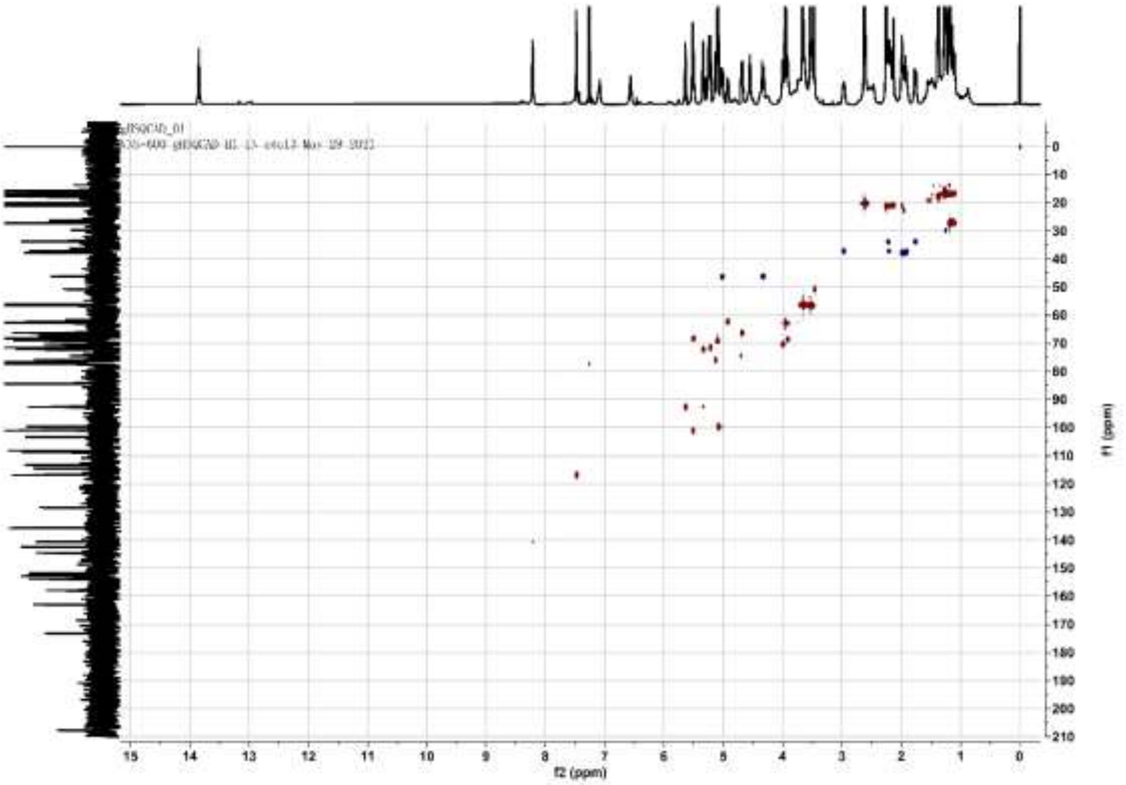

Figure S21. The HSQC spectrum of compound **16**

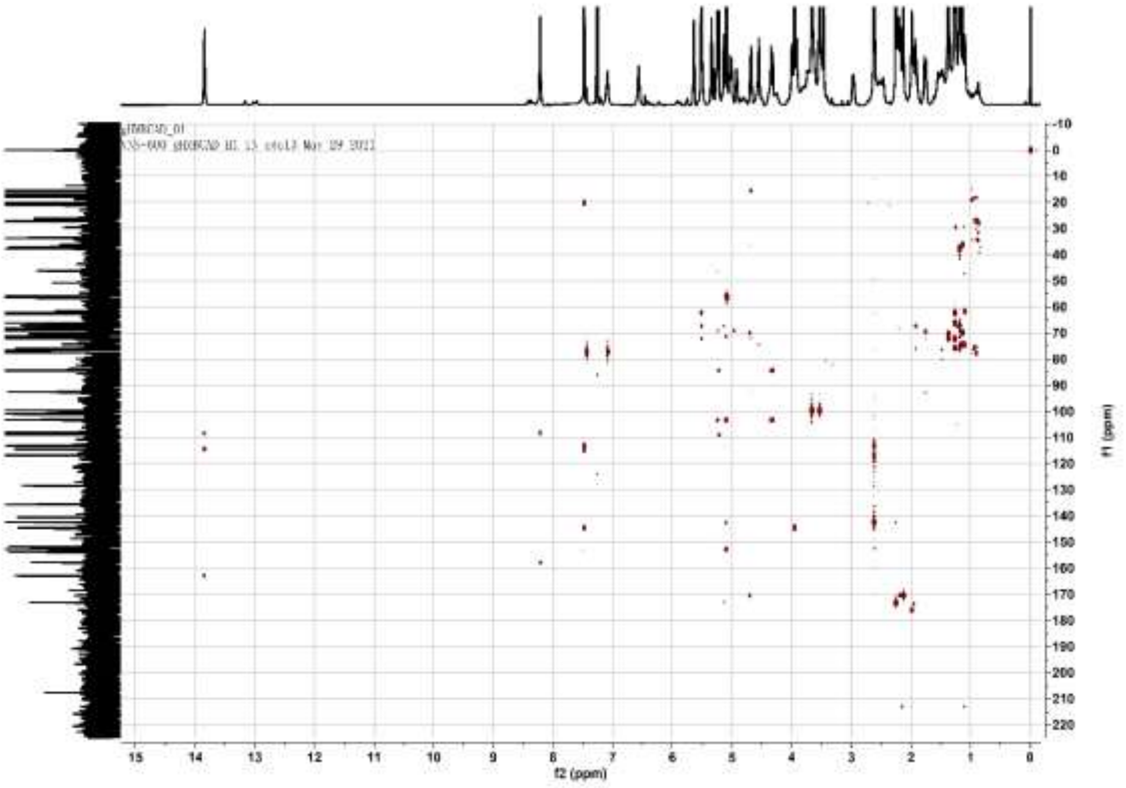

Figure S22. The HMBC spectrum of compound **16**

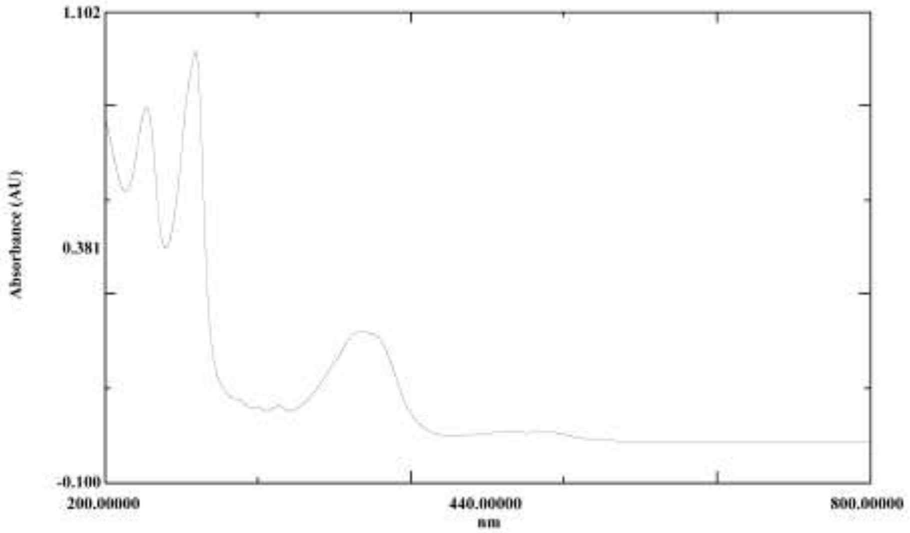

Figure S23. The UV spectrum of compound **20**

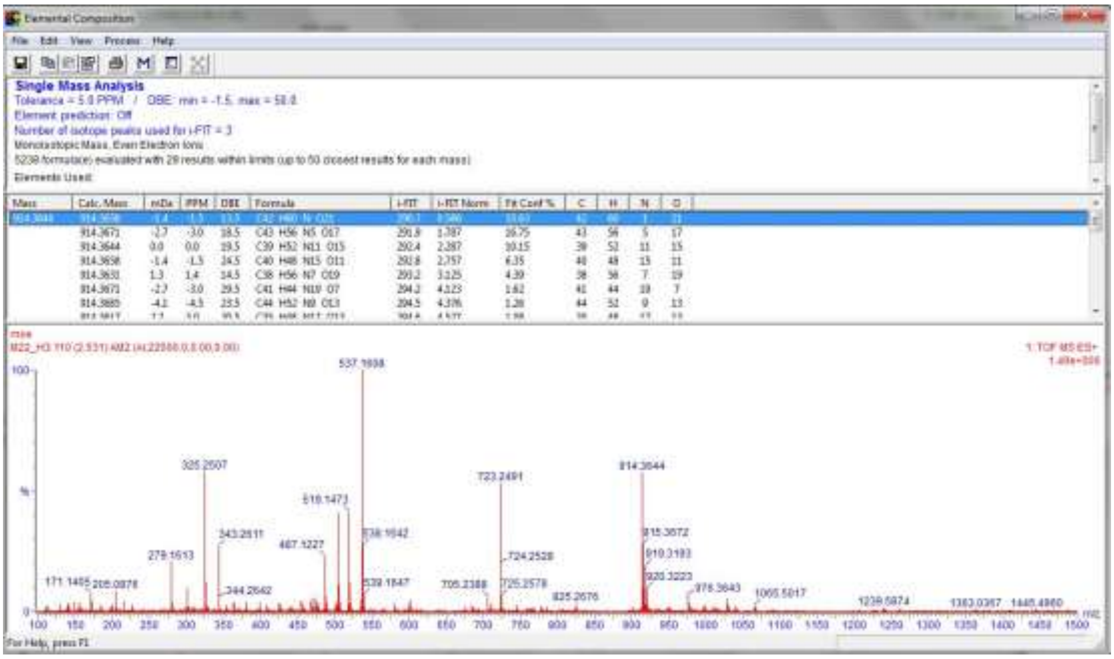

Figure S24. The HRESIMS of compound **20**

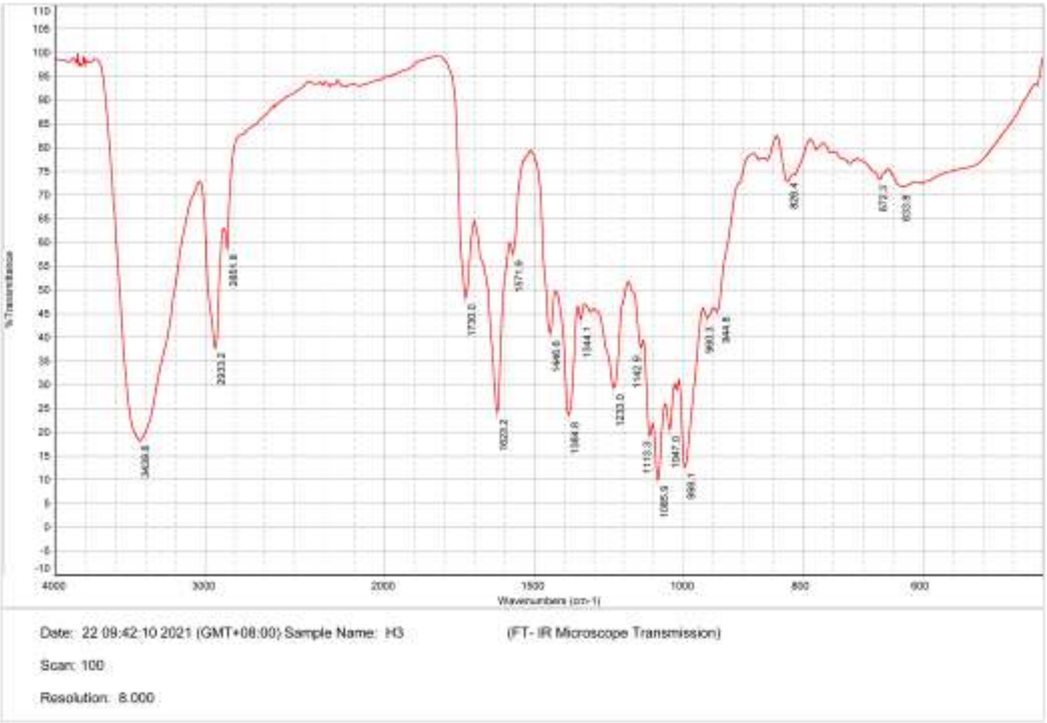

Figure S25. The IR spectrum of compound **20**

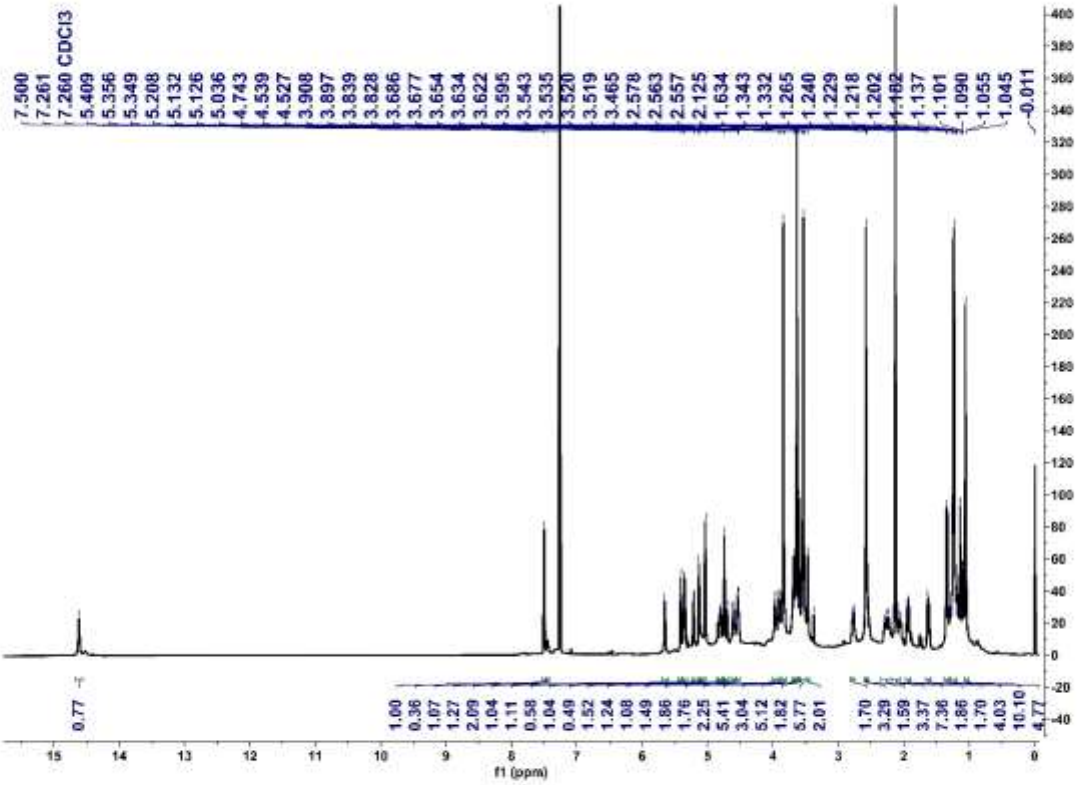

Figure S26. The ^{1}H NMR (600 MHz) spectrum of compound **20** in $CDCl_3$

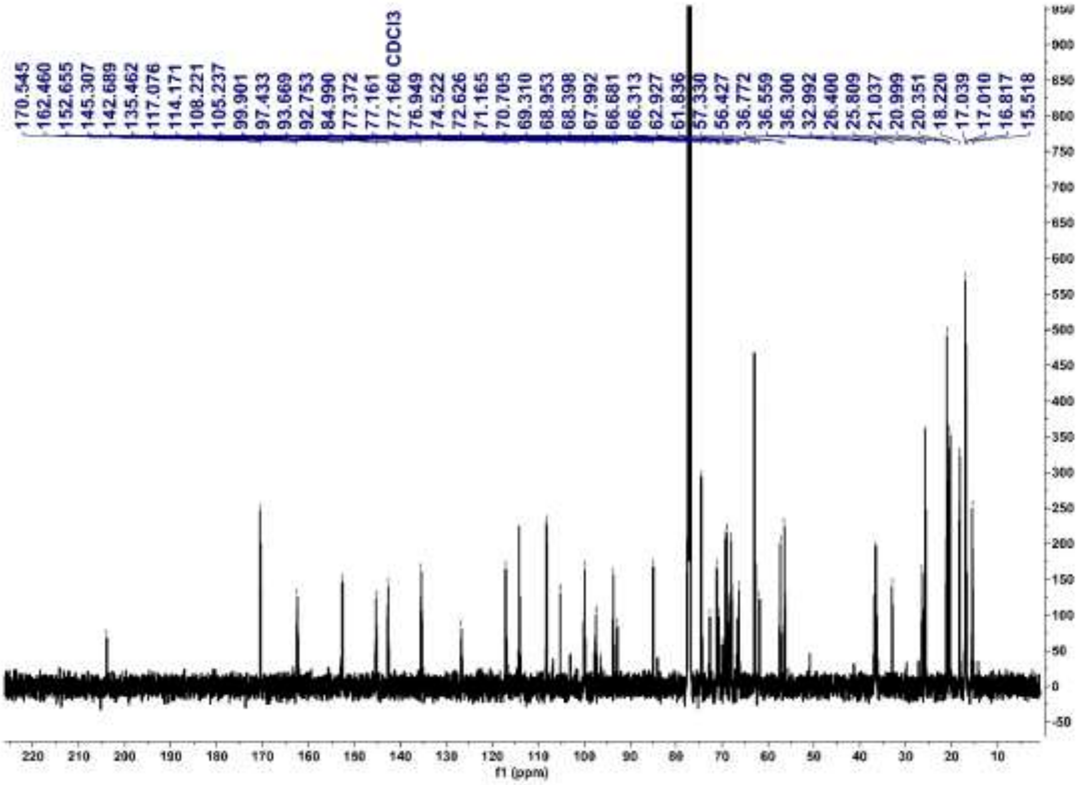

Figure S27. The ^{13}C NMR (150 MHz) spectrum of compound **20** in $CDCl_3$

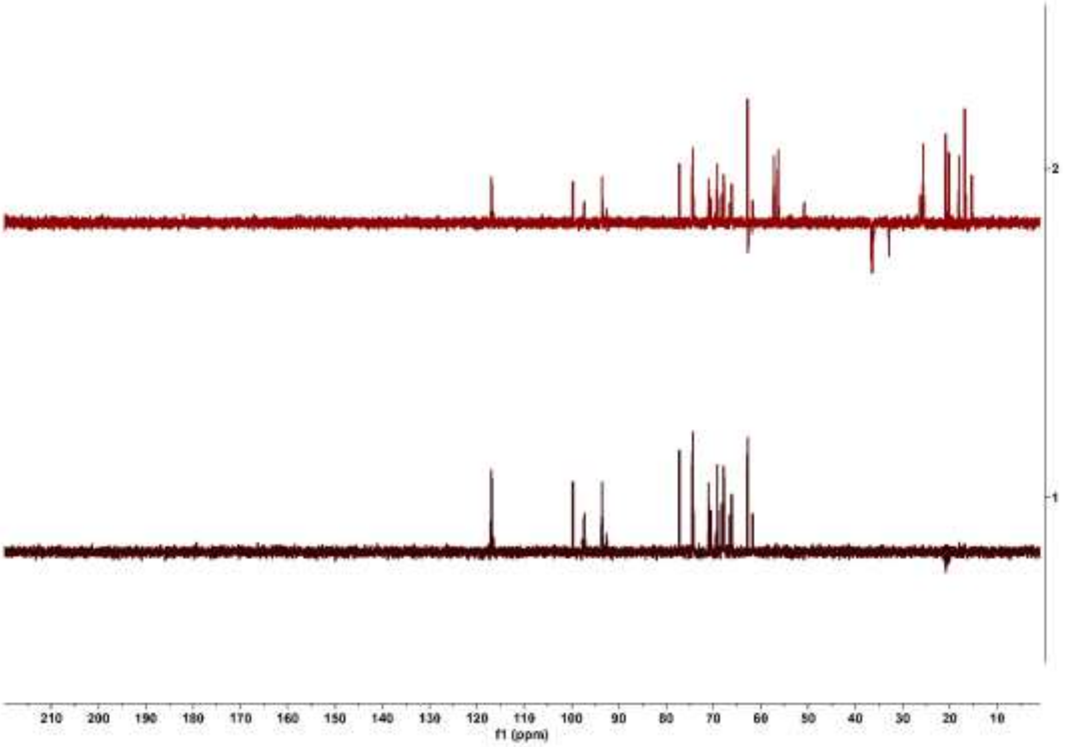

Figure S28. The DEPT spectrum of compound **20**

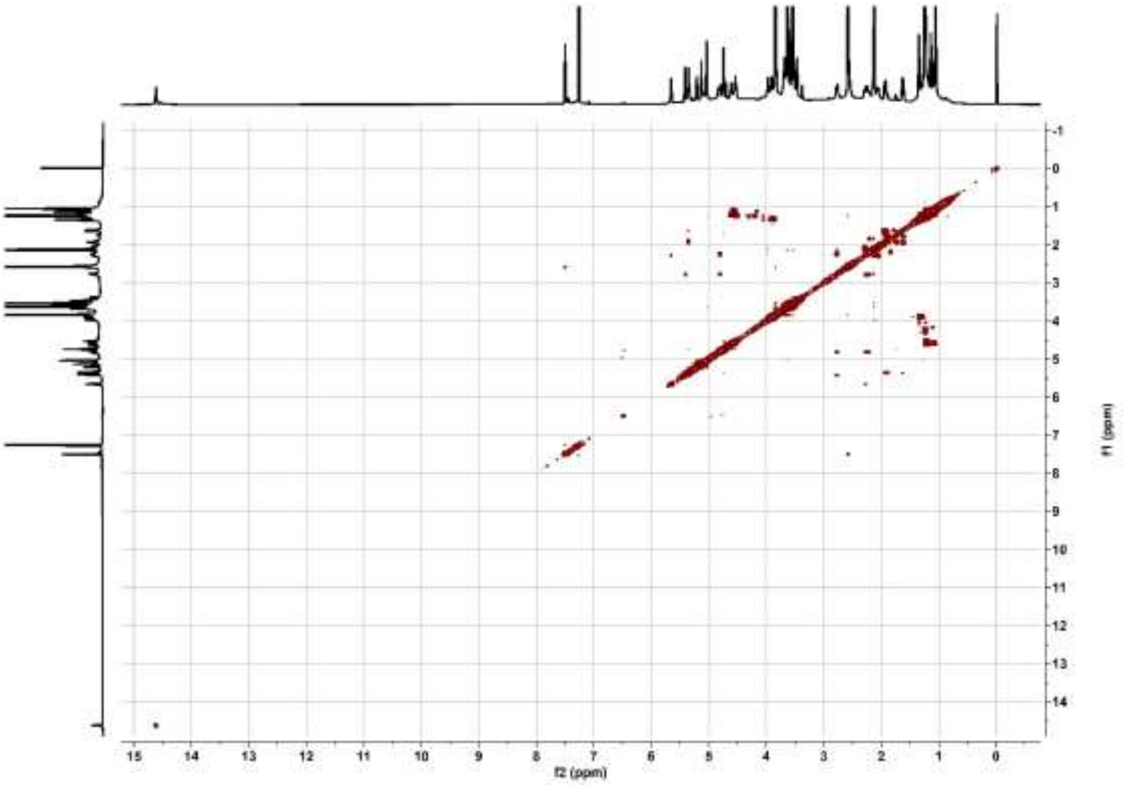

Figure S29. The ^1H-^1H COSY spectrum of compound **20**

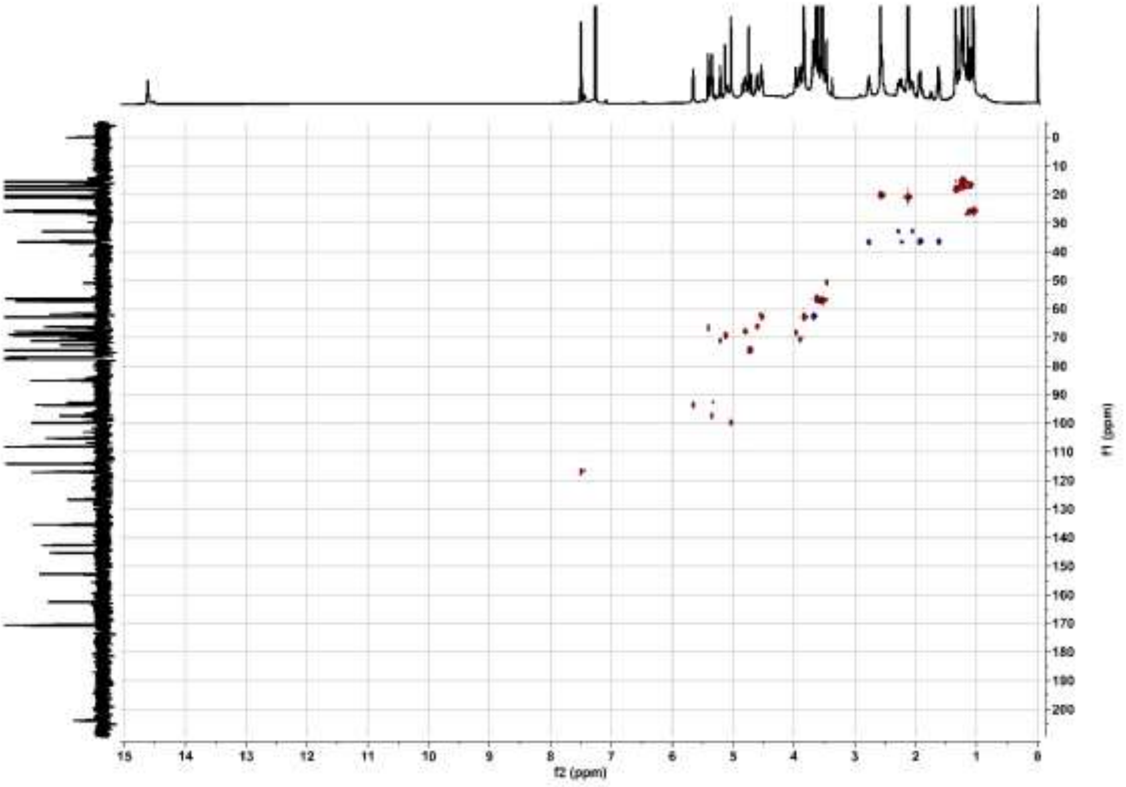

Figure S30. The HSQC spectrum of compound **20**

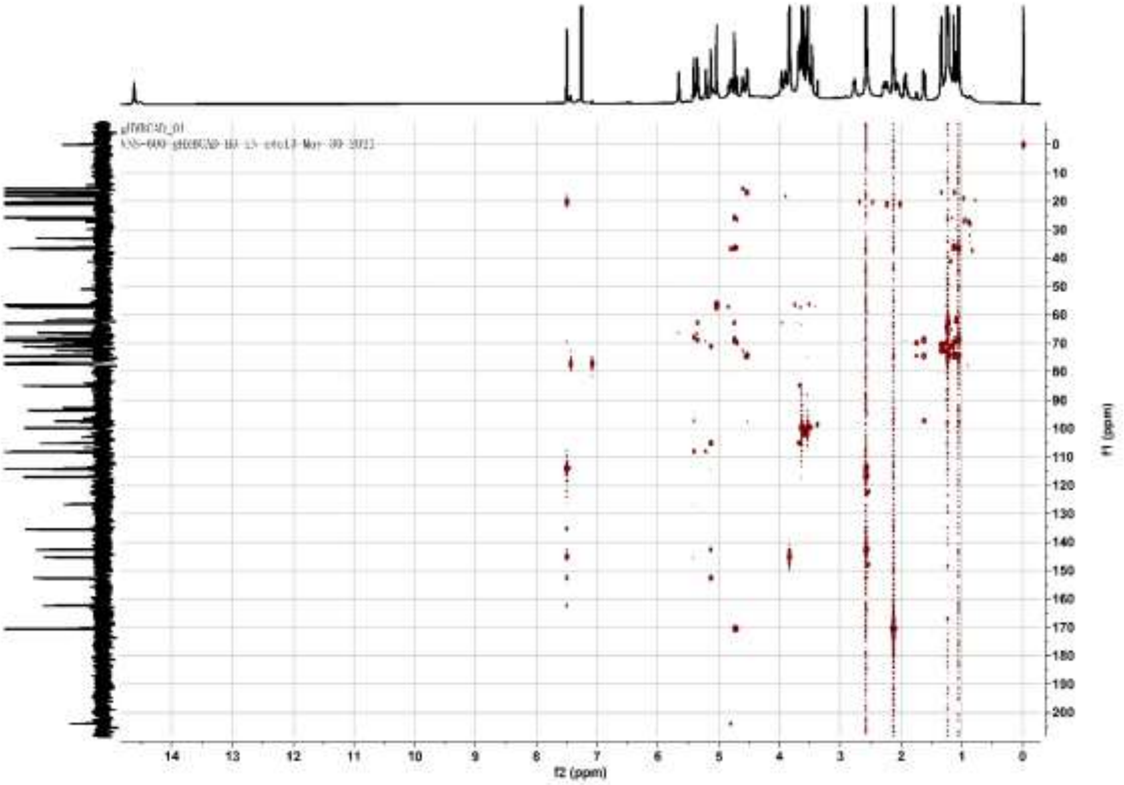

Figure S31. The HMBC spectrum of compound **20**

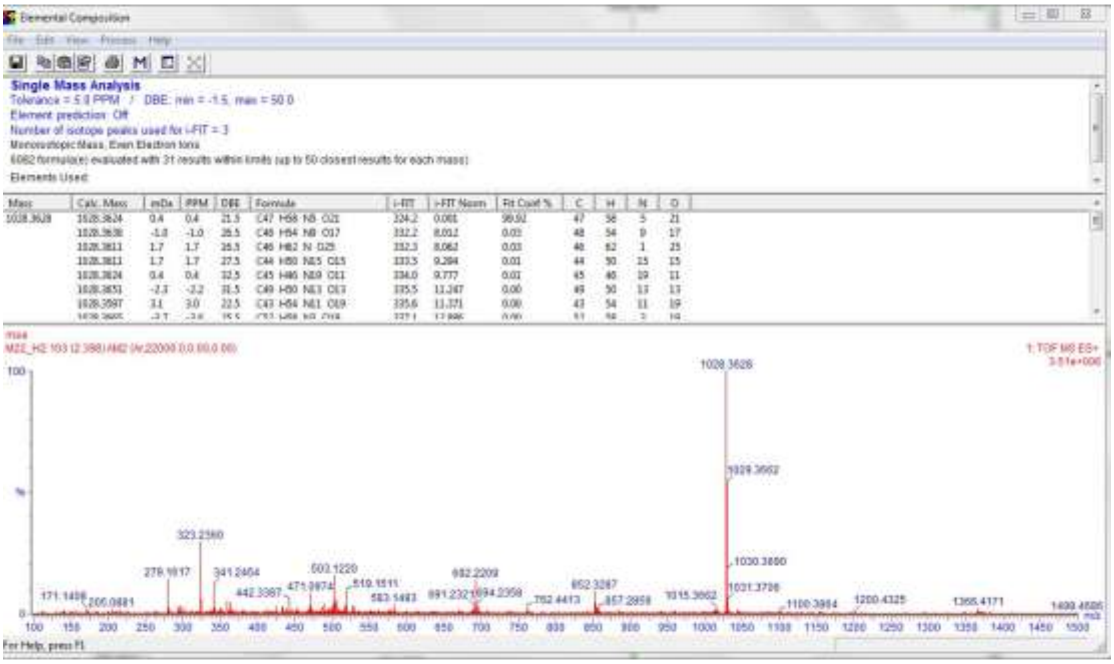

Figure S32. The HRESIMS of Compound **12**

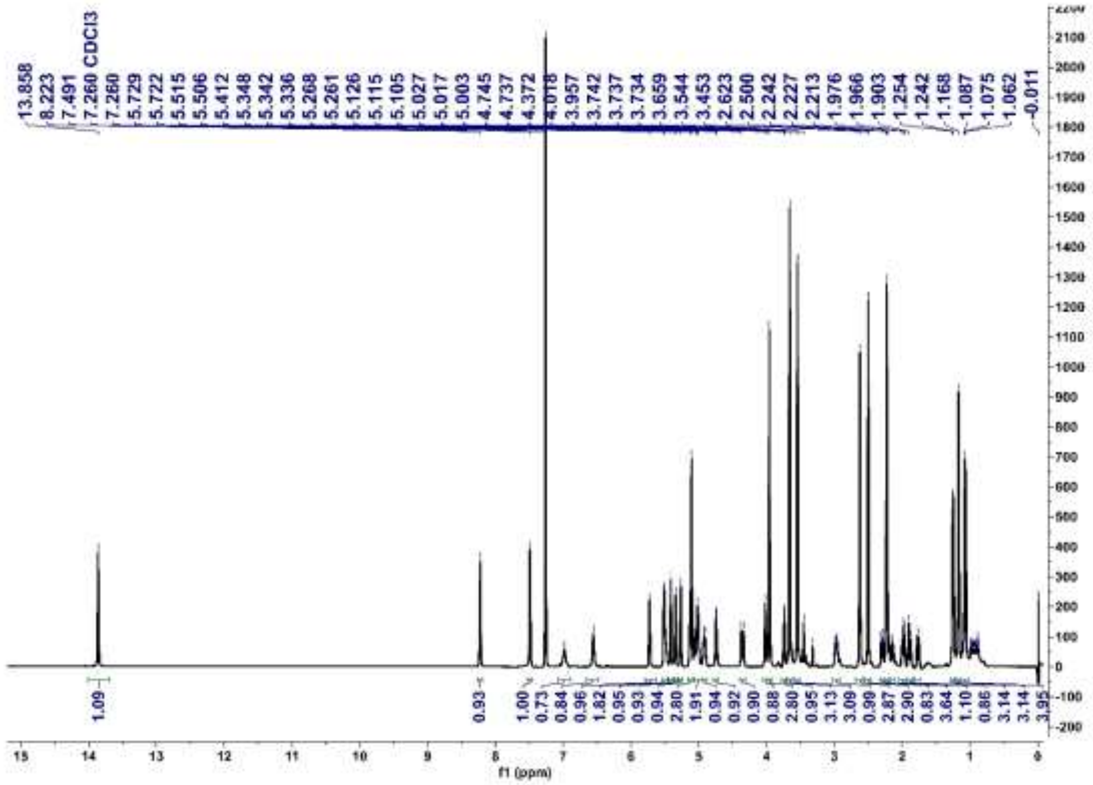

Figure S33. The ^{1}H NMR (500 MHz) spectrum of compound **12** in $CDCl_3$

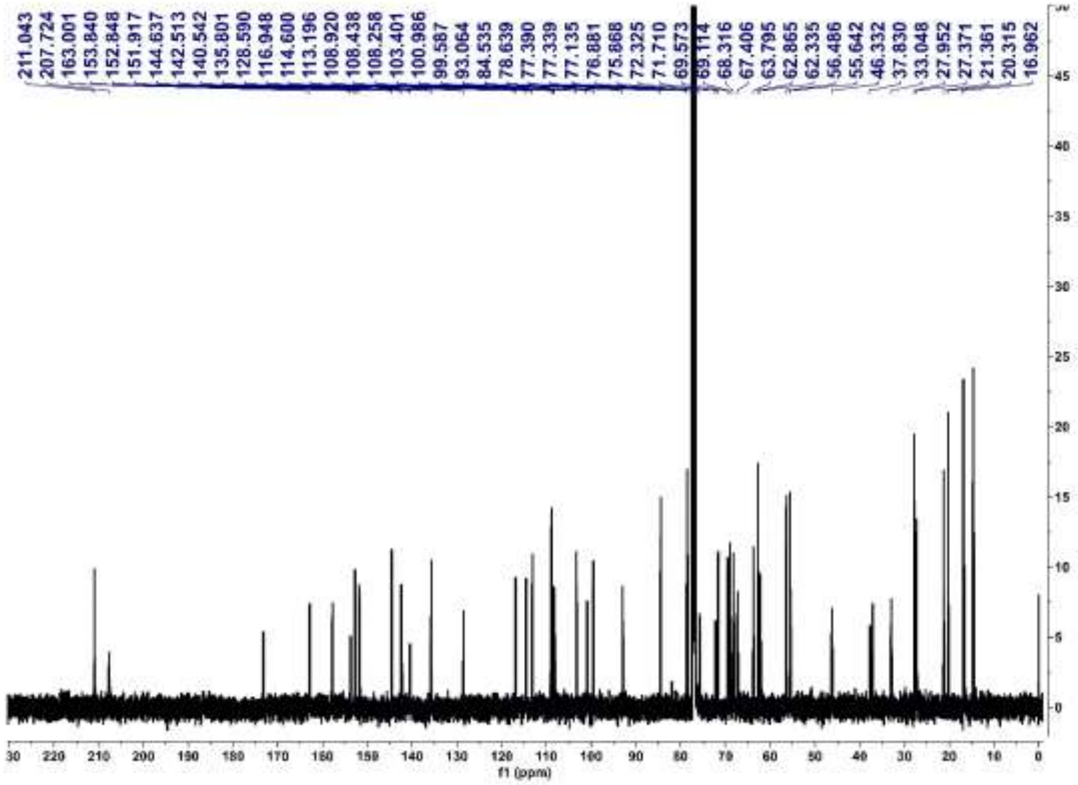

Figure S34. The ^{13}C NMR (125 MHz) spectrum of compound **12** in $CDCl_3$

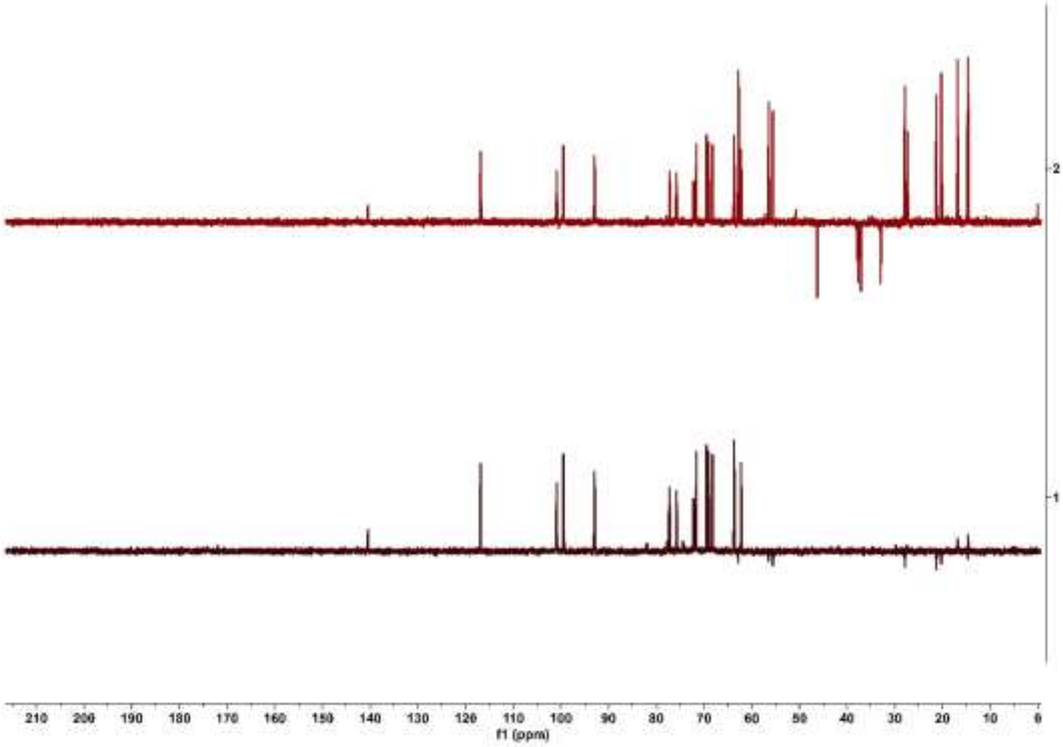

Figure S35. The DEPT spectrum of compound **12**

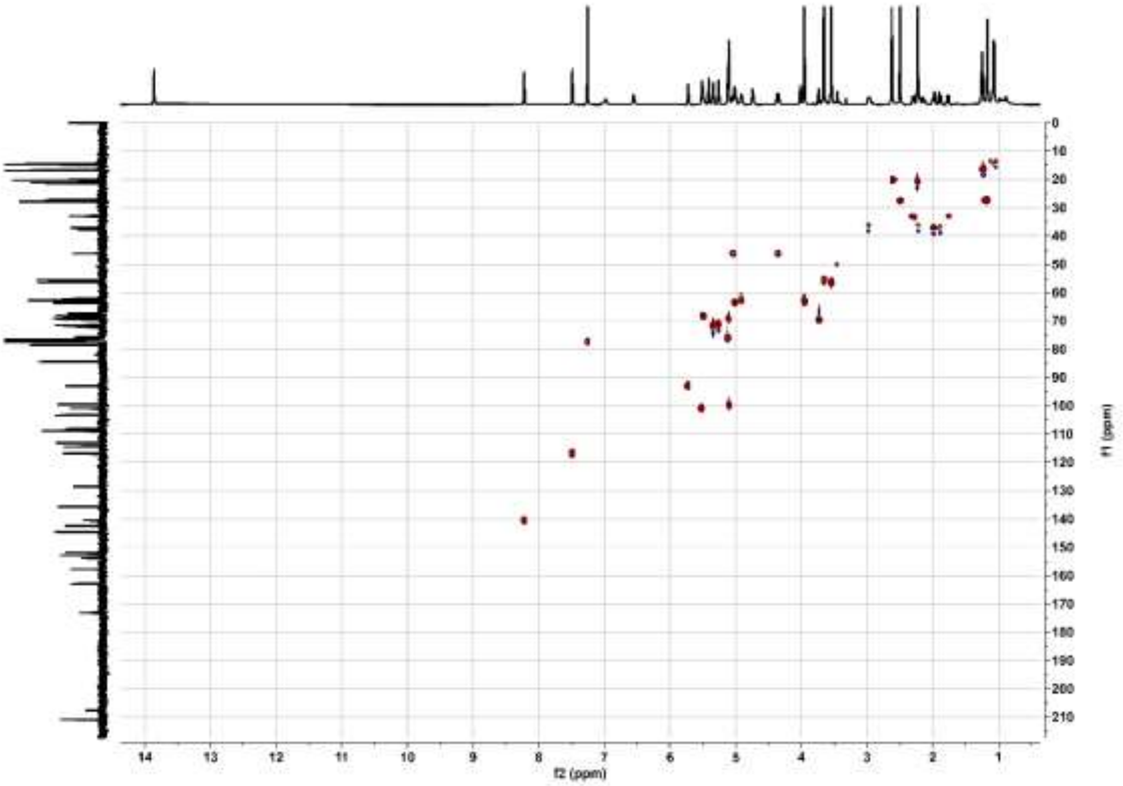

Figure S36. The HSQC spectrum of compound **12**

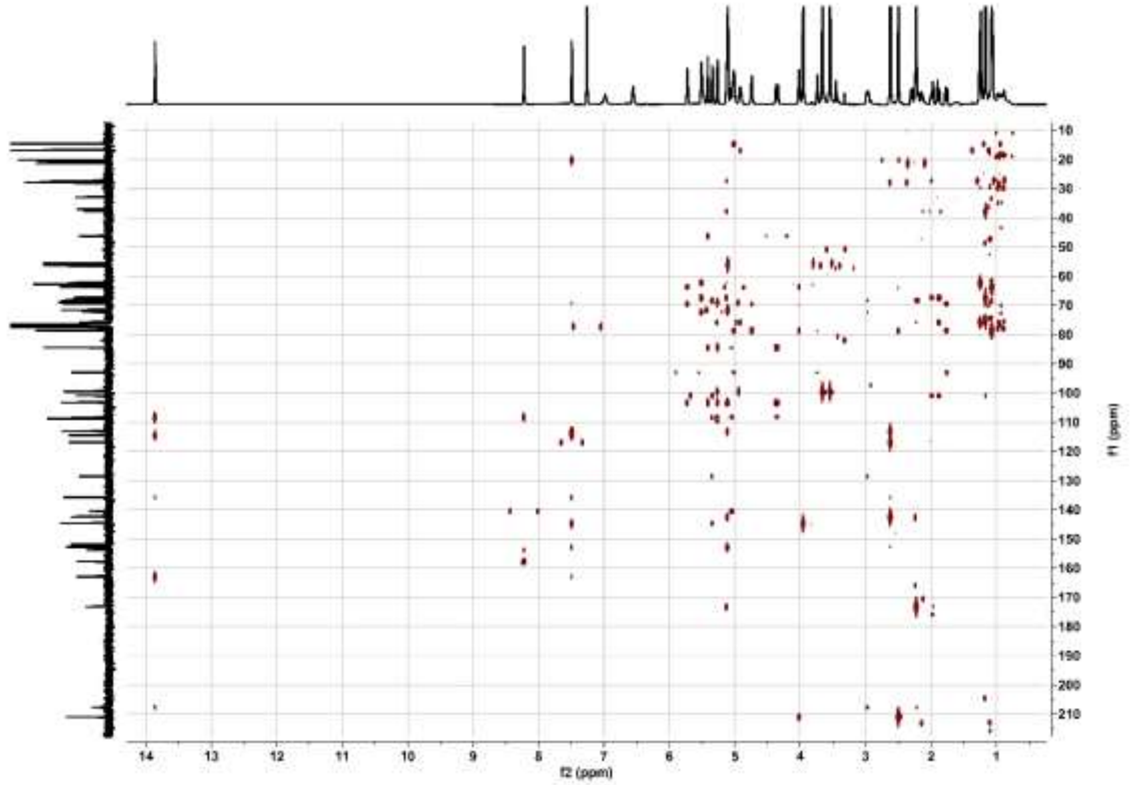

Figure S37. The HMBC spectrum of compound **12**

References

1. Lee, M.D.; Fantini, A.A.; Labeda, D.P.; Maiese, W.M.; Testa, R.T.; Borders, D.B. Antitumor agents LL-D49194α1, LL-D49194β1, LL-D49194β2, LL-D49194β3, LL-D49194γ, LL-D49194δ, LL-D49194ε, LL-D49194ξ, LL-D49194η, LL-D49194ω1, LL-D49194ω2, and LL-D49194ω3. Google Patents: 1986.
2. Maiese, W.M.; Labeda, D.P.; Korshalla, J.; Kuck, N.; Fantini, A.A.; Wildey, M.J.; Thomas, J.; Greenstein, M. LL-D49194 antibiotics, a novel family of antitumor agents: taxonomy, fermentation and biological properties. *J Antibiot (Tokyo)* **1990**, *43*, 253-258.
3. Maskey, R.P.; Helmke, E.; Fiebig, H.H.; Laatsch, H. Parimycin: isolation and structure elucidation of a novel cytotoxic 2,3-dihydroquinizarin analogue of gamma-indomycinone from a Marine *Streptomycete* isolate. *J Antibiot (Tokyo)* **2002**, *55*, 1031-1035.
4. Tamaoki, T.; Shirahata, K.; Iida, T.; Tomita, F. Trioxacarcins, novel antitumor antibiotics. II. Isolation, physico-chemical properties and mode of action. *J Antibiot (Tokyo)* **1981**, *34*, 1525-1530.
5. Maskey, R.P.; Helmke, E.; Kayser, O.; Fiebig, H.H.; Maier, A.; Busche, A.; Laatsch, H. Anti-cancer and antibacterial trioxacarcins with high anti-malaria activity from a marine *Streptomycete* and their absolute stereochemistry. *J Antibiot (Tokyo)* **2004**, *57*, 771-779.
6. Maskey, R.P.; Sevvana, M.; Usón, I.; Helmke, E.; Laatsch, H. Gutingimycin: a highly complex metabolite from a marine *Streptomycete*. *Angew. Chem. Int. Ed. Engl.* **2004**, *43*, 1281-1283.

7. Shirahata, K.; Iida, T. Compounds having antibiotic activity, processes for their preparation, pharmaceutical compositions containing them and their use as medicaments. Google Patents: 1984.
8. Nicolaou, K.C.; Cai, Q.; Sun, H.; Qin, B.; Zhu, S. Total Synthesis of Trioxacarcins DC-45-A1, A, D, C, and C7″-epi-C and Full Structural Assignment of Trioxacarcin C. *J. Am. Chem. Soc.* **2016**, *138*, 3118-3124.
9. Nicolaou, K.C.; Cai, Q.; Qin, B.; Petersen, M.T.; Mikkelsen, R.J.; Heretsch, P. Total synthesis of trioxacarcin DC-45-A2. *Angew. Chem. Int. Ed. Engl.* **2015**, *54*, 3074-3078.
